# Revisiting metazoan phylogeny with genomic sampling of all phyla

**DOI:** 10.1098/rspb.2019.0831

**Published:** 2019-07-10

**Authors:** Christopher E. Laumer, Rosa Fernández, Sarah Lemer, David Combosch, Kevin M. Kocot, Ana Riesgo, Sónia C. S. Andrade, Wolfgang Sterrer, Martin V. Sørensen, Gonzalo Giribet

**Affiliations:** 1 Museum of Comparative Zoology (MCZ) and Department of Organismic and Evolutionary Biology, Harvard University, 26 Oxford Street, Cambridge, MA 02138, USA; 2 EMBL-European Bioinformatics Institute (EBI), Wellcome Genome Campus, Hinxton CB10 1SD, UK; 3 Bioinformatics & Genomics Unit, Center for Genomic Regulation, Carrer del Dr. Aiguader 88, 08003 Barcelona (Spain); 4 Marine Laboratory, University of Guam, UOG Station, Mangilao, Guam 96923, USA; 5 Department of Biological Sciences and Alabama Museum of Natural History, The University of Alabama, Campus Box 870344, Tuscaoosa, AL 35487, USA; 6 Department of Life Sciences, Natural History Museum of London, Cromwell Road, London SW7 5BD, UK; 7 Departamento de Genética e Biologia Evolutiva, IB, Universidade de São Paulo, 05508090 São Paulo, SP, Brazil; 8 Bermuda Natural History Museum, PO Box FL 145, Flatts, FLBX, Bermuda; 9 Natural History Museum of Denmark, Universitetsparken 15, 2100 Copenhagen, Denmark

**Keywords:** phylogenomics, animal phylogeny, compositional bias, taxon sampling, matrix recoding

## Abstract

Proper biological interpretation of a phylogeny can sometimes hinge on the placement of key taxa—or fail when such key taxa are not sampled. In this light, we here present the first attempt to investigate (though not conclusively resolve) animal relationships using genome-scale data from all phyla. Results from the site-heterogeneous CAT + GTR model recapitulate many established major clades, and strongly confirm some recent discoveries, such as a monophyletic Lophophorata, and a sister group relationship between Gnathifera and Chaetognatha, raising continued questions on the nature of the spiralian ancestor. We also explore matrix construction with an eye towards testing specific relationships; this approach uniquely recovers support for Panarthropoda, and shows that Lophotrochozoa (a subclade of Spiralia) can be constructed in strongly conflicting ways using different taxon- and/or orthologue sets. Dayhoff-6 recoding sacrifices information, but can also reveal surprising outcomes, e.g. full support for a clade of Lophophorata and Entoprocta + Cycliophora, a clade of Placozoa + Cnidaria, and raising support for Ctenophora as sister group to the remaining Metazoa, in a manner dependent on the gene and/or taxon sampling of the matrix in question. Future work should test the hypothesis that the few remaining uncertainties in animal phylogeny might reflect violations of the various stationarity assumptions used in contemporary inference methods.

## 1. Background

For over a decade, molecular phylogeneticists have enjoyed the use of automated methods to use shotgun DNA sequencing data to decipher the deepest relationships in the animal tree of life [1,2]. This paradigm has continued the disruptive tradition of molecular phylogenetics, allowing the placement of taxa whose morphology and embryology have proven uninformative or misleading in this regard, and demonstrating that early animal evolution resulted in considerably more flexibility in phenotypic evolution than initially expected [3–5]. Unfortunately, however, even with the availability of genome-scale data, the shift away from morphologically defined trees has not proceeded towards one consistent molecular tree. Controversies have abounded, including some ongoing ones, such as the contention that Xenacoelomorpha represent deuterostomes [6–8], or the debate over the earliest split in the animal tree [9–16]. Furthermore, although highly parallel short-read sequencing has essentially overtaken Sanger and competing second-generation sequencing technologies, there is still a dearth of genomic data from several phyla (e.g. Bryozoa, Loricifera, Kinorhyncha and Nematomorpha), and it has been a decade since the last major synoptic attempt to infer the relationships among all animal groups [2], despite exemplary recent analyses focused on specific clades [17,18]. Indeed, it seems there has not yet been an attempt to investigate the animal tree of life using genome-scale data from representatives of all metazoan phyla. Herein, collating a mixture of published (prior to 2018) and new transcriptome and genome data sequenced largely with Illumina technology, and employing numerous strategies to control the influence of systematic error [19] and to build both general and taxon-specific matrices from a single orthology assignment, we empirically review the signals for and robustness of most animal clades recognized in the recent era.

## 2. Methods

Detailed description of molecular methods for RNA and DNA sequencing and run parameters for all bioinformatic analyses are provided as electronic supplementary material.

### (a) Orthologue assignment and matrix construction

**Figure 1.**
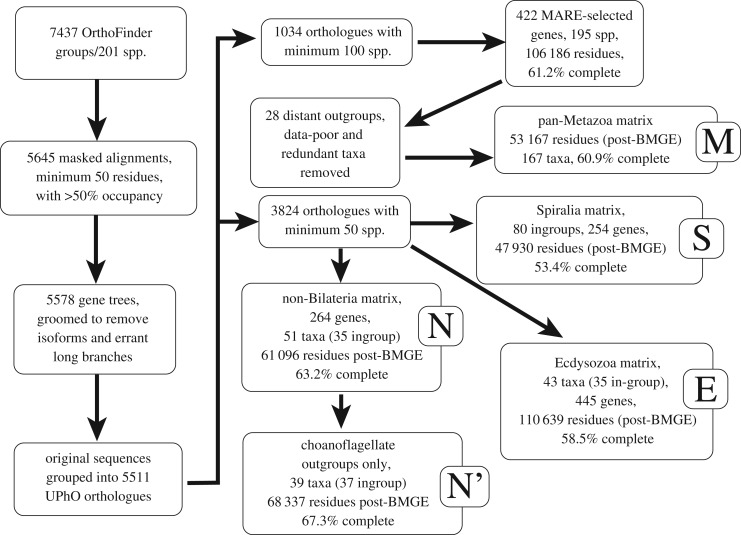
Schematic description of gene tree construction, orthologue assignment and matrix construction. Gene selection criteria for clade-specific matrix construction and other methodological details are discussed in the text.

Predicted proteomes derived from annotated genome and transcriptome assemblies were clustered into 7437 OrthoFinder groups (figure 1) comprising 201 spp. sampling all metazoan phyla (except for Orthonectida, from which no genomic resources were available at the time of this work's inception [20,21], and which were recently shown to represent modified annelids [22]), plus a variety of opisthokont outgroups (electronic supplementary material, table S1). From these, we constructed 5578 maximum-likelihood (ML) gene family trees from a subset of well-aligned groups, and processed these to mask candidate redundant isoforms, remove isolated divergent sequences and further split trees into subfamilies subtended by long internal branches [23]. These groomed gene trees were parsed to extract 5511 orthologues by the criterion of unrooted phylogenetic orthology (UPhO) [24].

To construct a single supermatrix representing all Metazoa, we considered the set of 1034 orthologues with 100 or more representatives. This was done in part to ensure a matrix with high taxon occupancy, but also to limit the effects of cryptic horizontal gene-transfer, biological cross-contamination or index misassignment (of which libraries produced in this laboratory have, however, previously shown little evidence [25]): when selecting orthologues by parsing gene family trees with the species overlap algorithm, as done here, such processes should tend to split large orthologues into smaller groupings. From this set of 1034, we further reduced to 422 information-rich genes present in 195 taxa (figure 1). Our initial ML tree showed evidence of redundant and poorly placed individual taxa, as well as the presence of some clades previously shown to be driven by compositional bias (e.g. Polyzoa [26]; electronic supplementary material, figure S1). We therefore selected a set of 28 taxa to remove (see electronic supplementary material, table S1), including all non-choanoflagellate outgroups, following contemporaneous suggestions of compositionally driven effects from the inclusion of these clades [11,12], and trimmed the matrix of putatively saturated and compositionally biased sites with the BMGE tool [27]. Interestingly, this procedure reduced the matrix from an initial 106 186 sites to 53 167 sites when these taxa were removed prior to BMGE, but to only 43 011 sites when all taxa are included prior to BMGE-trimming, indicating that more sites are detected as compositionally heterogeneous when distant outgroups are included in the test; we focus results from the 53 167-site matrix (M), but refer to those from the shorter matrix for comparison (see the electronic supplementary material figures).

We also constructed subclade-specific matrices with more limited taxon sampling (figure 1), meant to test specific relationships within these major subclades (e.g. Ecdysozoa, Spiralia), and with orthologues selected from within the set of 3824 with greater than 50 sequences each, to optimize representation of the clades in question. A 43-taxon (34 ingroups) Ecdysozoa matrix (E) was prepared with a MARE-reduced subset of 445 genes, from the set of those which had at least two representatives each of Kinorhyncha, Loricifera or Nematomorpha. An 80-taxon matrix (S) was constructed to test the position of Cycliophora and Entoprocta within Spiralia, composed of the 254 genes with representation of at least three each of Entoprocta + Cycliophora (no ecdysozoan outgroups were included); although relationships within Spiralia have been controversial in many respects, the position of Entoprocta and Cycliophora within this clade has been among the most difficult to assess, potentially due to compositional bias [17,26,28]. Indeed, an extensive recent analysis of Spiralian relationships [18] chose to avoid solving the position of Cycliophora entirely by trimming this group (and many other compositionally biased species) away entirely prior to phylogenetic inference. Here, we have favoured including all taxa possible, given the recognized importance of taxon sampling in accurate phylogenetic inference, choosing to mitigate compositional heterogeneity by trimming problematic alignment sites rather than entire taxa. A 51-taxon matrix (N) was finally constructed to evaluate relationships between Bilateria and the remaining four animal phyla and outgroups, including 264 genes selected by MARE from within the set of alignments that included the placozoan and at least one choanoflagellate, six sponges, three ctenophores and four cnidarians. We also constructed a version of this matrix (N′) with all non-choanoflagellate outgroups removed, leaving 39 taxa; BMGE-trimming was performed after taxon deletion, yielding 68 337 and 61 096 residues for the 51 and 39 taxon matrices, respectively. These matrices allowed us to test whether a metazoan-wide matrix and those that are clade-specific and therefore more informative to the specific question, produce comparable results.

### (b) Phylogenetic inference

We present principally results from Bayesian inference under the CAT + GTR + *Γ*4 model, which has been shown both theoretically and empirically to suppress long-branch attraction artefacts in heterogeneous matrices such as the ones presented here [29–31]. A minimum of four chains per matrix were run in PhyloBayes-MPI v. 1.6j for up to 1.5 years. Straightforward posterior consensus formation with conventional burn-ins indicated difficulties achieving convergence in many matrices, even following such long computation times, but we preferred, in contrast to some recent work [8,13], to exhaustively investigate the signal within one informative matrix (rather than jackknifing within a larger matrix [19]), and to employ the more general CAT + GTR + *Γ*4 model over the CAT + *Γ*4, which has been shown to be more susceptible to systematic error [32]. However, we determined that the apparent difficulties in achieving acceptable metrics of convergence were principally the result of isolated rogue taxa in 3 poorly taxonomically sampled ecdysozoan groups present in each matrix (annotated in electronic supplementary material, table S1). Sometimes, such taxa were represented sparsely in each matrix (e.g. only 1009 and 1554 occupied sites for the two kinorhynch species represented in the pan-Metazoa matrix); however, other rogue taxa (particularly our single representative each of Nematomorpha and Loricifera) were represented in thousands of positions and nonetheless showed poor stability throughout CAT + GTR + *Γ*4 chains. Therefore, such rogue taxa, defined anew for each individual analysis, were masked prior to posterior consensus formation, resulting in acceptable (maxdiff < 0.2) maximum bipartition differences across chains and generally higher support values throughout, as has been seen previously [33]. Unpruned consensus summaries, fully labelled trees and ML analyses of each matrix are also presented in the electronic supplementary material which also includes a number of early analyses not further discussed (but described in caption, electronic supplementary material, figures S1–S24).

## 3. Results and discussion

### (a) Pan-metazoan matrix (M)

**Figure 2.**
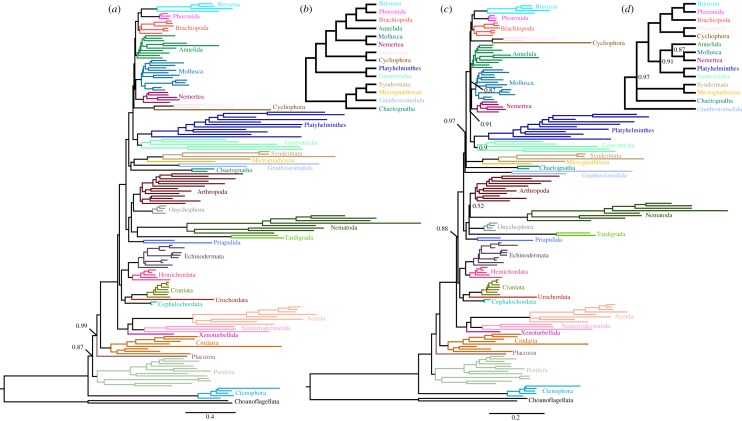
(*a*) Posterior consensus summary of CAT + GTR + *Γ*4 analysis of reduced, BMGE-trimmed pan-Metazoa matrix in amino acid space, trimmed of four rogue taxa prior to summary. (*b*) Cladogram depiction of relationships and support within Spiralia recovered in the previous analysis, shown to improve readability. (*c*) Posterior consensus summary of CAT + GTR + *Γ*4 analysis of the same matrix, recoded into Dayhoff-6 groups and trimmed of six rogue taxa prior to summary. (*d*) Cladogram depiction of relationships and support within Spiralia recovered in the previous analysis. Nodal support values are posterior probability; unlabelled nodes received full support. Relationships within labelled clades (phyla) are not annotated with support values to improve visualization, except in the case that the monophyly of the clade in question received less than full posterior probability. (Online version in colour.)

In broad structure, the trees from both amino acid and Dayhoff-6 group CAT + GTR + *Г*4 analyses of our most heavily analysed pan-metazoan matrix (figure 2), the BMGE-trimmed 53 167-site matrix M, recapitulate many deep relationships seen in molecular studies to date: Parahoxozoa, Planulozoa (= Cnidaria + Bilateria), Bilateria, Nephrozoa, Deuterostomia, Protostomia, Ecdysozoa and Spiralia all receive strong support. Ctenophora is recovered as the sister group of the remaining Metazoa, as is seen in many analyses [9]; however, unlike most other analyses to date, support for this split is poor in the amino acid analysis (figure 2*a,b*). Furthermore, contradicting previous observations that Dayhoff-6 group recoding tends to favour Porifera as the sister group to the remaining Metazoa, at least with this specific matrix, recoding instead maximizes support for Ctenophora in this position, even with only choanoflagellates as outgroups [11] (figure 2*c,d*). B**y** contrast, an even more heavily reduced (43 011-site) version of matrix M, where taxon (particularly, outgroup) deletion was applied only after trimming putatively saturated and/or compositionally biased sites, still shows poor resampling support for the earliest bipartition in Metazoa in CAT + GTR analysis at the amino acid level (electronic supplementary material, figure S3). Here, however, Placozoa are recovered with full support as the sister group of Cnidaria (a result also recently reported elsewhere [34]).

The more common effect of Dayhoff-6 recoding in matrix M is to reduce support for many clades, exemplified within Ecdysozoa. In the amino acid analysis (figure 2*a*), strong support is seen for an arthropod–onychophoran clade, as well as for a tardigrade–nematode clade, both of which are common outcomes of ecdysozoan molecular phylogenies [35–37], although the latter contradicts the clade Panarthropoda, also recovered in some genome-scale phylogenies [35,38]. Support for both of these clades is eroded under Dayhoff-6 recoding (figure 2*c*); indeed, the only ecdysozoan split for which strong support remains robust to this reduced alphabet is the division between Priapulida and the remaining Ecdysozoa. Unfortunately, within this analysis, our only representatives of the phyla Kinorhyncha, Nematomorpha and Loricifera behaved as rogue taxa (electronic supplementary material, figure S15–S17), and were therefore masked prior to posterior consensus summary. Ecdysozoa, one of the best-supported metazoan clades, therefore continues to be poorly resolved and understood [37].

Within Spiralia, our thorough taxon sampling permits interrogation of relationships within this challenging clade. Our results resemble most recent studies in recovering a mostly macrofaunal clade (Lophotrochozoa), sister group to a flatworm–gastrotrich clade (sometimes termed Rouphozoa [39]), both of which are sister to a clade populated by Gnathifera (Gnathostomulida, Micrognathozoa and Syndermata, inclusive of Rotifera and Acanthocephala) [17,28,39,40]. These results, however, differ markedly from a recent analysis also focusing on spiralian relationships, which found Rouphozoa to be non-monophyletic, with Gastrotricha and Platyhelminthes nested separately within Lophotrochozoa [18]. The contrasting approaches taken to mitigate compositional bias in this paper and our own (see Methods) may underlay this discrepancy. However, our analyses are in agreement with this paper in also finding Gnathifera as the sister group of Chaetognatha with full support in the displayed posterior consensus summary (figure 2). We emphasize, however, that support for this split is obtained only when our rogue ecdysozoan taxa are masked; in the unmasked posterior consensus (electronic supplementary material, figure S6), the loriciferan *Armorloricus elegans* is unexpectedly recovered within Gnathifera, as the sister group to Syndermata + Micrognathozoa, albeit its general instability breaks support for this relationship, as well as support for more basal nodes from this point down to the origin of Protostomia. This effect seems idiosyncratic to CAT + GTR + *Γ*4 analysis of this particular amino acid matrix, as it is not observed in the conjugate Dayhoff-6 recoded analysis of this matrix (electronic supplementary material, figure S8), in ML analysis of the same matrix (electronic supplementary material, figure S5), or in CAT + GTR + *Γ*4 analyses of the post-BMGE taxon-pruned version of this matrix (electronic supplementary material, figures S3 and S4). However, even when rogue taxa are masked, recoding modifies support for Chaetognatha as sister group to a monophyletic Gnathifera, although all constituent groups are still recovered as spiralians falling outside the clade formed by Platyhelminthes, Gastrotricha and Lophotrochozoa (figure 2*c*). The new relationship between Gnathifera and Chaetognatha is thus supported here, and in all the analyses of Marlétaz *et al*. [18], and was anticipated based on Hox presence data in Rotifera and Chaetognatha [41]. It has also been endorsed by the homology of the jaw elements of chaetognaths to those of the Middle Cambrian Burgess Shale fossil *Amiskwia sagittiformis*, which has a chaetognath-like body plan, but a jaw apparatus reminiscent of gnathiferans [42,43]. In view of the alternating support for Chaetognatha as the sister group to Gnathifera versus being nested within this clade—a duality found both by Marlétaz *et al*. [18] and ourselves in different analyses—we view the decision to declare chaetognaths as crown-group gnathiferans as premature, if not necessarily incorrect.

With adequate transcriptome representation from Entoprocta, Cycliophora, and with greatly improved sampling in Bryozoa, we see remarkable dynamics concerning the relative positions of these groups under different analytical conditions. Even trimmed of many compositionally biased sites, with ML analysis (electronic supplementary material, figures S2 and S10) we recover support for Polyzoa [2] (Entoprocta + Cycliophora + Bryozoa), a grouping previously suspected to represent a compositional artefact [18,26,40,44] as sister group to the remaining Lophotrochozoa. In CAT + GTR + *Γ*4 analysis of the amino acid matrix (figure 2*a*), we instead see Bryozoa placed within a lophophorate clade, as the sister group to Phoronida, with Entoprocta + Cycliophora forming the sister group to Lophotrochozoa. However, CAT + GTR + *Γ*4 analysis of recoded Dayhoff-6 groups yields yet a third possibility, to our knowledge not yet recovered in any molecular phylogeny—a clade of Entoprocta + Cycliophora sister group to a monophyletic Lophophorata, although the position of this clade within Spiralia at large is uncertain in this recoded analysis. On the contrary, Marlétaz *et al*. [18] found Entoprocta to be the sister group of Mollusca, recovering the traditional clade Lacunifera, based on the supposed haemocoel of entoprocts that has been interpreted by some authors as a lacunar circulatory system, similar to that of molluscs [45,46]. Prior analyses have shown that the position of Entoprocta is highly dependent on the presence or absence of Cycliophora, which was pruned prior to analysis in the study of Marlétaz *et al*. [18].

### (c) Spiralian matrix (S)

**Figure 3.**
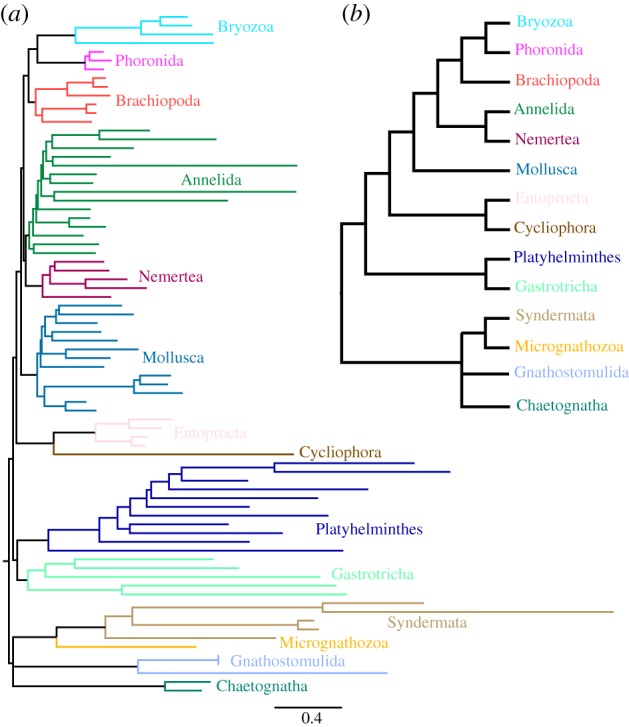
(*a*) Posterior consensus summary of CAT + GTR + *Γ*4 analysis of BMGE-trimmed Spiralia-specific matrix in amino acid space, trimmed of 2 rogue taxa prior to summary; the phylogram is drawn with the position of the root taken from the pan-Metazoa results shown in figure 2*b*. Cladogram depiction of the same, given to improve readability. Criteria for nodal annotation are as in figure 2; in this case, no internodes outside the labelled phyla received less than full support.

A parallel CAT + GTR + *Γ*4 analysis of a separate matrix constructed to optimize representation of Entoprocta + Cycliophora, in particular, provides another test of spiralian relationships. Amino acid level results (figure 3) are remarkably similar to the picture seen in the pan-Metazoa matrix, from which the position of the root in this outgroup-lacking tree is taken. Indeed, the only major differences are the lack of support for a monophyletic Gnathifera in this analysis (as it includes Chaetognatha, a result also sometimes found by Marlétaz *et al*. [18]), and relationships within Lophotrochozoa, here with nemerteans and annelids forming a sister group to the lophophorates, with molluscs branching immediately prior to this clade, whereas in the pan-Metazoa analysis (figure 2*a*) molluscs and nemerteans constitute the sister group to an annelid–lophophorate clade. Both of these scenarios differ from the result recovered by a similar CAT + GTR + *Γ*4 analysis published in 2015 [40]. Dayhoff-6 recoding in this matrix, in contrast to the pan-Metazoa matrix, does not recover a clade of Lophophorata and Entoprocta + Cycliophora; instead, the overall topology is identical to that of the amino acid matrix but in this relatively small matrix support diminishes throughout (electronic supplementary material, figure S13).

### (d) Ecdysozoan matrix (E)

**Figure 4.**
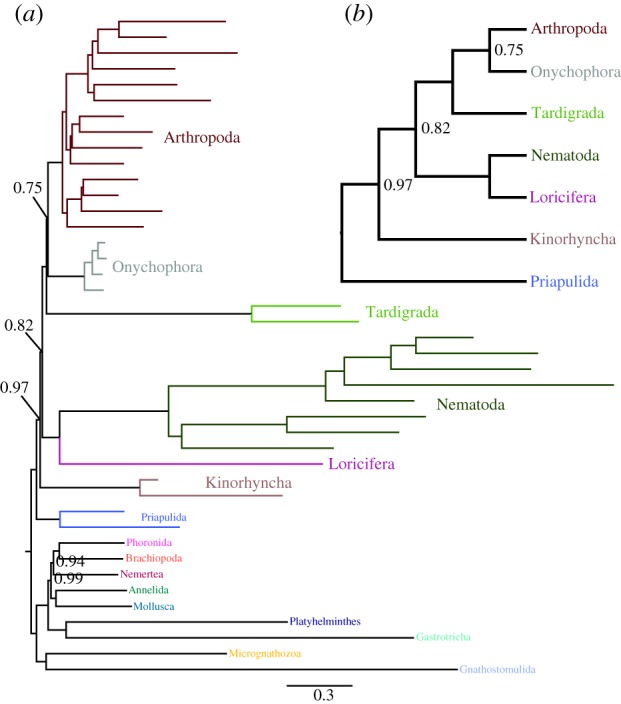
(*a*) Posterior consensus summary of CAT + GTR + *Γ*4 analysis of BMGE-trimmed Ecdysozoa-specific matrix in amino acid space, trimmed of *Nectonema* sp*.* (Nematomorpha) due to its behaviour as a rogue taxon prior to summary. (*b*) Cladogram depiction of ingroup ecdysozoan relationships within this phylogram, given to improve readability. Criteria for nodal annotation are as in figure 2. (Online version in colour.)

Due to the prevalence of rogue ecdysozoan taxa in our pan-Metazoa matrix, we constructed an Ecdysozoa-focused matrix to optimize gene sampling in these species. CAT + GTR + *Γ*4 analysis of this matrix (figure 4) gives preliminary positions for these rogue taxa: for instance, our representative of Loricifera is strongly supported here as an ingroup ecdysozoan sister group to Nematoda. As in the pan-Metazoa matrix, Priapulida falls as the sister group to the remaining members of Ecdysozoa. Kinorhyncha is not recovered as a sister group of Priapulida, instead falling out as the sister group the non-priapulan ecdysozoans (albeit with marginal support); however, we emphasize that although this matrix contains more occupied sites from Kinorhyncha than the pan-Metazoa matrix, both representatives are still only occupied in just over 4000 sites, and thus their position should be taken with caution. The sole representative of Nematomorpha in our dataset, unfortunately, still exhibits rogue taxon behaviour in CAT + GTR + *Γ*4 chains even in this matrix (and is therefore masked in the posterior summary shown in figure 4). However, in mixture-model ML analysis, it is strongly supported as a member of a clade including both Nematoda and Loricifera (electronic supplementary material, figure S18). Interestingly, although this matrix was constructed without reference to this clade, the results from its analysis under CAT + GTR + *Γ*4 recover Panarthropoda (Arthropoda + Onychophora + Tardigrada) with full support (figure 4). Curiously, however, support for Arthropoda + Onychophora within this panarthropod clade is lower than in previous studies. Furthermore, under ML, even with a profile mixture model (although less complex than the general CAT + GTR model) we fail to recover Panarthropoda, with Tardigrada strongly supported as the sister group to the Nematoda + Nematomorpha + Loricifera clade (electronic supplementary material, figure S18).

### (e) Non-bilaterian matrices (N and N′)

**Figure 5.**
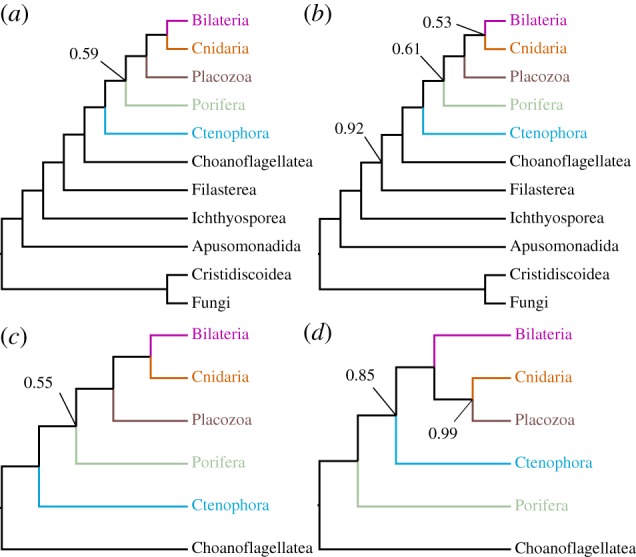
Cladograms depicting metazoan relationships outside Bilateria, summarizing relationships from CAT + GTR + *Γ*4 analysis of the non-Bilateria specific matrix, varied as follows: (*a*) amino acid matrix with all sampled opisthokont outgroups; (*b*) the same, recoded into Dayhoff-6 groups; (*c*) amino acid matrix including only Choanoflagellata as outgroups; (*d*) the same, recoded into Dayhoff-6 groups. On the matrix pruned of non-choanoflagellate outgroups, BMGE-trimming was performed after pruning, yielding a matrix of 68 337 sites, in contrast to the 61 096 sites retained when all outgroups are included prior to trimming. Trees have been arbitrarily drawn with the root between Apusomonadida and Opisthokonta. (Online version in colour.)

Recent work on metazoan relationships outside Bilateria has famously shown contrasting strong support for either Ctenophora or Porifera in the position of the sister group to the rest of Metazoa. It has been claimed that support for Ctenophora in this position even in taxonomically well-sampled datasets [9,10] is an artefact, which can be ameliorated by some combination of using adequately complex substitution models (such as CAT + GTR) [11], deleting compositionally biased, distant outgroups, and/or recoding amino acids into simpler alphabets [12,34]. To test these claims, we constructed a matrix to optimize balanced sampling of non-bilaterians, pruned of detectably compositionally biased and saturated sites (figure 1), and analysed it under CAT + GTR + *Γ*4 in both amino acid and Dayhoff-6 group codings, with or without non-choanoflagellate outgroups. Remarkably, using this matrix, we find no particular support in any of these conditions for either Porifera or Ctenophora as the sister group to the remaining Metazoa (figure 5)—unlike the strong support claimed in many recent analyses addressing this particular issue. While the combination of both Dayhoff-6 recoding and distant outgroup pruning does increase support for Porifera in this position, the posterior probability for this split is still well below most reasonable significance thresholds (figure 5*d*). Given that other matrices we have analysed under these conditions have recovered strong support for either Ctenophora (figure 2*c*) or Porifera [34] as the deepest-splitting animal phylum, it would appear that the effects of removing distant outgroups and using reduced amino acid alphabets on this problem are matrix-specific, contradicting the assertion that these approaches, in general, lessen systematic error and reveal the true phylogenetic signal in the data. It is, however, interesting to observe that in this matrix, with Dayhoff-6 recoding and removal of distant outgroups, we see strong support for Placozoa as the sister taxon to Cnidaria (figure 5*d*), mirroring the results of a separate recent study undertaken in parallel [34]. This result seems to require both factors, but is possibly more influenced by the recoding, given that support for Planulozoa (= Bilateria + Cnidaria, contra other uses [47]) is still complete in the outgroup-reduced amino acid analysis (figure 5*c*), but heavily diminished in the recoded, outgroup-unpruned analysis (figure 5*b*).

### (f) Implications and further directions for metazoan phylogenetics

Relationships within Ecdysozoa to date have not received much attention with genome-scale molecular data, perhaps owing to the rarity and/or limited nucleic acid yield in individuals of such key taxa such as Nematomorpha, Loricifera and Kinorhyncha [37,48]. Our combination of published data with new transcriptomes from representatives of all ecdysozoan taxa aspired to combat this deficiency; however, owing to our minimal taxon sampling of especially long-branched taxa such as Loricifera and Nematomorpha, combined with e.g. limited library complexity from the unamplified kinorhynch cDNA, the conclusions we can make with the data at hand are limited at best and difficult to ameliorate with new data [36] due to the duration of this study, with some analyses running longer than one year. The evidence for a monophyletic Panarthropoda found in CAT + GTR analysis of the Ecdysozoa-specific matrix (figure 4), albeit not in other more general analyses, is in close accord with other molecular evidence for the monophyly of this clade, long supported by uncontroversial morphological apomorphies such as lateral appendages [35,38]. Our results also question the notion of a monophyletic Scalidophora (Loricifera, Kinorhyncha and Priapulida), a morphologically disparate clade united only by the shared presence of innervated scalids, as the sister group to the remaining Ecdysozoa. The non-monophyly of a putative clade composed of Kinorhyncha and Priapulida in our Ecdysozoa-specific mixture model analyses (figure 4; electronic supplementary material, figure S15–S18) should be seen as at best modestly supported, and we emphasize that the matrix occupancy for our sampled kinorhynchs is no more than approximately 4% in any matrix we have analysed. By contrast, despite a still-modest matrix occupancy from the amplified cDNA library of *Armorloricus elegans*, we see strong support for a sister group relationship between Nematoda and this scalidophoran taxon (figure 4), or in trees including them, for a clade comprising Nematoda, Nematomorpha and Loricifera (electronic supplementary material, figures S15 and S18). This result recalls a previous result from ‘universal-marker' phylogenetics in which Loricifera was recovered as the sister group to Nematomorpha, this clade itself sister to Nematoda [49]. That we report genome-scale evidence for a very similar position might, therefore, bolster the suggested homologies between larval and adult loriciferans and the nematomorph gordiid larva. However, we emphasize that this result should be seen as provisional, pending a synoptic analysis of Ecdysozoa with good gene and taxon sampling, especially within Nematomorpha and all members of Scalidophora.

Relationships within the spiralian subclade Lophotrochozoa, despite our excellent gene and taxon sampling of this clade, have continued to be volatile in our analyses and contrast with the recent study by Marlétaz *et al*. [18]. One consistent element is the support (figures 2*a* and 3) for a monophyletic Lophophorata, which validates the homology of the lophophore and associated structures in these taxa. The specific topology we recover within Lophophorata, with Phoronida and Byrozoa being well-supported sister taxa, similar to other recent phylogenies that control systematic error [26,50], hearkens to earlier morphological hypotheses of lophophorate phylogeny which homologized the epistome of phylactolaemate bryozoans with that of phoronids [51], for example, and contradicts the assertion principally founded by rRNA phylogenetics that Phoronida represents a subtaxon of Brachiopoda, sister group to Inarticulata [52–54]. In a recoded analysis (figure 2*c*), we have also recovered, for the first time to our knowledge in a molecular phylogeny, strong support for this monophyletic lophophorate clade as the sister group to Entoprocta/Cycliophora. The existence of such a clade, which might imply that the long-branched Entoprocta/Cycliophora are being driven in amino acid analyses outside Lophotrochozoa towards the platyzoan taxa, certainly requires further validation, especially in the context of the recent analysis that, when excluding Cycliophora, place Entoprocta with Mollusca [18]. If corroborated by other analyses, however, our clade may resurrect the core aspect of the Polyzoa hypothesis: that Bryozoa and Entoprocta descend from a common ancestor with asexual budding and/or coloniality [1]. Indeed, considering that the funnel replacement mechanism of cycliophorans is homologous to the process of budding and that many phoronid species also reproduce asexually by transverse fission or budding [55], this would make modern Brachiopoda the only taxon of this sessile clade with U-shaped guts to lack asexual reproduction. However, comparisons between modern taxa may be misleading without reference to the fossil record, which is rich for this lineage. Perhaps most worthy of consideration in light of this topology is *Cotyledion*, interpreted as a macrofaunal, solitary entoproct whose calyx was armed by mineralized sclerites, implying that the minute size and pseudocoelomate nature of modern entoprocts, as well as the absence of sclerites, may be derived features [56]. A sister group relationship between Entoprocta + Cycliophora and Lophophorata would be consistent with the possibility that not only the sessile habit and U-shaped gut of these taxa are homologous, but also that the sclerites of *Cotyledion* and presumably other stem entoprocts might be homologous to those of other fossil lophophorates (e.g. tommotiids [54]), or indeed more deeply to those of other ‘small shelly fossil' taxa assigned to Lophotrochozoa (e.g. halkieriids [57], possibly chancelloriids [58]). Given the putative spiral cleavage [59] and putative trochophore larva [60] of some species of Entoprocta, this would further bolster the notion that Lophophorata have lost these developmental modes inherited from at least the ancestor of Lophotrochozoa, a scenario which is also becoming increasingly clear on developmental grounds alone [61]. The fact that *Cotyledion* to all appearances is a solitary animal would indicate that either the budding of modern Bryozoa (and possibly Phoronida) and Entoprocta + Cycliophora are convergent reproductive modes, or that modern Brachiopoda and possibly the stem leading to *Cotyledion* have convergently lost this mode of reproduction. Further knowledge and phylogenetic placement of problematic fossils which show evidence of clonal reproduction, which have also been attributed to total group Lophophorata, may help further clarify this question [62,63].

The recognition that the mostly meiofaunal, acoelomate members of Spiralia, namely Platyhelminthes (which despite its many macrofaunal lineages, was ancestrally surely meiofaunal [64,65]), Gastrotricha and Gnathifera, comprise two separate, deeply splitting lineages within this clade (Rouphozoa and Gnathifera), had been previously used to argue that the spiralian ancestor may, perhaps inheriting traits from an earlier bilaterian ancestor [7], plausibly have been itself a relatively simple, microscopic, acoelomate worm [39,40]. Our analyses complicate this picture. Simply the recognition that Chaetognatha, a group of coeolomate macroscopic worms with a complete gut, may form a sister group to Gnathifera, makes it more difficult to reconstruct the spiralian ancestor as an organism superficially similar to a modern platyhelminth or gnathostomulid. The placement of Platyhelminthes as sister group to Nemertea, and of Gastrotricha as sister group to Lophophorata within the traditional coelomate spiralians by Marlétaz *et al*. [18]—who, however, chiefly arrive at this result through taxon deletion as a means of mitigating compositional bias—would challenge this hypothesis even further. Indeed, simply considering only our own results, it may be worthwhile to question even the monophyly of Lophotrochozoa (exclusive of Platyhelminthes and Gastrotricha), another foundation of the ‘platyzoan paraphyly' hypothesis for which support is lacking in both of the recoded analyses including spiralian taxa that we analyse here (figure 2*c*; electronic supplementary material, figure s13).

## 4. Conclusion

Resolving the most ancient relationships among animals with large-scale molecular datasets continues to present several frustrating paradoxes, often not recognized in recent publications, which tend to claim resolution with strong support for clades that remain in conflict in this thorough study. As the number of taxa for which sequence data are available grows, at least partly as a result of the prevalence of lineage-specific genes, the number of large orthologues available to study deep relationships with balanced matrix occupancy diminishes. Researchers may feel the need to even further reduce the number of sites in a matrix as the number of taxa increases, since only the most complicated, computationally demanding models yield reasonable results on datasets that span many billions of years of collective evolutionary divergence. Even with tens of thousands of well-aligned sites, stringently validated orthology, and the most flexible, descriptive site-heterogeneous models available today, different gene sets can give full support to conflicting phylogenies. Indeed, even examination of a single matrix under a single model—but using different taxon sets, or masking all but a subset of the recorded substitutions—can yield strong conflicts.

We view such conflicts as reasons for optimism—in true Socratic fashion, they let us know what we do not yet know. Compared to the situation a decade ago, it is now a relatively small list. We consider the chief outstanding goals to be:

— Verifying the status of Porifera or Ctenophora as the sister group of the remaining metazoans.
— Defining Chaetognatha as a member of Gnathifera or as the sister group of this clade.
— Clarifying which taxon is the sister group of Cnidaria (an issue recently complicated by both conventional molecular phylogenetic analysis [34], analysis of gene family gain and loss [16], and new fossil evidence [66]).
— Continuing to interrogate the position of Xenoacoelomorpha (not addressed here).
— Testing the monophyly of Scalidophora, Panarthropoda and Lophotrochozoa.
— Within the latter, precisely defining the relationships among a monophyletic Lophophorata, Entoprocta + Cycliophora, and the remaining three trochozoan phyla (Annelida, Mollusca and Nemertea).

Defining such fixed-scope problems provides a powerful approach moving forward: clade-specific matrices constructed to test a minimal number of relationships among taxa already demonstrated to be monophyletic allows a much larger number of genes and sites to be examined. Matrices made within such gene sets also are less likely to violate—or perhaps simply less strongly violate—the stationarity assumptions still made by almost all practical phylogenetic inference software: that a single frequency vector can describe composition across the tree [67,68]; that rates of evolution at given sites do not differ among taxa [69]; and that a single substitution matrix accurately describes evolution at a single site among distantly related clades [70]. We hypothesize that such model violations are likely to eventually explain many of the conflicts we and others have seen in metazoan molecular phylogenies. In the near term, we see hope for controlling such violations by limiting the summed patristic distance of a matrix to the minimum required to test relationships with good taxon sampling of the clades in question, and by using sensitive statistical tests to detect and remove sites and genes that show evidence of non-stationarity. Using reduced amino acid alphabets may also mask some forms of non-stationary substitution without removing the site outright, and such reduced state matrices have the advantage of being computationally much simpler to model in useful timeframes. However, the theoretical properties of different recoding schemes remain barely understood [71].

The call to punctiliously discard data which violate the stationarity assumptions used to infer phylogenies will be much easier to meet when highly contiguous, well-annotated genomes—now routinely and economically generated with third generation sequencing [72,73]—are used exclusively; it may be the beginning of the end of the days of using incomplete transcriptome assemblies as an interim approximation to genomes, as done here. Such datasets will also make it much more straightforward to detect genomic changes that bear phylogenetic signal besides those observable in multiple sequence alignments [74,75]. In the long term, however, perhaps the best hope for resolving persistent phylogenetic conflicts in Metazoa and elsewhere will come not from the generation of more data [29], but from analysis of such data with practical, computationally scalable [76] software that flexibly describes heterogeneity in sequence evolution not only among sites, but also through time.

## Supplementary Material

Supplemental Methods, Figures, and Table

## Data accessibility

All newly sequenced libraries have been given NCBI accessions reported in electronic supplementary material, table S1. Predicted peptides from each species, primary OrthoFinder outputs, the 5511 UPhO groups used for gene selection, all matrices provided as phylip files, and all phylogenetic trees and associated files, are available from the Dryad Digital Repository: https://doi.org/10.5061/dryad.293kp3d [77].
