# Supplementary material for: Revisiting metazoan phylogeny with genomic sampling of all phyla
Source: Proc Biol Sci. 2019 Jul 10;286(1906):20190831. doi: 10.1098/rspb.2019.0831 (PMC6650721; doi:10.1098/rspb.2019.0831)
Supplement: Supplemental Methods, Figures, and Table [file rspb20190831supp1.pdf]

Supplemental materials and methods

Sample collection, preservation, and RNA extraction

Specimens were preserved during field collections in RNA*later* (Ambion) according to the manufacturer's recommendations, flash frozen at -80 C, or directly placed into 0.5 mL TRIzol for RNA extraction, and total RNA was extracted with established protocols, homogenizing LN2-frozen samples directly within 0.5-1.0 mL TRIzol (Riesgo et al., 2012b, 2012a). Tissues with RNA yields expected to be within the sensitivity range of fragment analysis were routinely QC'd by Agilent Bioanalyzer 2100, using RNA 6000 Pico or Nano kits (Agilent, Inc, cat. #s 5067-1511 and 5067-1513). Polyadenylated mRNA was enriched using the Dynabeads™ mRNA Purification Kit (ThermoFisher, Inc., cat. # 61006), eluting in up to 10 uL of 10 mM Tris-HCl, pH 7.5.

Transcriptome library generation

Except where specified, mRNA was converted into fragmented cDNA libraries and indexed for Illumina sequencing using an automated library construction robot, the Apollo 324 (originally manufactured by IntegenX Inc., then Wafergen, Inc., and currently by TaKaRa Bio, Inc). We used the PrepX RNA-seq kit (TaKaRa, cat. # 640096), which RNAse III-fragments mRNA and performs single-stranded adapter ligation prior to reverse transcription, to prepare up to 8 libraries at once, and used manufacturer-provided indexing primers to assign sample barcodes. Libraries were QC'd using the KAPA Library Quantification kit (KAPA Biosciences, Inc. KK4824, now Roche cat. # 07960140001), and the Agilent 2100 High Sensitivity DNA kit (Agilent, Inc. cat. # 5067-4626). Pools were prepared with plexity 6-12, and sequenced as paired-end 150 bp or 250 bp reads on Illumina HiSeq 2500 instruments, typically as rapid runs.

Whole genome and transcriptome amplified samples

For the gnathostomulid *Tenuignathia rikerae*, we attempted a genome assembly from DNA amplified from a single individual by multiple displacement amplification. In brief, a single RNAlater preserved specimen was rinsed in distilled water, and kept in a lens of ~2 µL, to which alkaline lysis buffer from the Qiagen REPLI-g Single-Cell Kit (Qiagen, Inc., cat. # 150343) was directly added, and DNA amplified as per manufacturer's recommendations with the single-cell protocol, incubating at 30 C for 8 hours. MDA product was QC'd and confirmed to be HMW (70 kbp+) on an Agilent Genomic DNA TapeStation 2200 kit (Agilent, Inc. cat. # 5067-5365). One aliquot was taken and fragmented to ~350 bp using a Covaris S220 focused ultrasonicator and prepared as an Illumina short-insert genomic DNA library using a previously described protocol for end-repair/A-tailing and ligation of Y-shaped adapters (Neiman et al., 2012), followed by limited library amplification with indexing primers and KAPA HiFi polymerase (KK2103). 1 µg of MDA product was processed into a mate-pair library using the Illumina Nextera Mate-Pair Library Preparation Kit (Illumina, Inc. cat. # FC-132-1001), following the manufacturer's gel-free protocol, and amplifying the final Illumina library with indexing oligos and the KAPA HiFi polymerase. Adapter sequences were trimmed from the mate-pair library using NextClip (Leggett et al., 2014) using default parameters, and from the short-insert library using Trimmomatic as described above. An initial assembly using SPAdes v3.2.0 was created running in single-cell mode; however, the assembly failed to finish at the k-mer size step of 55. We therefore took the error-corrected reads output by SPAdes, and used these as inputs for

assembly with IDBA_UD v1.1.1 (Peng et al., 2012). The resulting assembly remained highly fragmented (1,691,903 scaffolds of which 1,140,751 were <200 bp, with a 496.07 Mbp span, a scaffold N50 of 360 bp, and GC% of 32.25). Nonetheless, we conducted an ab initio gene prediction effort on the resultant assembly, using Augustus v3.2.2, and though still highly incomplete (e.g. with only 37.29% and 61.39% of complete and complete+partial BUSCO v3 eukaryotic core genes, respectively), these peptides were still judged to be a sufficiently large sample to be useful in the context of phylogenetic inference.

For a number of samples (marked by a * in Supplemental Table S1), we chose to utilize an alternative approach to transcriptome library preparation, undertaking blunt-end circularization of double-stranded cDNA and phi29-mediated rolling-circle amplification, the so-called PMA method (Pan et al., 2013), reverse-transcribing, forming second-strand cDNA, and circularizing following the published description, but leveraging the Qiagen REPLI-g Single-Cell kits protocol for amplifying purified DNA. These amplified cDNAs were then fragmented to ~350 bp inserts via Covaris S220 sonication, and prepared into short-insert Illumina libraries as described above.

Transcriptome assembly and peptide prediction

Demultiplexed FASTQ files were trimmed of adapters and low-quality sequences using Trimmomatic v0.32 (Bolger et al., 2014) and assembled in Trinity (Haas et al., 2013). Explicit efforts to control cross-contamination from index swapping were not made during the period of dataset curation; however, we point out that transcriptome libraries prepared in this laboratory have shown exceptionally low levels of cross contamination in secondary analysis (Ballenghien et al., 2017; Simion et al., 2018). Putative coding sequences were predicted using TransDecoder, and the longest ORFs per Trinity subcomponents were extracted as described elsewhere (Laumer et al., 2015), serving as proxies for the most complete isoform per unigene.

Orthologue assignment

Representative proteomes from derived from Trinity transcriptome assemblies or annotated genome assemblies from 202 species representing all animal phyla and unikont outgroups were brought together (Supplemental Table S1) for an all-by-all blastp task, driven by a series of highly parallel job arrays on the EBI high-performance compute cluster and the Harvard Odyssey 2.0 research computing cluster. Results were formatted as tsv files described in the documentation for use of precomputed blast results, and the OrthoFinder v0.4 algorithm (Emms and Kelly, 2015) was used to group peptide sequences into 1,648,840 top-level gene families (most very small in sequence occupancy), calling with the '--only-groups' flag to stop the algorithm prior to orthogroup alignment and gene tree construction, which was conducted manually.

From these top-level orthogroups, a highly reduced set of 7,437 groups were selected, which each had at least 10 separate phyla (or higher outgroup clades, as specified by the first four letters of each taxon abbreviation in Supplemental Table S1). The PASTA algorithm (Mirarab et al., 2015) run with default parameters was used to iteratively produce gene trees (calling the FastTree2 algorithm) and gapped multiple sequence alignments. From the successfully completed alignments, we ran the ZORRO algorithm to mask all columns with confidence score <= 0.1, and removed all sequences from each orthogroup which were composed of more than 50% gaps, with the intention to select against highly incompletely assembled transcripts. From the remaining orthogroup set, we selected 5,645

groups which contained more than 50 sequences and 50 columns. At this stage, we reconstituted the orthogroups using the original, untrimmed sequences, and used the L-INS-I algorithm of the MAFFT aligner (Katoh and Standley, 2013) to produce new multiple sequence alignment. These we again masked with ZORRO, this time retaining all columns with a confidence score of > 0.5, and from these masked alignments produced gene trees with RAxML, called with the PROTCATLGF model and producing support values with 50 rapid bootstrap replicates. These gene trees and their originating alignments were further curated with python scripts from the Phylogenomic Dataset Construction (PDC) pipeline (Yang and Smith, 2014), calling the following three scripts in succession within the working directory as such:

'python trim_tips.py . .tre 5 10' (removing all tree tips that were >5x longer than their sister, as well as all tips that were over branch length 10)

'python mask_tips_by_taxonID_transcripts.py . . y' (masking all sequences from the same species that formed a monophyletic or paraphyletic group, retaining the one with the longest proportion of aligned residues)

'python cut_long_internal_branches.py . tt.mm 2 30 cut_2'. (cutting all gene families into smaller groups of size at least 30 if an edge within the gene tree had branch length of 2 or longer)

From this process survived 5,578 gene trees. These were then parsed to extract unambiguously orthologous groups, using the criterion implemented in the Unrooted Phylogenetic Orthology (UPhO) algorithm (Ballesteros and Hormiga, 2016), yielding 5,511 final orthogroups comprising 195 taxa.

Matrix construction

To construct the pan-metazoa matrix, we selected a set of 422 alignments from the initial set of 1,034 that were seen to have at least 100 sequences, by calling the MARE algorithm, initially concatenating the 1,034 with phyutility's '-concat' option (Smith and Dunn, 2008), converting the resulting NEXUS file into a FASTA in BioPython and extracting the partition list with vi regular expressions, and running MARE v0.12 with the options '-t 100' '-d 0.5' to force retention of all taxa and select for a matrix judged to be of adequate (initially at least 100,000 residues) size. Inspection of this matrix and initial trees, and a contemporaneous publication reporting increased compositional artifacts from including outgroups more distant than Choanoflagellata (Pisani et al., 2015), led us to remove 28 taxa judged to be redundant representatives of their lineage and/or too sparsely represented in the matrix, plus all non-choanoflagellate outgroups. The initial 422-gene matrix of 106,186 residues was split into 10 nearly equally sized parts using BioPython, as a rough parallelization of this job which ran very slowly as a serial process on the intact matrix, and BMGE v1.12 was run with options '-g 0', '-h 0.5', '-m BLOSUM30', and '-s FAST' to retain all gapped sites, but trim those that failed its approximate non-stationarity test, and those judged to be entropic relative to the BLOSUM30 matrix (Criscuolo and Gribaldo, 2010). These parts were then re-joined using BioPython to retain the final matrix. As reported in the main text, this process was performed both before and after the deletion of the 28 taxa, with different results on the proportion of sites retained.

For our subclade-specific matrices, we considered from among the set of 5,511 UPhO groups a smaller set of 3,824 groups with at least 50 sequences, and created initial sets using the arbitrary taxon inclusion criteria specified within the main text. MARE was used as described above to further

reduce these sets to matrix sizes deemed appropriate for CAT+GTR analysis (approximately 50-100k sites), tuning the '-d' parameter to effect this result, and the resultant matrix was then treated with BMGE prior to phylogenetic analysis, again as called above.

Phylogenetic analyses

In general, CAT+GTR+Γ4 analyses were conducted using the PhyloBayes-MPI v1.6j release (Lartillot et al., 2013), starting each chain without specifying an initial tree, specifying the model with '-cat' '-gtr', removing constant sites with the '-dc' option, and running each generally with 10 to 20 MPI processes. Dayhoff-6 recoded analyses were conducted with the same default parameters, but in Phylobayes v4.1c serial (Lartillot et al., 2009), specifying '-dayhoff6' to initiate recoding. To trim suspected rogue taxa, we first used the RogueNaRok algorithm (Aberer et al., 2013) with default parameters to inspect .treelist files, and removed the species thus identified using the '-prune' option of phyutility, prior to a new summary.

IQ-tree (Nguyen et al., 2015) was run using specified versions, typically as a multithreaded process using '-nt 10' or AUTO, and selecting '-bb 1000' and '-bnni' to run 1,000 UFboot2 replicates corrected with nearest-neighbor interchanges. Models were specified with the '-m' parameter as described in figure captions; in general, whenever the PMSF model was applied (Wang et al., 2018), it was used to approximate the C60+LG+FO+R4 profile mixture model, and was called using an initial guide tree inferred with '-m LG4X+FO+R4'.

References

Aberer, A.J., Krompass, D., Stamatakis, A., 2013. Pruning Rogue Taxa Improves Phylogenetic Accuracy: An Efficient Algorithm and Webservice. Syst. Biol. 62, 162–166. https://doi.org/10.1093/sysbio/sys078

Ballenghien, M., Faivre, N., Galtier, N., 2017. Patterns of cross-contamination in a multispecies population genomic project: detection, quantification, impact, and solutions. BMC Biol. 15, 25. https://doi.org/10.1186/s12915-017-0366-6

Ballesteros, J.A., Hormiga, G., 2016. A new orthology assessment method for phylogenomic data: Unrooted Phylogenetic Orthology. Mol. Biol. Evol. 33, 2117–2134. https://doi.org/10.1093/molbev/msw069

Bolger, A.M., Lohse, M., Usadel, B., 2014. Trimmomatic: a flexible trimmer for Illumina sequence data. Bioinformatics 30, 2114–2120. https://doi.org/10.1093/bioinformatics/btu170

Criscuolo, A., Gribaldo, S., 2010. BMGE (Block Mapping and Gathering with Entropy): a new software for selection of phylogenetic informative regions from multiple sequence alignments. BMC Evol. Biol. 10, 210. https://doi.org/10.1186/1471-2148-10-210

Emms, D.M., Kelly, S., 2015. OrthoFinder: solving fundamental biases in whole genome comparisons dramatically improves orthogroup inference accuracy. Genome Biol. 16, 157. https://doi.org/10.1186/s13059-015-0721-2

Haas, B.J., Papanicolaou, A., Yassour, M., Grabherr, M., Blood, P.D., Bowden, J., Couger, M.B., Eccles, D., Li, B., Lieber, M., MacManes, M.D., Ott, M., Orvis, J., Pochet, N., Strozzi, F., Weeks, N., Westerman, R., William, T., Dewey, C.N., Henschel, R., LeDuc, R.D., Friedman, N., Regev, A., 2013. *De novo* transcript sequence reconstruction from RNA-seq using the Trinity platform for reference generation and analysis. Nat. Protoc. 8, 1494–1512. https://doi.org/10.1038/nprot.2013.084

Katoh, K., Standley, D.M., 2013. MAFFT Multiple Sequence Alignment Software Version 7: Improvements in Performance and Usability. Mol. Biol. Evol. 30, 772. https://doi.org/10.1093/molbev/mst010

Lartillot, N., Lepage, T., Blanquart, S., 2009. PhyloBayes 3: a Bayesian software package for phylogenetic reconstruction and molecular dating. Bioinformatics 25, 2286–2288. https://doi.org/10.1093/bioinformatics/btp368

Lartillot, N., Rodrigue, N., Stubbs, D., Richer, J., 2013. PhyloBayes MPI: phylogenetic reconstruction with infinite mixtures of profiles in a parallel environment. Syst. Biol. 62, 611–615. https://doi.org/10.1093/sysbio/syt022

Laumer, C.E., Hejnol, A., Giribet, G., 2015. Nuclear genomic signals of the 'microturbellarian' roots of platyhelminth evolutionary innovation. eLife 4, e05503. https://doi.org/10.7554/eLife.05503

Leggett, R.M., Clavijo, B.J., Clissold, L., Clark, M.D., Caccamo, M., 2014. NextClip: an analysis and read preparation tool for Nextera Long Mate Pair libraries. Bioinformatics 30, 566–568. https://doi.org/10.1093/bioinformatics/btt702

Mirarab, S., Nguyen, N., Guo, S., Wang, L.-S., Kim, J., Warnow, T., 2015. PASTA: Ultra-Large Multiple Sequence Alignment for Nucleotide and Amino-Acid Sequences. J. Comput. Biol. 22, 377–386. https://doi.org/10.1089/cmb.2014.0156

Neiman, M., Sundling, S., Grönberg, H., Hall, P., Czene, K., Lindberg, J., Klevebring, D., 2012. Library Preparation and Multiplex Capture for Massive Parallel Sequencing Applications Made Efficient and Easy. PLOS ONE 7, e48616. https://doi.org/10.1371/journal.pone.0048616

Nguyen, L.-T., Schmidt, H.A., von Haeseler, A., Minh, B.Q., 2015. IQ-TREE: A Fast and Effective Stochastic Algorithm for Estimating Maximum-Likelihood Phylogenies. Mol. Biol. Evol. 32, 268–274. https://doi.org/10.1093/molbev/msu300

Pan, X., Durrett, R.E., Zhu, H., Tanaka, Y., Li, Y., Zi, X., Marjani, S.L., Euskirchen, G., Ma, C., LaMotte, R.H., Park, I.-H., Snyder, M.P., Mason, C.E., Weissman, S.M., 2013. Two methods for full-length RNA sequencing for low quantities of cells and single cells. Proc. Natl. Acad. Sci. U. S. A. 110, 594–599. https://doi.org/10.1073/pnas.1217322109

Peng, Y., Leung, H.C.M., Yiu, S.M., Chin, F.Y.L., 2012. IDBA-UD: a de novo assembler for single-cell and metagenomic sequencing data with highly uneven depth. Bioinforma. Oxf. Engl. 28, 1420–1428. https://doi.org/10.1093/bioinformatics/bts174

Pisani, D., Pett, W., Dohrmann, M., Feuda, R., Rota-Stabelli, O., Philippe, H., Lartillot, N., Wörheide, G., 2015. Genomic data do not support comb jellies as the sister group to all other animals. Proc. Natl. Acad. Sci. 112, 15402–15407. https://doi.org/10.1073/pnas.1518127112

Riesgo, A., Andrade, S.C.S., Sharma, P.P., Novo, M., Pérez-Porro, A.R., Vahtera, V., González, V.L., Kawauchi, G.Y., Giribet, G., 2012a. Comparative description of ten transcriptomes of newly sequenced invertebrates and efficiency estimation of genomic sampling in non-model taxa. Front. Zool. 9, 33. https://doi.org/10.1186/1742-9994-9-33

Riesgo, A., Pérez-Porro, A.R., Carmona, S., Leys, S.P., Giribet, G., 2012b. Optimization of preservation and storage time of sponge tissues to obtain quality mRNA for next-generation sequencing. Mol. Ecol. Resour. 12, 312–322. https://doi.org/10.1111/j.1755-0998.2011.03097.x

Simion, P., Belkhir, K., François, C., Veyssier, J., Rink, J.C., Manuel, M., Philippe, H., Telford, M.J., 2018. A software tool "CroCo" detects pervasive cross-species contamination in next generation sequencing data. BMC Biol. 16, 28. https://doi.org/10.1186/s12915-018-0486-7

Smith, S.A., Dunn, C.W., 2008. Phyutility: a phyloinformatics tool for trees, alignments and molecular data. Bioinformatics 24, 715–716. https://doi.org/10.1093/bioinformatics/btm619

Wang, H.-C., Minh, B.Q., Susko, E., Roger, A.J., 2018. Modeling Site Heterogeneity with Posterior Mean Site Frequency Profiles Accelerates Accurate Phylogenomic Estimation. Syst. Biol. 67, 216–235. https://doi.org/10.1093/sysbio/syx068

Yang, Y., Smith, S.A., 2014. Orthology inference in nonmodel organisms using transcriptomes and low-coverage genomes: improving accuracy and matrix occupancy for phylogenomics. Mol. Biol. Evol. 31, 3081–3092. https://doi.org/10.1093/molbev/msu245

Supplementary Figure and Table Captions

Figure S1 – Maximum Likelihood cladogram from concatenated analysis of all 3,824 trimmed orthologue alignments with greater than 50 species (201 taxa, 1,263,500 sites), inferred in IQ-tree v 1.6.3 under the LG4X+R4+FO model, with 1,000 UFboot2 replicates corrected with NNI.

Figure S2 – Maximum Likelihood cladogram from the pan-Metazoa matrix with no taxon or site trimming (195 taxa, 106,186 sites), inferred in IQ-tree v1.6.2 under the PMSF approximation of the C60+LG+FO+R4 model, with 1,000 UFboot2 replicates corrected with NNI. In this and all other PMSF-profile derived trees, an initial guide tree was inferred under the LG4X mixture model.

Figure S3 – Cladogram from posterior consensus summary of CAT+GTR+Γ4 analysis of post-BMGE taxon-pruned, 43,011 site pan-Metazoa matrix, with rogue taxa masked prior to summary. Two chains were used to form this consensus, sampling every 10 generations, discarding the first 2,000 as burn-in, yielding a maxdiff of 0.309244.

Figure S4 – Cladogram from posterior consensus summary of CAT+GTR+Γ4 analysis of post-BMGE taxon-pruned, 43,011 site pan-Metazoa matrix, with no rogue taxon masking prior to summary. Two chains were used to form this consensus, sampling every 10 generations, discarding the first 2,000 as burn-in, yielding a maxdiff of 0.309244.

Figure S5 – Cladogram from Maximum Likelihood analysis of the post-BMGE taxon-pruned, 43,011 site pan-Metazoa matrix, inferred in IQ-tree v1.6.2 under the PMSF approximation of the C60+LG+FO+R4 model, with 1000 UFboot2 replicates corrected with NNI.

Figure S6 – Cladogram from posterior consensus summary of CAT+GTR+Γ4 analysis of pan-Metazoa matrix M, but with no rogue taxon masking prior to summary. Three chains were used to form this consensus, sampling every 10 generations and discarding the first 14,000 as burn-in, yielding a maxdiff of 0.21288.

Figure S7 - Cladogram from posterior consensus summary of CAT+GTR+Γ4 analysis of pan-Metazoa matrix M, with rogue taxon masking prior to summary, and full pp and taxon labels. Three chains were used to form this consensus, sampling every 10 generations and discarding the first 14,000 as burn-in, yielding a maxdiff of 0.21288.

Figure S8 – Cladogram from posterior consensus summary of CAT+GTR+Γ4 analysis of pan-Metazoa matrix M, recoded into in Dayhoff-6 groups, the same as presented in Figure 2b, but with no rogue taxon masking prior to summary. Three chains were used to form this consensus, sampling every 10 generations and discarding the first 2,100 as burn-in, yielding a maxdiff of 0.543937.

Figure S9 – Cladogram from posterior consensus summary of CAT+GTR+Γ4 analysis of pan-Metazoa matrix M, recoded into in Dayhoff-6 groups, the same as presented in Figure 2b, with rogue taxon masking prior to summary. Three chains were used to

form this consensus, sampling every 10 generations and discarding the first 2,100 as burn-in, yielding a maxdiff of 0.228175.

Figure S10 -- Maximum Likelihood cladogram derived from pan-Metazoa matrix M, inferred in IQ-tree v1.6.2 under the PMSF approximation of the C60+LG+FO+R4 model, with 1000 UFboot2 replicates corrected with NNI.

Figure S11 -- Cladogram from posterior consensus summary of CAT+GTR+Γ4 analysis of "Spiralia" matrix S, the same as presented in Figure 3, but with no rogue taxon masking prior to summary. Two chains were used to form this consensus, sampling every 10 generations and discarding the first 6,000 as burn-in, yielding a maxdiff of 0.146158.

Figure S12 -- Cladogram from posterior consensus summary of CAT+GTR+Γ4 analysis of "Spiralia" matrix S, the same as presented in Figure 3, with rogue taxon masking prior to summary, and full pp and taxon labels. Two chains were used to form this consensus, sampling every 10 generations and discarding the first 6,000 as burn-in, yielding a maxdiff of 0.140087.

Figure S13 -- Cladogram from posterior consensus summary of CAT+GTR+Γ4 analysis of "Spiralia" matrix S, recoded into Dayhoff-6 groups, with rogue taxon masking prior to summary. Two chains were used to form this consensus, sampling every 10 generations and discarding the first 2,000 as burn-in, yielding a maxdiff of 0.0884638.

Figure S14 -- Maximum Likelihood cladogram derived from "Spiralia" matrix S, inferred in IQ-tree v1.6.2 under the PMSF approximation of the C60+LG+FO+R4 model, with 1000 UFboot2 replicates corrected with NNI. The position of the root is arbitrarily drawn between the clade of Syndermata+Micrognathozoa+Chaetognatha and the remaining taxa.

Figure S15 -- Cladogram from posterior consensus summary of CAT+GTR+Γ4 analysis of "Ecdysozoa" matrix E, the same as presented in Figure 4, but with no rogue taxon masking prior to summary. Two chains were used to form this consensus, sampling every 10 generations and discarding the first 2,000 as burn-in, yielding a maxdiff of 0.552004.

Figure S16 -- Cladogram from posterior consensus summary of CAT+GTR+Γ4 analysis of "Ecdysozoa" matrix E, the same as presented in Figure 4, with rogue taxon masking prior to summary, and full pp and taxon labels. Two chains were used to form this consensus, sampling every 10 generations and discarding the first 2,000 as burn-in, yielding a maxdiff of 0.0839663.

Figure S17 -- Cladogram from posterior consensus summary of CAT+GTR+Γ4 analysis of "Ecdysozoa" matrix E, recoded into Dayhoff-6 groups, with rogue taxon masking prior to summary. Two chains were used to form this consensus, sampling every 10 generations and discarding the first 2,000 as burn-in, yielding a maxdiff of 0.0884638.

Figure S18 -- Maximum Likelihood cladogram derived from "Ecdysozoa" matrix E, inferred in IQ-tree v1.6.2 under the PMSF approximation of the C60+LG+FO+R4 model, with 1000 UFboot2 replicates corrected with NNI.

Figure S19 -- Cladogram from posterior consensus summary of CAT+GTR+Γ4 amino acid analysis of "Non-Bilateria" matrix N, the same as presented in Figure 5A. Two chains were used to form this consensus, sampling every 10 generations and discarding the first 5,000 as burn-in, yielding a maxdiff of 0.078028.

Figure S20 -- Cladogram from posterior consensus summary of CAT+GTR+Γ4 Dayhoff-6 recoded analysis of "Non-Bilateria" matrix N, the same as presented in Figure 5B. Two chains were used to form this consensus, sampling every 10 generations and discarding the first 5,000 as burn-in, yielding a maxdiff of 0.239156.

Figure S21 -- Cladogram from posterior consensus summary of CAT+GTR+Γ4 amino acid analysis of "Non-Bilateria" matrix N', the same as presented in Figure 5C. Two chains were used to form this consensus, sampling every 10 generations and discarding the first 5,000 as burn-in, yielding a maxdiff of 0.0944149.

Figure S22 -- Cladogram from posterior consensus summary of CAT+GTR+Γ4 Dayhoff-6 recoded analysis of "Non-Bilateria" matrix N', the same as presented in Figure 5D. Two chains were used to form this consensus, sampling every 10 generations and discarding the first 2,000 as burn-in, yielding a maxdiff of 0.06295.

Figure S23 – Cladogram from ML analysis of “Non-Bilateria” matrix N, inferred in IQ-tree v1.6.2 under the PMSF approximation of the C60+LG+FO+R4 model, with 1000 UFboot2 replicates corrected with NNI.

Figure S24 – Cladogram from ML analysis of “Non-Bilateria” matrix N’, inferred in IQ-tree v1.6.2 under the PMSF approximation of the C60+LG+FO+R4 model, with 1000 UFboot2 replicates corrected with NNI.

Table S1 – Mapping between full species name, abbreviated names used in Figures S1-S24, assembly type, and SRA/Bioproject numbers/genome repository for raw data source. Taxa annotated with * represent rogue taxa masked in Matrix M, those annotated with † were deleted during the construction of Matrix M, and those annotated with ‡ were taxa included in the orthology analysis, but which were not included among the alignments with minimum 100 spp.

S1

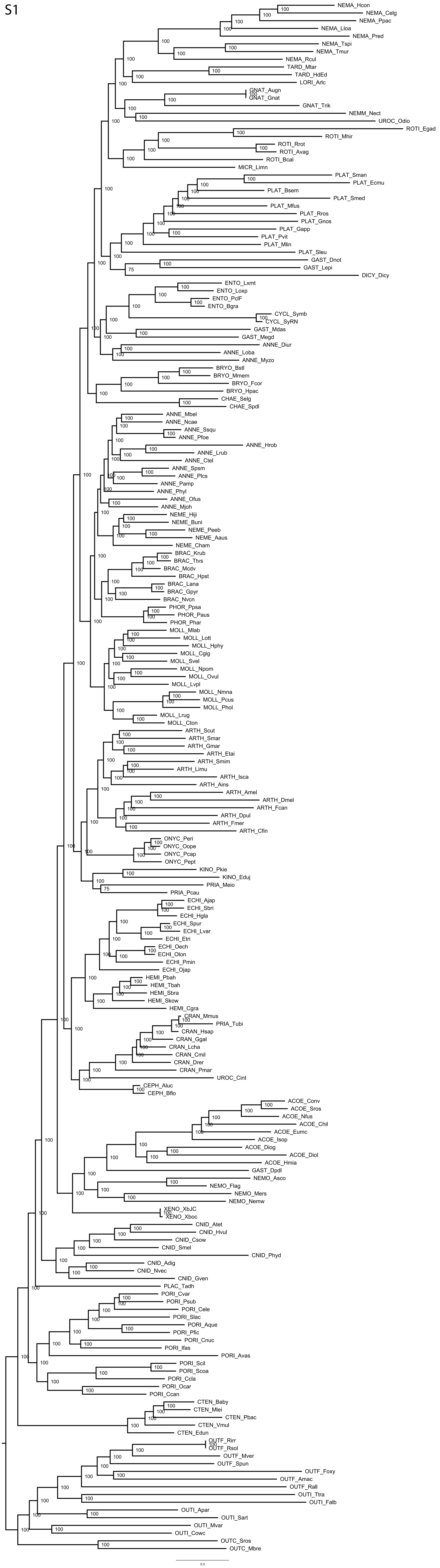

NEMA_Hcon
NEMA_Celg
NEMA_Ppac
NEMA_Lloa
NEMA_Pred
NEMA_Tspi
NEMA_Tmur
NEMA_Rcul
TARD_Mtar
TARD_HdEd
LORI_Arlc
GNAT_Augn
GNAT_Gnat
GNAT_Trik
NEMM_Nect
UROC_Odio
ROTI_Egad
ROTI_Mhir
ROTI_Rrot
ROTI_Avag
ROTI_Bcal
MICR_Limn
PLAT_Sman
PLAT_Ecmu
PLAT_Bsem
PLAT_Smed
PLAT_Mfus
PLAT_Rros
PLAT_Gnos
PLAT_Gapp
PLAT_Pvit
PLAT_Mlin
PLAT_Sleu
GAST_Dnot
GAST_Lepi
DICY_Dicy
ENTO_Lxmt
ENTO_Loxp
ENTO_PclF
ENTO_Bgra
CYCL_Symb
CYCL_SyRN
GAST_Mdas
GAST_Megd
ANNE_Diur
ANNE_Loba
ANNE_Myzo
BRYO_Bstl
BRYO_Mmem
BRYO_Fcor
BRYO_Hpac
CHAE_Selg
CHAE_Spdl
ANNE_Mbel
ANNE_Ncae
ANNE_Ssqu
ANNE_Pfoe
ANNE_Hrob
ANNE_Lrub
ANNE_Ctel
ANNE_Spsm
ANNE_Plcs
ANNE_Pamp
ANNE_Phyl
ANNE_Ofus
ANNE_Mjoh
NEME_Hiji
NEME_Buni
NEME_Peeb
NEME_Aaus
NEME_Cham
BRAC_Krub
BRAC_Ttvs
BRAC_Mcdv
BRAC_Hpst
BRAC_Lana
BRAC_Gpyr
BRAC_Nvcn
PHOR_Ppsa
PHOR_Paus
PHOR_Phar
MOLL_Mlab
MOLL_Lott
MOLL_Hphy
MOLL_Cgig
MOLL_Svel
MOLL_Npom
MOLL_Ovul
MOLL_Lvpl
MOLL_Nmna
MOLL_Pcus
MOLL_Phol
MOLL_Lrug
MOLL_Cton
ARTH_Scut
ARTH_Smar
ARTH_Gmar
ARTH_Etai
ARTH_Smim
ARTH_Limu
ARTH_Isca
ARTH_Ains
ARTH_Amel
ARTH_Dmel
ARTH_Fcan
ARTH_Dpul
ARTH_Fmer
ARTH_Cfin
ONYC_Peri
ONYC_Oope
ONYC_Pcap
ONYC_Pept
KINO_Pkie
KINO_Eduj
PRIA_Meio
PRIA_Pcau
ECHI_Ajap
ECHI_Sbri
ECHI_Hgla
ECHI_Spur
ECHI_Lvar
ECHI_Etri
ECHI_Oech
ECHI_Olon
ECHI_Pmin
ECHI_Ojap
HEMI_Pbah
HEMI_Tbah
HEMI_Sbra
HEMI_Skow
HEMI_Cgra
CRAN_Mmus
PRIA_Tubi
CRAN_Hsap
CRAN_Ggal
CRAN_Lcha
CRAN_Cmil
CRAN_Drer
CRAN_Pmar
UROC_Cint
CEPH_Aluc
CEPH_Bflo
ACOE_Conv
ACOE_Sros
ACOE_Nfus
ACOE_Chil
ACOE_Eumc
ACOE_Isop
ACOE_Diog
ACOE_Diol
ACOE_Hmia
GAST_Dpdl
NEMO_Asco
NEMO_Flag
NEMO_Mers
NEMO_Nemw
XENO_XbJC
XENO_Xboc
CNID_Atet
CNID_Hvul
CNID_Csow
CNID_Smel
CNID_Phyd
CNID_Adig
CNID_Nvec
CNID_Gven
PLAC_Tadh
PORI_Cvar
PORI_Psub
PORI_Cele
PORI_Slac
PORI_Aque
PORI_Pfic
PORI_Cnuc
PORI_Ifas
PORI_Avas
PORI_Scil
PORI_Scoa
PORI_Ccla
PORI_Ocar
PORI_Ccan
CTEN_Baby
CTEN_Mlei
CTEN_Pbac
CTEN_Vmul
CTEN_Edun
OUTF_Rirr
OUTF_Rsol
OUTF_Mver
OUTF_Spun
OUTF_Foxy
OUTF_Amac
OUTF_Rall
OUTI_Ttra
OUTI_Falb
OUTI_Apar
OUTI_Sart
OUTI_Mvar
OUTI_Cowc
OUTC_Sros
OUTC_Mbre
0.3

S2

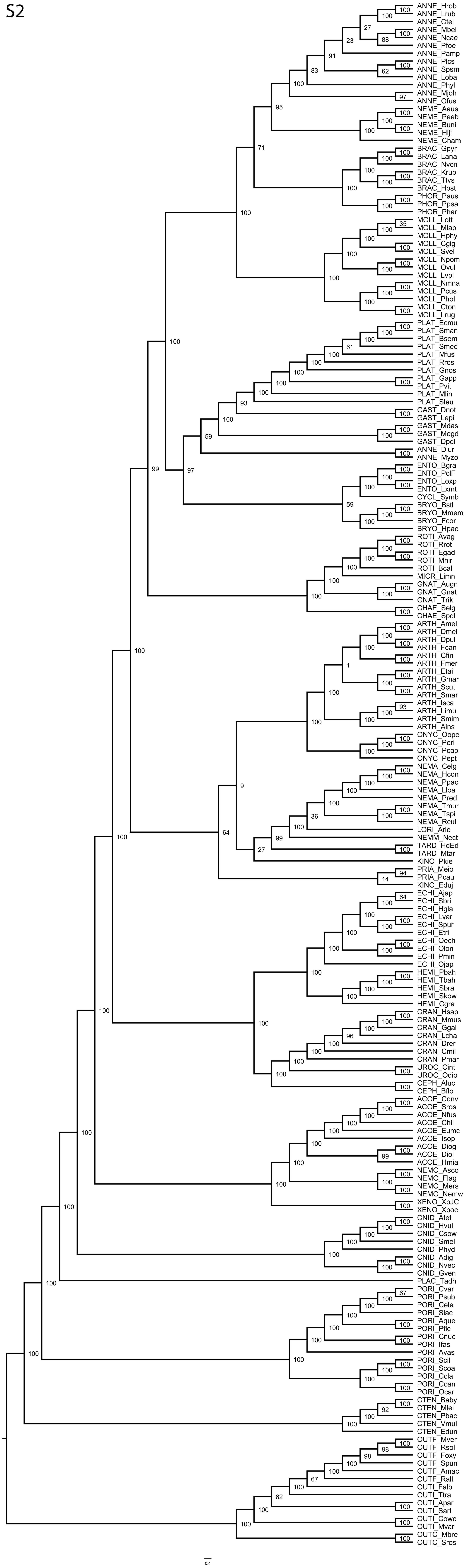
ANNE_Hrob
ANNE_Lrub
ANNE_Ctel
ANNE_Mbel
ANNE_Ncae
ANNE_Pfoe
ANNE_Pamp
ANNE_Plcs
ANNE_Spsm
ANNE_Loba
ANNE_Phyl
ANNE_Mjoh
ANNE_Ofus
NEME_Aaus
NEME_Peeb
NEME_Buni
NEME_Hiji
NEME_Cham
BRAC_Gpyr
BRAC_Lana
BRAC_Nvcn
BRAC_Krub
BRAC_Ttvs
BRAC_Hpst
PHOR_Paus
PHOR_Ppsa
PHOR_Phar
MOLL_Lott
MOLL_Mlab
MOLL_Hphy
MOLL_Cgig
MOLL_Svel
MOLL_Npom
MOLL_Ovul
MOLL_Lvpl
MOLL_Nmna
MOLL_Pcus
MOLL_Phol
MOLL_Cton
MOLL_Lrug
PLAT_Ecmu
PLAT_Sman
PLAT_Bsem
PLAT_Smed
PLAT_Mfus
PLAT_Rros
PLAT_Gnos
PLAT_Gapp
PLAT_Pvit
PLAT_Mlin
PLAT_Sleu
GAST_Dnot
GAST_Lepi
GAST_Mdas
GAST_Megd
GAST_Dpdl
ANNE_Diur
ANNE_Myzo
ENTO_Bgra
ENTO_PclF
ENTO_Loxp
ENTO_Lxmt
CYCL_Symb
BRYO_Bstl
BRYO_Mmem
BRYO_Fcor
BRYO_Hpac
ROTI_Avag
ROTI_Rrot
ROTI_Egad
ROTI_Mhir
ROTI_Bcal
MICR_Limn
GNAT_Augn
GNAT_Gnat
GNAT_Trik
CHAE_Selg
CHAE_Spdl
ARTH_Amel
ARTH_Dmel
ARTH_Dpul
ARTH_Fcan
ARTH_Cfin
ARTH_Fmer
ARTH_Etai
ARTH_Gmar
ARTH_Scut
ARTH_Smar
ARTH_Isca
ARTH_Limu
ARTH_Smim
ARTH_Ains
ONYC_Oope
ONYC_Peri
ONYC_Pcap
ONYC_Pept
NEMA_Celg
NEMA_Hcon
NEMA_Ppac
NEMA_Lloa
NEMA_Pred
NEMA_Tmur
NEMA_Tspi
NEMA_Rcul
LORI_Arlc
NEMM_Nect
TARD_HdEd
TARD_Mtar
KINO_Pkie
PRIA_Meio
PRIA_Pcau
KINO_Eduj
ECHI_Ajap
ECHI_Sbri
ECHI_Hgla
ECHI_Lvar
ECHI_Spur
ECHI_Etri
ECHI_Oech
ECHI_Olon
ECHI_Pmin
ECHI_Ojap
HEMI_Pbah
HEMI_Tbah
HEMI_Sbra
HEMI_Skow
HEMI_Cgra
CRAN_Hsap
CRAN_Mmus
CRAN_Ggal
CRAN_Lcha
CRAN_Drer
CRAN_Cmil
CRAN_Pmar
UROC_Cint
UROC_Odio
CEPH_Aluc
CEPH_Bflo
ACOE_Conv
ACOE_Sros
ACOE_Nfus
ACOE_Chil
ACOE_Eumc
ACOE_Isop
ACOE_Diog
ACOE_Diol
ACOE_Hmia
NEMO_Asco
NEMO_Flag
NEMO_Mers
NEMO_Nemw
XENO_XbJC
XENO_Xboc
CNID_Atet
CNID_Hvul
CNID_Csow
CNID_Smel
CNID_Phyd
CNID_Adig
CNID_Nvec
CNID_Gven
PLAC_Tadh
PORI_Cvar
PORI_Psub
PORI_Cele
PORI_Slac
PORI_Aque
PORI_Pfic
PORI_Cnuc
PORI_Ifas
PORI_Avas
PORI_Scil
PORI_Scoa
PORI_Ccla
PORI_Ccan
PORI_Ocar
CTEN_Baby
CTEN_Mlei
CTEN_Pbac
CTEN_Vmul
CTEN_Edun
OUTF_Mver
OUTF_Rsol
OUTF_Foxy
OUTF_Spun
OUTF_Amac
OUTF_Rall
OUTI_Falb
OUTI_Ttra
OUTI_Apar
OUTI_Sart
OUTI_Cowc
OUTI_Mvar
OUTC_Mbre
OUTC_Sros
0.4

S3

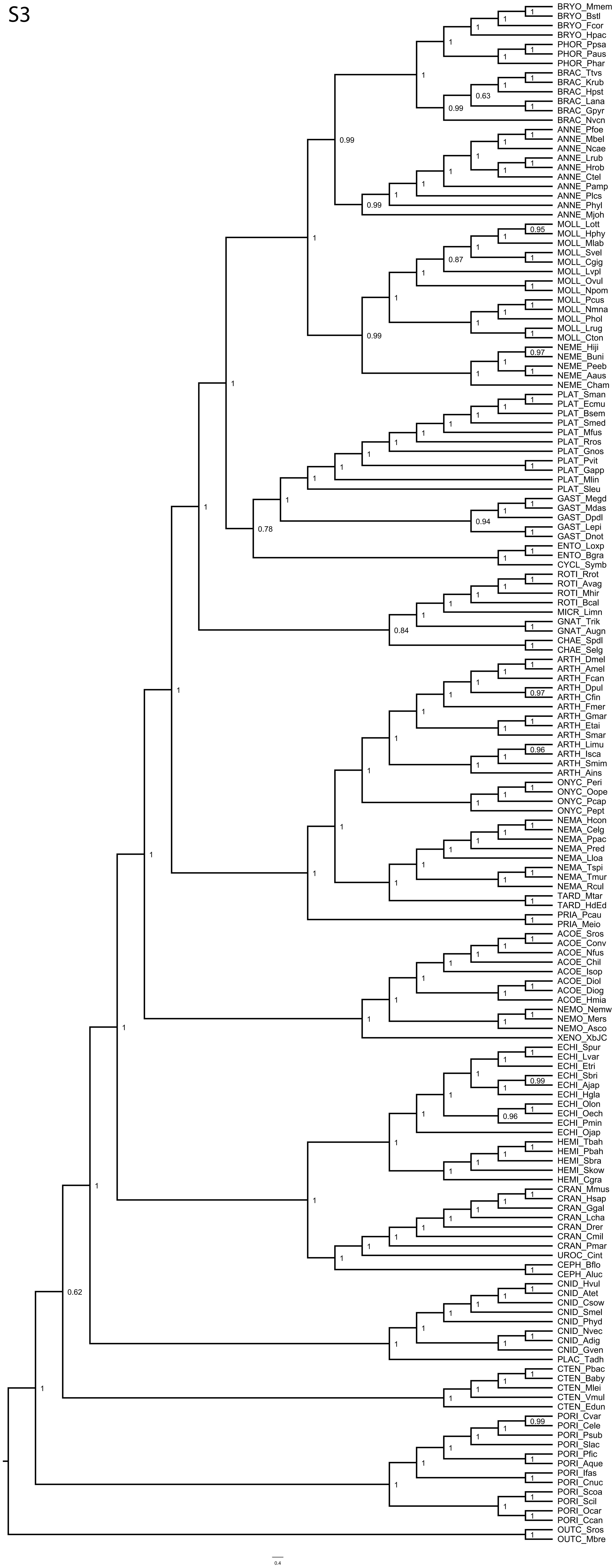

BRYO_Mmem
BRYO_Bstl
BRYO_Fcor
BRYO_Hpac
PHOR_Ppsa
PHOR_Paus
PHOR_Phar
BRAC_Ttvs
BRAC_Krub
BRAC_Hpst
BRAC_Lana
BRAC_Gpyr
BRAC_Nvcn
ANNE_Pfoe
ANNE_Mbel
ANNE_Ncae
ANNE_Lrub
ANNE_Hrob
ANNE_Ctel
ANNE_Pamp
ANNE_Plcs
ANNE_Phyl
ANNE_Mjoh
MOLL_Lott
MOLL_Hphy
MOLL_Mlab
MOLL_Svel
MOLL_Cgig
MOLL_Lvpl
MOLL_Ovul
MOLL_Npom
MOLL_Pcus
MOLL_Nmna
MOLL_Phol
MOLL_Lrug
MOLL_Cton
NEME_Hiji
NEME_Buni
NEME_Peeb
NEME_Aaus
NEME_Cham
PLAT_Sman
PLAT_Ecmu
PLAT_Bsem
PLAT_Smed
PLAT_Mfus
PLAT_Rros
PLAT_Gnos
PLAT_Pvit
PLAT_Gapp
PLAT_Mlin
PLAT_Sleu
GAST_Megd
GAST_Mdas
GAST_Dpdl
GAST_Lepi
GAST_Dnot
ENTO_Loxp
ENTO_Bgra
CYCL_Symb
ROTI_Rrot
ROTI_Avag
ROTI_Mhir
ROTI_Bcal
MICR_Limn
GNAT_Trik
GNAT_Augn
CHAE_Spdl
CHAE_Selg
ARTH_Dmel
ARTH_Amel
ARTH_Fcan
ARTH_Dpul
ARTH_Cfin
ARTH_Fmer
ARTH_Gmar
ARTH_Etai
ARTH_Smar
ARTH_Limu
ARTH_Isca
ARTH_Smim
ARTH_Ains
ONYC_Peri
ONYC_Oope
ONYC_Pcap
ONYC_Pept
NEMA_Hcon
NEMA_Celg
NEMA_Ppac
NEMA_Pred
NEMA_Lloa
NEMA_Tspi
NEMA_Tmur
NEMA_Rcul
TARD_Mtar
TARD_HdEd
PRIA_Pcau
PRIA_Meio
ACOE_Sros
ACOE_Conv
ACOE_Nfus
ACOE_Chil
ACOE_Isop
ACOE_Diol
ACOE_Diog
ACOE_Hmia
NEMO_Nemw
NEMO_Mers
NEMO_Asco
XENO_XbJC
ECHI_Spur
ECHI_Lvar
ECHI_Etri
ECHI_Sbri
ECHI_Ajap
ECHI_Hgla
ECHI_Olon
ECHI_Oech
ECHI_Pmin
ECHI_Ojap
HEMI_Tbah
HEMI_Pbah
HEMI_Sbra
HEMI_Skow
HEMI_Cgra
CRAN_Mmus
CRAN_Hsap
CRAN_Ggal
CRAN_Lcha
CRAN_Drer
CRAN_Cmil
CRAN_Pmar
UROC_Cint
CEPH_Bflo
CEPH_Aluc
CNID_Hvul
CNID_Atet
CNID_Csow
CNID_Smel
CNID_Phyd
CNID_Nvec
CNID_Adig
CNID_Gven
PLAC_Tadh
CTEN_Pbac
CTEN_Baby
CTEN_Mlei
CTEN_Vmul
CTEN_Edun
PORI_Cvar
PORI_Cele
PORI_Psub
PORI_Slac
PORI_Pfic
PORI_Aque
PORI_Ifas
PORI_Cnuc
PORI_Scoa
PORI_Scil
PORI_Ocar
PORI_Ccan
OUTC_Sros
OUTC_Mbre
0.4

S4

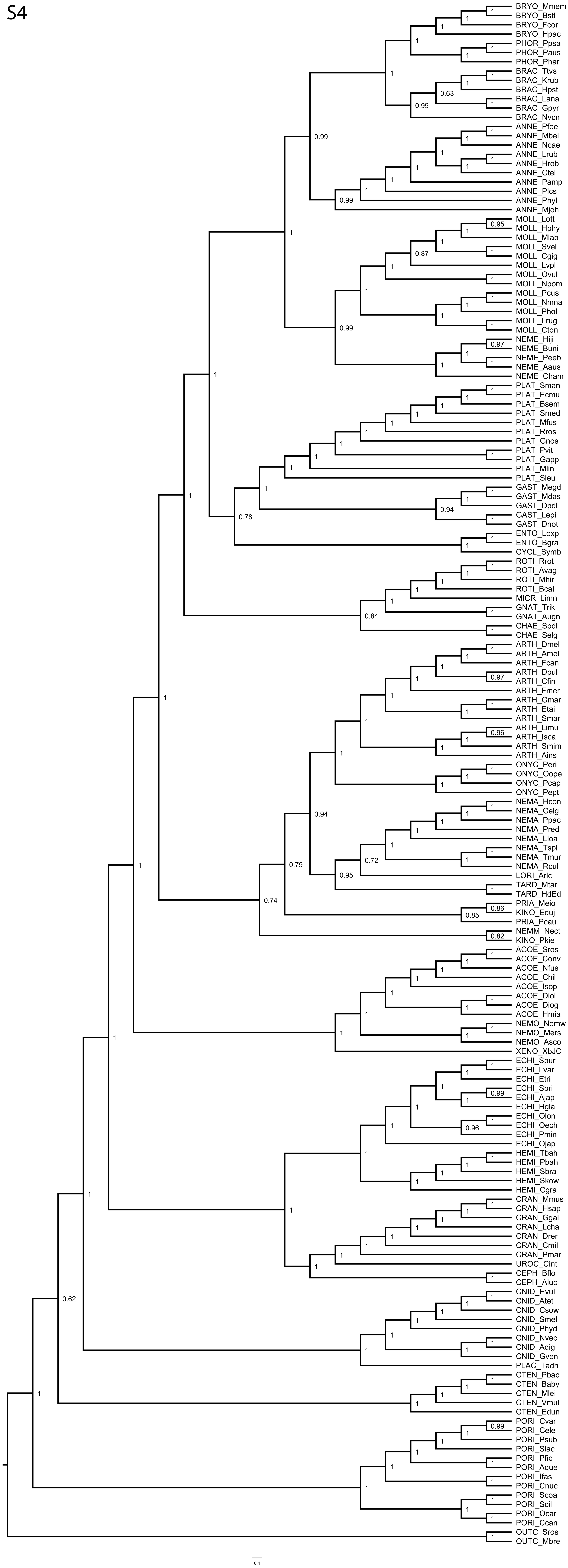
BRYO_Mmem
BRYO_Bstl
BRYO_Fcor
BRYO_Hpac
PHOR_Ppsa
PHOR_Paus
PHOR_Phar
BRAC_Ttvs
BRAC_Krub
BRAC_Hpst
BRAC_Lana
BRAC_Gpyr
BRAC_Nvcn
ANNE_Pfoe
ANNE_Mbel
ANNE_Ncae
ANNE_Lrub
ANNE_Hrob
ANNE_Ctel
ANNE_Pamp
ANNE_Plcs
ANNE_Phyl
ANNE_Mjoh
MOLL_Lott
MOLL_Hphy
MOLL_Mlab
MOLL_Svel
MOLL_Cgig
MOLL_Lvpl
MOLL_Ovul
MOLL_Npom
MOLL_Pcus
MOLL_Nmna
MOLL_Phol
MOLL_Lrug
MOLL_Cton
NEME_Hiji
NEME_Buni
NEME_Peeb
NEME_Aaus
NEME_Cham
PLAT_Sman
PLAT_Ecmu
PLAT_Bsem
PLAT_Smed
PLAT_Mfus
PLAT_Rros
PLAT_Gnos
PLAT_Pvit
PLAT_Gapp
PLAT_Mlin
PLAT_Sleu
GAST_Megd
GAST_Mdas
GAST_Dpdl
GAST_Lepi
GAST_Dnot
ENTO_Loxp
ENTO_Bgra
CYCL_Symb
ROTI_Rrot
ROTI_Avag
ROTI_Mhir
ROTI_Bcal
MICR_Limn
GNAT_Trik
GNAT_Augn
CHAE_Spdl
CHAE_Selg
ARTH_Dmel
ARTH_Amel
ARTH_Fcan
ARTH_Dpul
ARTH_Cfin
ARTH_Fmer
ARTH_Gmar
ARTH_Etai
ARTH_Smar
ARTH_Limu
ARTH_Isca
ARTH_Smim
ARTH_Ains
ONYC_Peri
ONYC_Oope
ONYC_Pcap
ONYC_Pept
NEMA_Hcon
NEMA_Celg
NEMA_Ppac
NEMA_Pred
NEMA_Lloa
NEMA_Tspi
NEMA_Tmur
NEMA_Rcul
LORI_Arlc
TARD_Mtar
TARD_HdEd
PRIA_Meio
KINO_Eduj
PRIA_Pcau
NEMM_Nect
KINO_Pkie
ACOE_Sros
ACOE_Conv
ACOE_Nfus
ACOE_Chil
ACOE_Isop
ACOE_Diol
ACOE_Diog
ACOE_Hmia
NEMO_Nemw
NEMO_Mers
NEMO_Asco
XENO_XbJC
ECHI_Spur
ECHI_Lvar
ECHI_Etri
ECHI_Sbri
ECHI_Ajap
ECHI_Hgla
ECHI_Olon
ECHI_Oech
ECHI_Pmin
ECHI_Ojap
HEMI_Tbah
HEMI_Pbah
HEMI_Sbra
HEMI_Skow
HEMI_Cgra
CRAN_Mmus
CRAN_Hsap
CRAN_Ggal
CRAN_Lcha
CRAN_Drer
CRAN_Cmil
CRAN_Pmar
UROC_Cint
CEPH_Bflo
CEPH_Aluc
CNID_Hvul
CNID_Atet
CNID_Csow
CNID_Smel
CNID_Phyd
CNID_Nvec
CNID_Adig
CNID_Gven
PLAC_Tadh
CTEN_Pbac
CTEN_Baby
CTEN_Mlei
CTEN_Vmul
CTEN_Edun
PORI_Cvar
PORI_Cele
PORI_Psub
PORI_Slac
PORI_Pfic
PORI_Aque
PORI_Ifas
PORI_Cnuc
PORI_Scoa
PORI_Scil
PORI_Ocar
PORI_Ccan
OUTC_Sros
OUTC_Mbre
0.4

S5

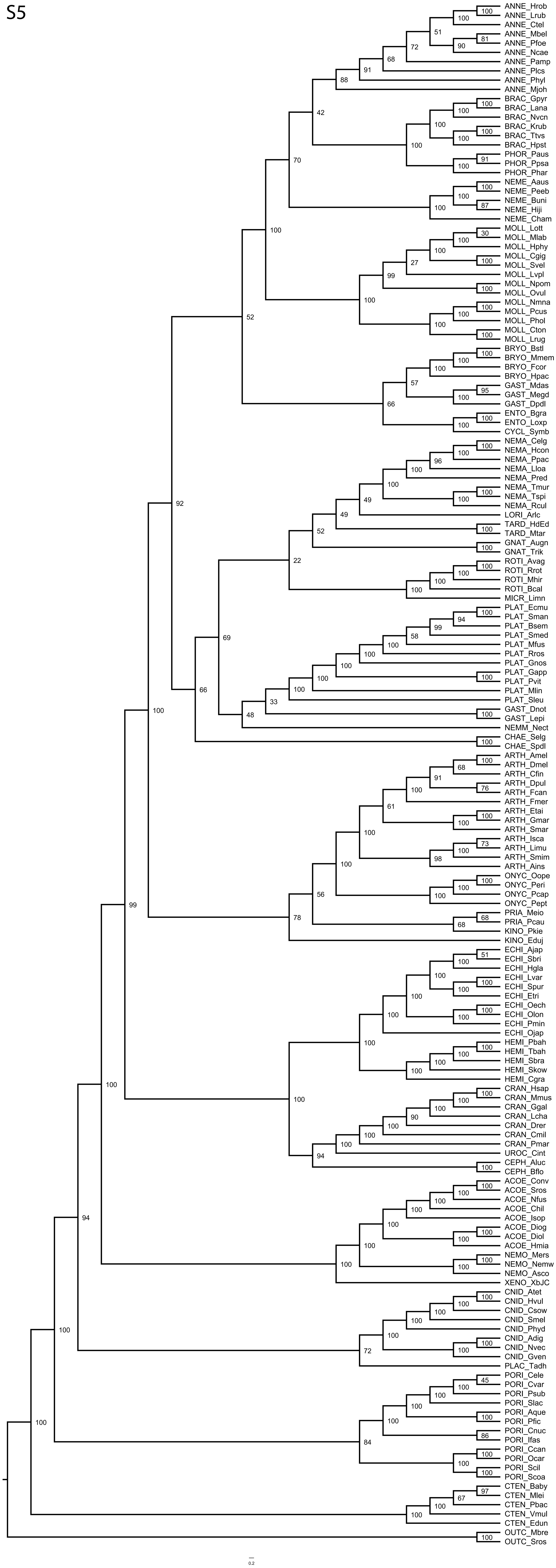
ANNE_Hrob
ANNE_Lrub
ANNE_Ctel
ANNE_Mbel
ANNE_Pfoe
ANNE_Ncae
ANNE_Pamp
ANNE_Plcs
ANNE_Phyl
ANNE_Mjoh
BRAC_Gpyr
BRAC_Lana
BRAC_Nvcn
BRAC_Krub
BRAC_Ttvs
BRAC_Hpst
PHOR_Paus
PHOR_Ppsa
PHOR_Phar
NEME_Aaus
NEME_Peeb
NEME_Buni
NEME_Hiji
NEME_Cham
MOLL_Lott
MOLL_Mlab
MOLL_Hphy
MOLL_Cgig
MOLL_Svel
MOLL_Lvpl
MOLL_Npom
MOLL_Ovul
MOLL_Nmna
MOLL_Pcus
MOLL_Phol
MOLL_Cton
MOLL_Lrug
BRYO_Bstl
BRYO_Mmem
BRYO_Fcor
BRYO_Hpac
GAST_Mdas
GAST_Megd
GAST_Dpdl
ENTO_Bgra
ENTO_Loxp
CYCL_Symb
NEMA_Celg
NEMA_Hcon
NEMA_Ppac
NEMA_Lloa
NEMA_Pred
NEMA_Tmur
NEMA_Tspi
NEMA_Rcul
LORI_Arlc
TARD_HdEd
TARD_Mtar
GNAT_Augn
GNAT_Trik
ROTI_Avag
ROTI_Rrot
ROTI_Mhir
ROTI_Bcal
MICR_Limn
PLAT_Ecmu
PLAT_Sman
PLAT_Bsem
PLAT_Smed
PLAT_Mfus
PLAT_Rros
PLAT_Gnos
PLAT_Gapp
PLAT_Pvit
PLAT_Mlin
PLAT_Sleu
GAST_Dnot
GAST_Lepi
NEMM_Nect
CHAE_Selg
CHAE_Spdl
ARTH_Amel
ARTH_Dmel
ARTH_Cfin
ARTH_Dpul
ARTH_Fcan
ARTH_Fmer
ARTH_Etai
ARTH_Gmar
ARTH_Smar
ARTH_Isca
ARTH_Limu
ARTH_Smim
ARTH_Ains
ONYC_Oope
ONYC_Peri
ONYC_Pcap
ONYC_Pept
PRIA_Meio
PRIA_Pcau
KINO_Pkie
KINO_Eduj
ECHI_Ajap
ECHI_Sbri
ECHI_Hgla
ECHI_Lvar
ECHI_Spur
ECHI_Etri
ECHI_Oech
ECHI_Olon
ECHI_Pmin
ECHI_Ojap
HEMI_Pbah
HEMI_Tbah
HEMI_Sbra
HEMI_Skow
HEMI_Cgra
CRAN_Hsap
CRAN_Mmus
CRAN_Ggal
CRAN_Lcha
CRAN_Drer
CRAN_Cmil
CRAN_Pmar
UROC_Cint
CEPH_Aluc
CEPH_Bflo
ACOE_Conv
ACOE_Sros
ACOE_Nfus
ACOE_Chil
ACOE_Isop
ACOE_Diog
ACOE_Diol
ACOE_Hmia
NEMO_Mers
NEMO_Nemw
NEMO_Asco
XENO_XbJC
CNID_Atet
CNID_Hvul
CNID_Csow
CNID_Smel
CNID_Phyd
CNID_Adig
CNID_Nvec
CNID_Gven
PLAC_Tadh
PORI_Cele
PORI_Cvar
PORI_Psub
PORI_Slac
PORI_Aque
PORI_Pfic
PORI_Cnuc
PORI_Ifas
PORI_Ccan
PORI_Ocar
PORI_Scil
PORI_Scoa
CTEN_Baby
CTEN_Mlei
CTEN_Pbac
CTEN_Vmul
CTEN_Edun
OUTC_Mbre
OUTC_Sros
0.2

S6

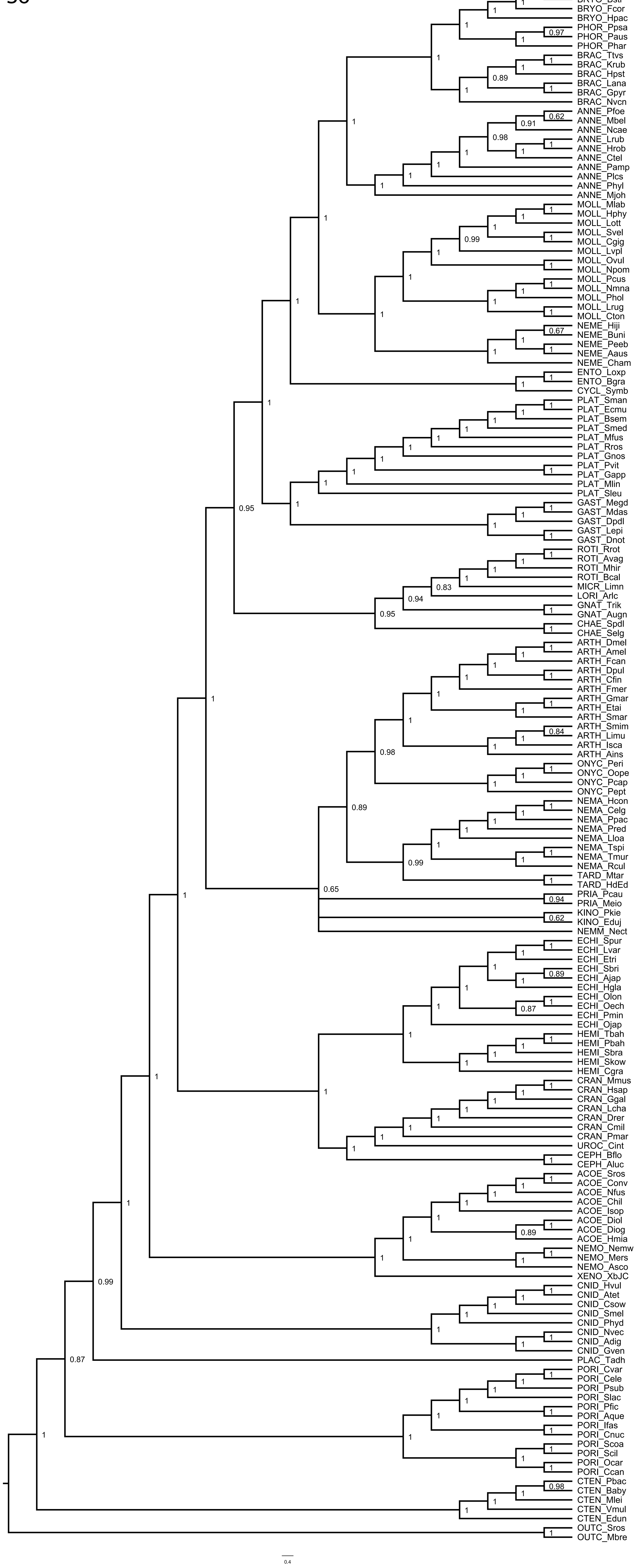
BRYO_Mmem
BRYO_Bstl
BRYO_Fcor
BRYO_Hpac
PHOR_Ppsa
PHOR_Paus
PHOR_Phar
BRAC_Ttvs
BRAC_Krub
BRAC_Hpst
BRAC_Lana
BRAC_Gpyr
BRAC_Nvcn
ANNE_Pfoe
ANNE_Mbel
ANNE_Ncae
ANNE_Lrub
ANNE_Hrob
ANNE_Ctel
ANNE_Pamp
ANNE_Plcs
ANNE_Phyl
ANNE_Mjoh
MOLL_Mlab
MOLL_Hphy
MOLL_Lott
MOLL_Svel
MOLL_Cgig
MOLL_Lvpl
MOLL_Ovul
MOLL_Npom
MOLL_Pcus
MOLL_Nmna
MOLL_Phol
MOLL_Lrug
MOLL_Cton
NEME_Hiji
NEME_Buni
NEME_Peeb
NEME_Aaus
NEME_Cham
ENTO_Loxp
ENTO_Bgra
CYCL_Symb
PLAT_Sman
PLAT_Ecmu
PLAT_Bsem
PLAT_Smed
PLAT_Mfus
PLAT_Rros
PLAT_Gnos
PLAT_Pvit
PLAT_Gapp
PLAT_Mlin
PLAT_Sleu
GAST_Megd
GAST_Mdas
GAST_Dpdl
GAST_Lepi
GAST_Dnot
ROTI_Rrot
ROTI_Avag
ROTI_Mhir
ROTI_Bcal
MICR_Limn
LORI_Arlc
GNAT_Trik
GNAT_Augn
CHAE_Spdl
CHAE_Selg
ARTH_Dmel
ARTH_Amel
ARTH_Fcan
ARTH_Dpul
ARTH_Cfin
ARTH_Fmer
ARTH_Gmar
ARTH_Etai
ARTH_Smar
ARTH_Smim
ARTH_Limu
ARTH_Isca
ARTH_Ains
ONYC_Peri
ONYC_Oope
ONYC_Pcap
ONYC_Pept
NEMA_Hcon
NEMA_Celg
NEMA_Ppac
NEMA_Pred
NEMA_Lloa
NEMA_Tspi
NEMA_Tmur
NEMA_Rcul
TARD_Mtar
TARD_HdEd
PRIA_Pcau
PRIA_Meio
KINO_Pkie
KINO_Eduj
NEMM_Nect
ECHI_Spur
ECHI_Lvar
ECHI_Etri
ECHI_Sbri
ECHI_Ajap
ECHI_Hgla
ECHI_Olon
ECHI_Oech
ECHI_Pmin
ECHI_Ojap
HEMI_Tbah
HEMI_Pbah
HEMI_Sbra
HEMI_Skow
HEMI_Cgra
CRAN_Mmus
CRAN_Hsap
CRAN_Ggal
CRAN_Lcha
CRAN_Drer
CRAN_Cmil
CRAN_Pmar
UROC_Cint
CEPH_Bflo
CEPH_Aluc
ACOE_Sros
ACOE_Conv
ACOE_Nfus
ACOE_Chil
ACOE_Isop
ACOE_Diol
ACOE_Diog
ACOE_Hmia
NEMO_Nemw
NEMO_Mers
NEMO_Asco
XENO_XbJC
CNID_Hvul
CNID_Atet
CNID_Csow
CNID_Smel
CNID_Phyd
CNID_Nvec
CNID_Adig
CNID_Gven
PLAC_Tadh
PORI_Cvar
PORI_Cele
PORI_Psub
PORI_Slac
PORI_Pfic
PORI_Aque
PORI_Ifas
PORI_Cnuc
PORI_Scoa
PORI_Scil
PORI_Ocar
PORI_Ccan
CTEN_Pbac
CTEN_Baby
CTEN_Mlei
CTEN_Vmul
CTEN_Edun
OUTC_Sros
OUTC_Mbre
0.4

S7

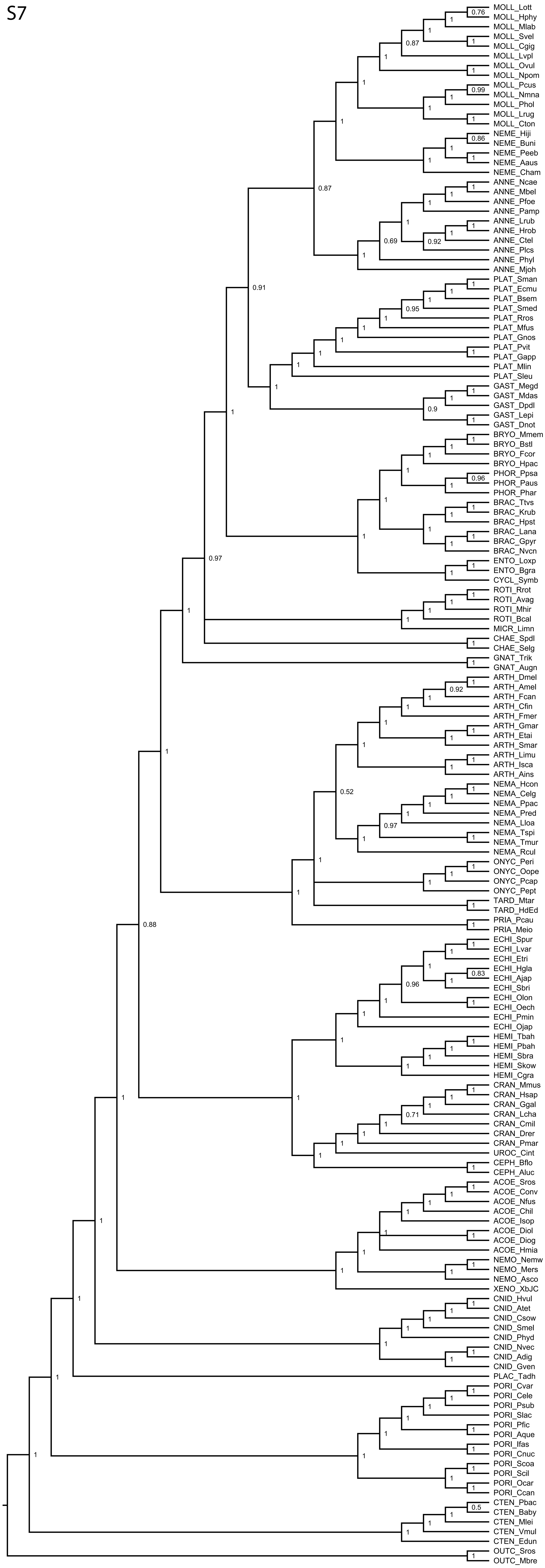

MOLL_Lott
MOLL_Hphy
MOLL_Mlab
MOLL_Svel
MOLL_Cgig
MOLL_Lvpl
MOLL_Ovul
MOLL_Npom
MOLL_Pcus
MOLL_Nmna
MOLL_Phol
MOLL_Lrug
MOLL_Cton
NEME_Hiji
NEME_Buni
NEME_Peeb
NEME_Aaus
NEME_Cham
ANNE_Ncae
ANNE_Mbel
ANNE_Pfoe
ANNE_Pamp
ANNE_Lrub
ANNE_Hrob
ANNE_Ctel
ANNE_Plcs
ANNE_Phyl
ANNE_Mjoh
PLAT_Sman
PLAT_Ecmu
PLAT_Bsem
PLAT_Smed
PLAT_Rros
PLAT_Mfus
PLAT_Gnos
PLAT_Pvit
PLAT_Gapp
PLAT_Mlin
PLAT_Sleu
GAST_Megd
GAST_Mdas
GAST_Dpdl
GAST_Lepi
GAST_Dnot
BRYO_Mmem
BRYO_Bstl
BRYO_Fcor
BRYO_Hpac
PHOR_Ppsa
PHOR_Paus
PHOR_Phar
BRAC_Ttvs
BRAC_Krub
BRAC_Hpst
BRAC_Lana
BRAC_Gpyr
BRAC_Nvcn
ENTO_Loxp
ENTO_Bgra
CYCL_Symb
ROTI_Rrot
ROTI_Avag
ROTI_Mhir
ROTI_Bcal
MICR_Limn
CHAE_Spdl
CHAE_Selg
GNAT_Trik
GNAT_Augn
ARTH_Dmel
ARTH_Amel
ARTH_Fcan
ARTH_Cfin
ARTH_Fmer
ARTH_Gmar
ARTH_Etai
ARTH_Smar
ARTH_Limu
ARTH_Isca
ARTH_Ains
NEMA_Hcon
NEMA_Celg
NEMA_Ppac
NEMA_Pred
NEMA_Lloa
NEMA_Tspi
NEMA_Tmur
NEMA_Rcul
ONYC_Peri
ONYC_Oope
ONYC_Pcap
ONYC_Pept
TARD_Mtar
TARD_HdEd
PRIA_Pcau
PRIA_Meio
ECHI_Spur
ECHI_Lvar
ECHI_Etri
ECHI_Hgla
ECHI_Ajap
ECHI_Sbri
ECHI_Olon
ECHI_Oech
ECHI_Pmin
ECHI_Ojap
HEMI_Tbah
HEMI_Pbah
HEMI_Sbra
HEMI_Skow
HEMI_Cgra
CRAN_Mmus
CRAN_Hsap
CRAN_Ggal
CRAN_Lcha
CRAN_Cmil
CRAN_Drer
CRAN_Pmar
UROC_Cint
CEPH_Bflo
CEPH_Aluc
ACOE_Sros
ACOE_Conv
ACOE_Nfus
ACOE_Chil
ACOE_Isop
ACOE_Diol
ACOE_Diog
ACOE_Hmia
NEMO_Nemw
NEMO_Mers
NEMO_Asco
XENO_XbJC
CNID_Hvul
CNID_Atet
CNID_Csow
CNID_Smel
CNID_Phyd
CNID_Nvec
CNID_Adig
CNID_Gven
PLAC_Tadh
PORI_Cvar
PORI_Cele
PORI_Psub
PORI_Slac
PORI_Pfic
PORI_Aque
PORI_Ifas
PORI_Cnuc
PORI_Scoa
PORI_Scil
PORI_Ocar
PORI_Ccan
CTEN_Pbac
CTEN_Baby
CTEN_Mlei
CTEN_Vmul
CTEN_Edun
OUTC_Sros
OUTC_Mbre

S8

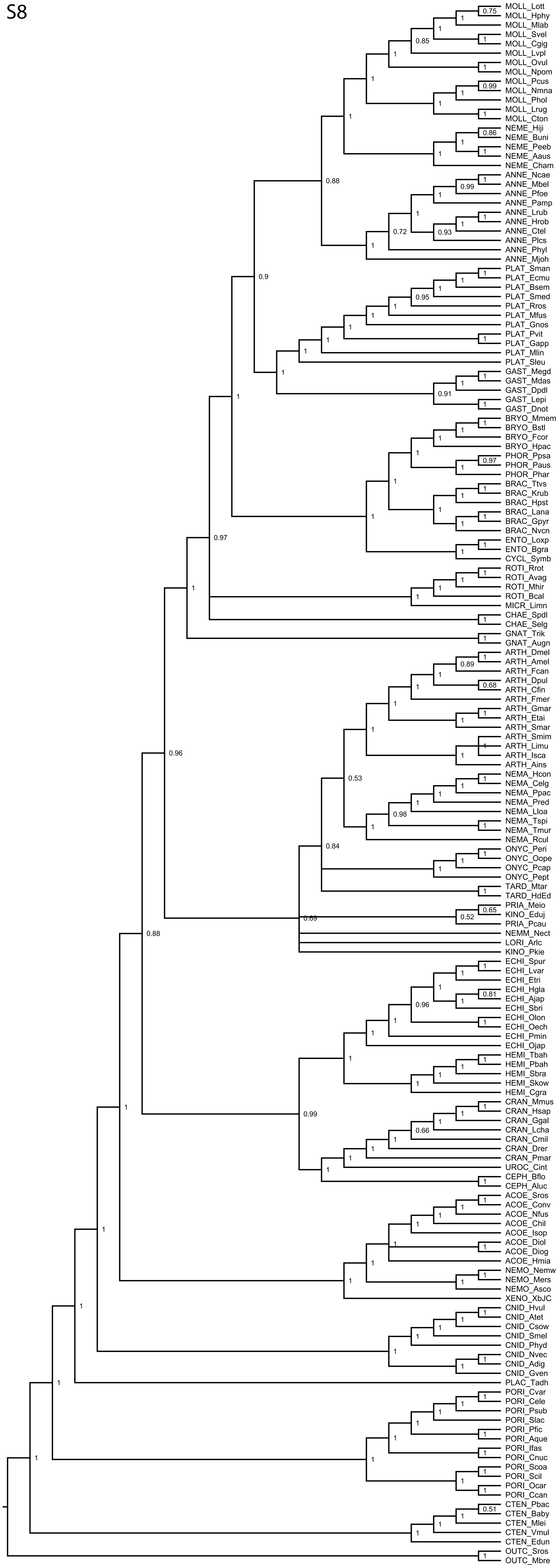
MOLL_Lott
MOLL_Hphy
MOLL_Mlab
MOLL_Svel
MOLL_Cgig
MOLL_Lvpl
MOLL_Ovul
MOLL_Npom
MOLL_Pcus
MOLL_Nmna
MOLL_Phol
MOLL_Lrug
MOLL_Cton
NEME_Hiji
NEME_Buni
NEME_Peeb
NEME_Aaus
NEME_Cham
ANNE_Ncae
ANNE_Mbel
ANNE_Pfoe
ANNE_Pamp
ANNE_Lrub
ANNE_Hrob
ANNE_Ctel
ANNE_Plcs
ANNE_Phyl
ANNE_Mjoh
PLAT_Sman
PLAT_Ecmu
PLAT_Bsem
PLAT_Smed
PLAT_Rros
PLAT_Mfus
PLAT_Gnos
PLAT_Pvit
PLAT_Gapp
PLAT_Mlin
PLAT_Sleu
GAST_Megd
GAST_Mdas
GAST_Dpdl
GAST_Lepi
GAST_Dnot
BRYO_Mmem
BRYO_Bstl
BRYO_Fcor
BRYO_Hpac
PHOR_Ppsa
PHOR_Paus
PHOR_Phar
BRAC_Ttvs
BRAC_Krub
BRAC_Hpst
BRAC_Lana
BRAC_Gpyr
BRAC_Nvcn
ENTO_Loxp
ENTO_Bgra
CYCL_Symb
ROTI_Rrot
ROTI_Avag
ROTI_Mhir
ROTI_Bcal
MICR_Limn
CHAE_Spdl
CHAE_Selg
GNAT_Trik
GNAT_Augn
ARTH_Dmel
ARTH_Amel
ARTH_Fcan
ARTH_Dpul
ARTH_Cfin
ARTH_Fmer
ARTH_Gmar
ARTH_Etai
ARTH_Smar
ARTH_Smim
ARTH_Limu
ARTH_Isca
ARTH_Ains
NEMA_Hcon
NEMA_Celg
NEMA_Ppac
NEMA_Pred
NEMA_Lloa
NEMA_Tspi
NEMA_Tmur
NEMA_Rcul
ONYC_Peri
ONYC_Oope
ONYC_Pcap
ONYC_Pept
TARD_Mtar
TARD_HdEd
PRIA_Meio
KINO_Eduj
PRIA_Pcau
NEMM_Nect
LORI_Arlc
KINO_Pkie
ECHI_Spur
ECHI_Lvar
ECHI_Etri
ECHI_Hgla
ECHI_Ajap
ECHI_Sbri
ECHI_Olon
ECHI_Oech
ECHI_Pmin
ECHI_Ojap
HEMI_Tbah
HEMI_Pbah
HEMI_Sbra
HEMI_Skow
HEMI_Cgra
CRAN_Mmus
CRAN_Hsap
CRAN_Ggal
CRAN_Lcha
CRAN_Cmil
CRAN_Drer
CRAN_Pmar
UROC_Cint
CEPH_Bflo
CEPH_Aluc
ACOE_Sros
ACOE_Conv
ACOE_Nfus
ACOE_Chil
ACOE_Isop
ACOE_Diol
ACOE_Diog
ACOE_Hmia
NEMO_Nemw
NEMO_Mers
NEMO_Asco
XENO_XbJC
CNID_Hvul
CNID_Atet
CNID_Csow
CNID_Smel
CNID_Phyd
CNID_Nvec
CNID_Adig
CNID_Gven
PLAC_Tadh
PORI_Cvar
PORI_Cele
PORI_Psub
PORI_Slac
PORI_Pfic
PORI_Aque
PORI_Ifas
PORI_Cnuc
PORI_Scoa
PORI_Scil
PORI_Ocar
PORI_Ccan
CTEN_Pbac
CTEN_Baby
CTEN_Mlei
CTEN_Vmul
CTEN_Edun
OUTC_Sros
OUTC_Mbre

S9

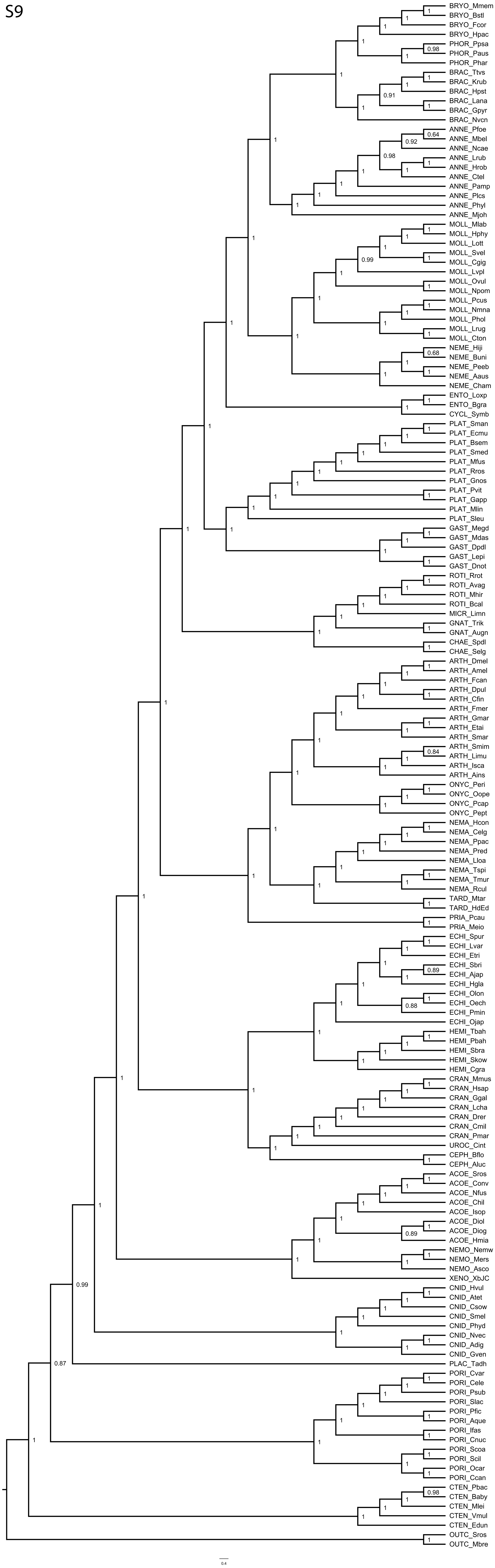

BRYO_Mmem
BRYO_Bstl
BRYO_Fcor
BRYO_Hpac
PHOR_Ppsa
PHOR_Paus
PHOR_Phar
BRAC_Ttvs
BRAC_Krub
BRAC_Hpst
BRAC_Lana
BRAC_Gpyr
BRAC_Nvcn
ANNE_Pfoe
ANNE_Mbel
ANNE_Ncae
ANNE_Lrub
ANNE_Hrob
ANNE_Ctel
ANNE_Pamp
ANNE_Plcs
ANNE_Phyl
ANNE_Mjoh
MOLL_Mlab
MOLL_Hphy
MOLL_Lott
MOLL_Svel
MOLL_Cgig
MOLL_Lvpl
MOLL_Ovul
MOLL_Npom
MOLL_Pcus
MOLL_Nmna
MOLL_Phol
MOLL_Lrug
MOLL_Cton
NEME_Hiji
NEME_Buni
NEME_Peeb
NEME_Aaus
NEME_Cham
ENTO_Loxp
ENTO_Bgra
CYCL_Symb
PLAT_Sman
PLAT_Ecmu
PLAT_Bsem
PLAT_Smed
PLAT_Mfus
PLAT_Rros
PLAT_Gnos
PLAT_Pvit
PLAT_Gapp
PLAT_Mlin
PLAT_Sleu
GAST_Megd
GAST_Mdas
GAST_Dpdl
GAST_Lepi
GAST_Dnot
ROTI_Rrot
ROTI_Avag
ROTI_Mhir
ROTI_Bcal
MICR_Limn
GNAT_Trik
GNAT_Augn
CHAE_Spdl
CHAE_Selg
ARTH_Dmel
ARTH_Amel
ARTH_Fcan
ARTH_Dpul
ARTH_Cfin
ARTH_Fmer
ARTH_Gmar
ARTH_Etai
ARTH_Smar
ARTH_Smim
ARTH_Limu
ARTH_Isca
ARTH_Ains
ONYC_Peri
ONYC_Oope
ONYC_Pcap
ONYC_Pept
NEMA_Hcon
NEMA_Celg
NEMA_Ppac
NEMA_Pred
NEMA_Lloa
NEMA_Tspi
NEMA_Tmur
NEMA_Rcul
TARD_Mtar
TARD_HdEd
PRIA_Pcau
PRIA_Meio
ECHI_Spur
ECHI_Lvar
ECHI_Etri
ECHI_Sbri
ECHI_Ajap
ECHI_Hgla
ECHI_Olon
ECHI_Oech
ECHI_Pmin
ECHI_Ojap
HEMI_Tbah
HEMI_Pbah
HEMI_Sbra
HEMI_Skow
HEMI_Cgra
CRAN_Mmus
CRAN_Hsap
CRAN_Ggal
CRAN_Lcha
CRAN_Drer
CRAN_Cmil
CRAN_Pmar
UROC_Cint
CEPH_Bflo
CEPH_Aluc
ACOE_Sros
ACOE_Conv
ACOE_Nfus
ACOE_Chil
ACOE_Isop
ACOE_Diol
ACOE_Diog
ACOE_Hmia
NEMO_Nemw
NEMO_Mers
NEMO_Asco
XENO_XbJC
CNID_Hvul
CNID_Atet
CNID_Csow
CNID_Smel
CNID_Phyd
CNID_Nvec
CNID_Adig
CNID_Gven
PLAC_Tadh
PORI_Cvar
PORI_Cele
PORI_Psub
PORI_Slac
PORI_Pfic
PORI_Aque
PORI_Ifas
PORI_Cnuc
PORI_Scoa
PORI_Scil
PORI_Ocar
PORI_Ccan
CTEN_Pbac
CTEN_Baby
CTEN_Mlei
CTEN_Vmul
CTEN_Edun
OUTC_Sros
OUTC_Mbre
0.4

S10

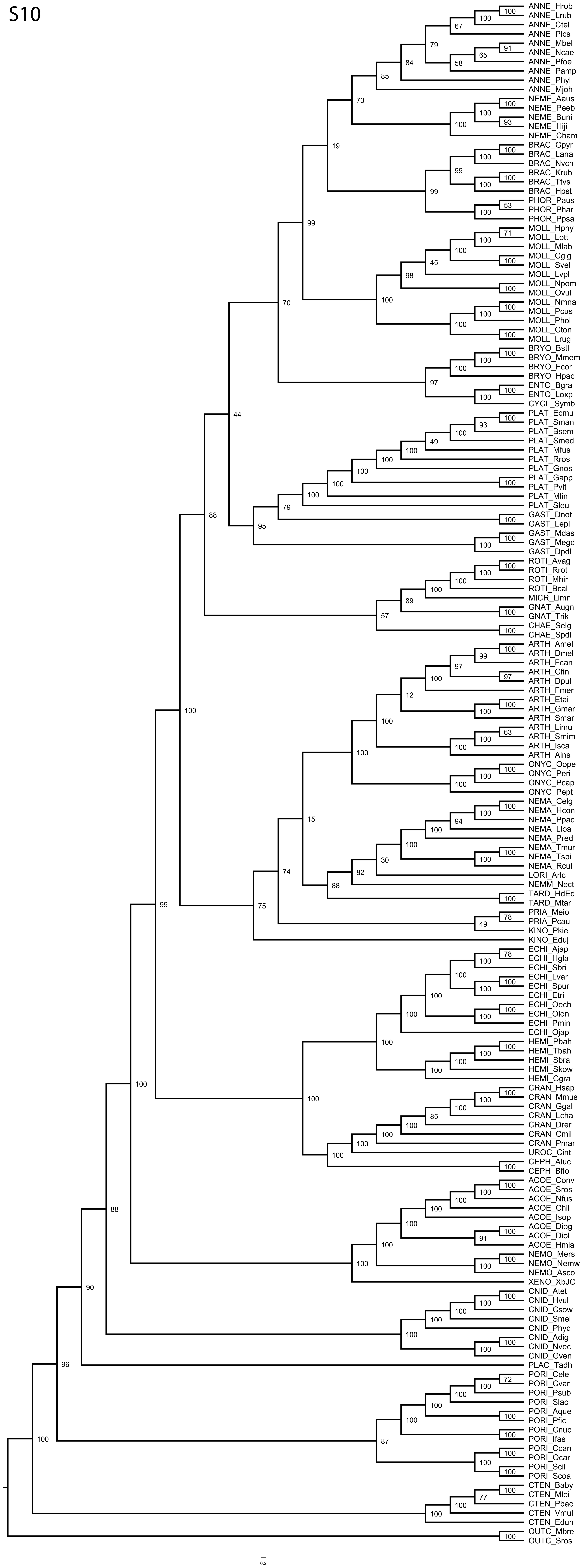
ANNE_Hrob
ANNE_Lrub
ANNE_Ctel
ANNE_Plcs
ANNE_Mbel
ANNE_Ncae
ANNE_Pfoe
ANNE_Pamp
ANNE_Phyl
ANNE_Mjoh
NEME_Aaus
NEME_Peeb
NEME_Buni
NEME_Hiji
NEME_Cham
BRAC_Gpyr
BRAC_Lana
BRAC_Nvcn
BRAC_Krub
BRAC_Ttvs
BRAC_Hpst
PHOR_Paus
PHOR_Phar
PHOR_Ppsa
MOLL_Hphy
MOLL_Lott
MOLL_Mlab
MOLL_Cgig
MOLL_Svel
MOLL_Lvpl
MOLL_Npom
MOLL_Ovul
MOLL_Nmna
MOLL_Pcus
MOLL_Phol
MOLL_Cton
MOLL_Lrug
BRYO_Bstl
BRYO_Mmem
BRYO_Fcor
BRYO_Hpac
ENTO_Bgra
ENTO_Loxp
CYCL_Symb
PLAT_Ecmu
PLAT_Sman
PLAT_Bsem
PLAT_Smed
PLAT_Mfus
PLAT_Rros
PLAT_Gnos
PLAT_Gapp
PLAT_Pvit
PLAT_Mlin
PLAT_Sleu
GAST_Dnot
GAST_Lepi
GAST_Mdas
GAST_Megd
GAST_Dpdl
ROTI_Avag
ROTI_Rrot
ROTI_Mhir
ROTI_Bcal
MICR_Limn
GNAT_Augn
GNAT_Trik
CHAE_Selg
CHAE_Spdl
ARTH_Amel
ARTH_Dmel
ARTH_Fcan
ARTH_Cfin
ARTH_Dpul
ARTH_Fmer
ARTH_Etai
ARTH_Gmar
ARTH_Smar
ARTH_Limu
ARTH_Smim
ARTH_Isca
ARTH_Ains
ONYC_Oope
ONYC_Peri
ONYC_Pcap
ONYC_Pept
NEMA_Celg
NEMA_Hcon
NEMA_Ppac
NEMA_Lloa
NEMA_Pred
NEMA_Tmur
NEMA_Tspi
NEMA_Rcul
LORI_Arlc
NEMM_Nect
TARD_HdEd
TARD_Mtar
PRIA_Meio
PRIA_Pcau
KINO_Pkie
KINO_Eduj
ECHI_Ajap
ECHI_Hgla
ECHI_Sbri
ECHI_Lvar
ECHI_Spur
ECHI_Etri
ECHI_Oech
ECHI_Olon
ECHI_Pmin
ECHI_Ojap
HEMI_Pbah
HEMI_Tbah
HEMI_Sbra
HEMI_Skow
HEMI_Cgra
CRAN_Hsap
CRAN_Mmus
CRAN_Ggal
CRAN_Lcha
CRAN_Drer
CRAN_Cmil
CRAN_Pmar
UROC_Cint
CEPH_Aluc
CEPH_Bflo
ACOE_Conv
ACOE_Sros
ACOE_Nfus
ACOE_Chil
ACOE_Isop
ACOE_Diog
ACOE_Diol
ACOE_Hmia
NEMO_Mers
NEMO_Nemw
NEMO_Asco
XENO_XbJC
CNID_Atet
CNID_Hvul
CNID_Csow
CNID_Smel
CNID_Phyd
CNID_Adig
CNID_Nvec
CNID_Gven
PLAC_Tadh
PORI_Cele
PORI_Cvar
PORI_Psub
PORI_Slac
PORI_Aque
PORI_Pfic
PORI_Cnuc
PORI_Ifas
PORI_Ccan
PORI_Ocar
PORI_Scil
PORI_Scoa
CTEN_Baby
CTEN_Mlei
CTEN_Pbac
CTEN_Vmul
CTEN_Edun
OUTC_Mbre
OUTC_Sros
0.2

S11

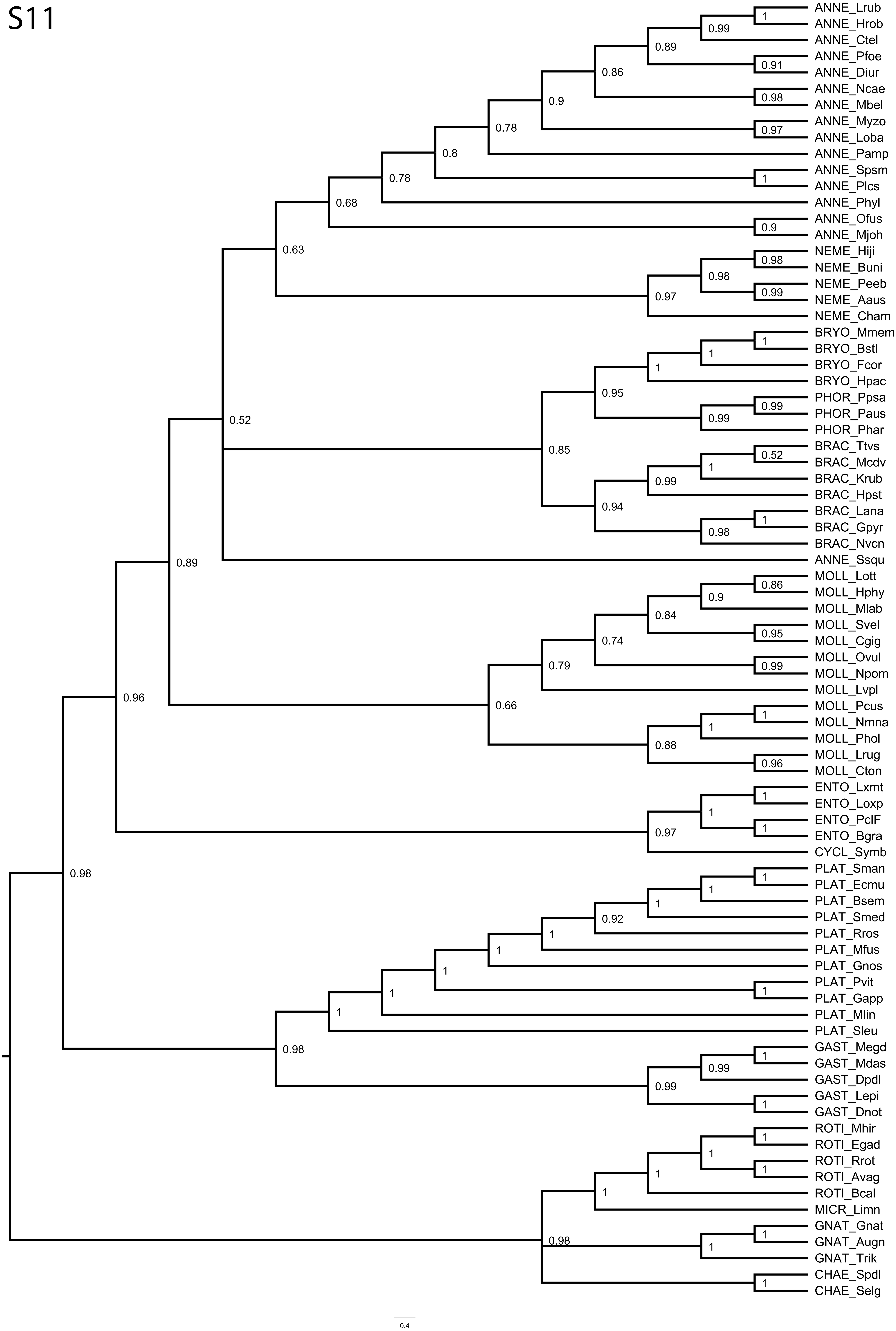
ANNE_Lrub
ANNE_Hrob
ANNE_Ctel
ANNE_Pfoe
ANNE_Diur
ANNE_Ncae
ANNE_Mbel
ANNE_Myzo
ANNE_Loba
ANNE_Pamp
ANNE_Spsm
ANNE_Plcs
ANNE_Phyl
ANNE_Ofus
ANNE_Mjoh
NEME_Hiji
NEME_Buni
NEME_Peeb
NEME_Aaus
NEME_Cham
BRYO_Mmem
BRYO_Bstl
BRYO_Fcor
BRYO_Hpac
PHOR_Ppsa
PHOR_Paus
PHOR_Phar
BRAC_Ttvs
BRAC_Mcdv
BRAC_Krub
BRAC_Hpst
BRAC_Lana
BRAC_Gpyr
BRAC_Nvcn
ANNE_Ssqu
MOLL_Lott
MOLL_Hphy
MOLL_Mlab
MOLL_Svel
MOLL_Cgig
MOLL_Ovul
MOLL_Npom
MOLL_Lvpl
MOLL_Pcus
MOLL_Nmna
MOLL_Phol
MOLL_Lrug
MOLL_Cton
ENTO_Lxmt
ENTO_Loxp
ENTO_PclF
ENTO_Bgra
CYCL_Symb
PLAT_Sman
PLAT_Ecmu
PLAT_Bsem
PLAT_Smed
PLAT_Rros
PLAT_Mfus
PLAT_Gnos
PLAT_Pvit
PLAT_Gapp
PLAT_Mlin
PLAT_Sleu
GAST_Megd
GAST_Mdas
GAST_Dpdl
GAST_Lepi
GAST_Dnot
ROTI_Mhir
ROTI_Egad
ROTI_Rrot
ROTI_Avag
ROTI_Bcal
MICR_Limn
GNAT_Gnat
GNAT_Augn
GNAT_Trik
CHAE_Spdl
CHAE_Selg
0.4

S12

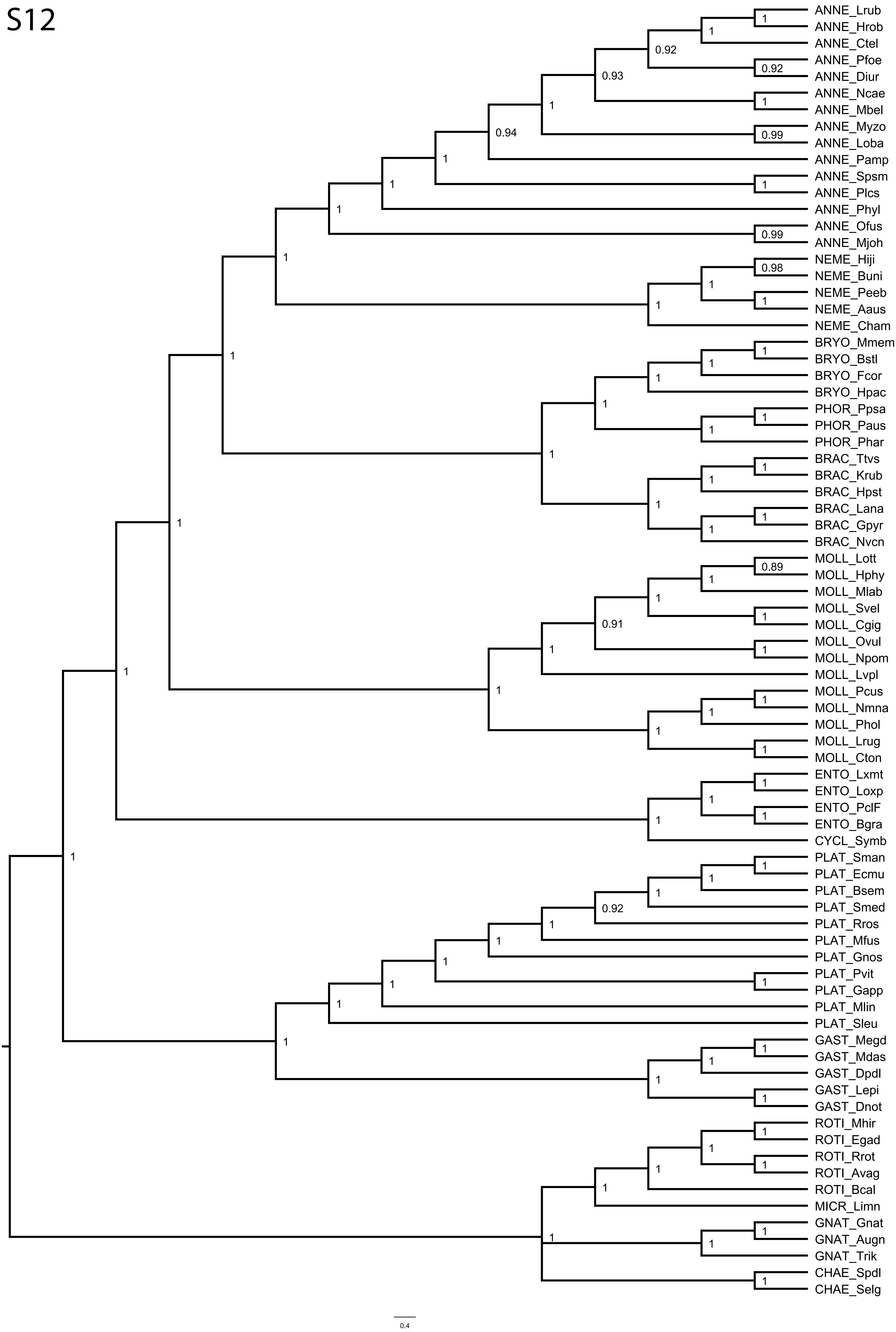
ANNE_Lrub
ANNE_Hrob
ANNE_Ctel
ANNE_Pfoe
ANNE_Diur
ANNE_Ncae
ANNE_Mbel
ANNE_Myzo
ANNE_Loba
ANNE_Pamp
ANNE_Spsm
ANNE_Plcs
ANNE_Phyl
ANNE_Ofus
ANNE_Mjoh
NEME_Hiji
NEME_Buni
NEME_Peeb
NEME_Aaus
NEME_Cham
BRYO_Mmem
BRYO_Bstl
BRYO_Fcor
BRYO_Hpac
PHOR_Ppsa
PHOR_Paus
PHOR_Phar
BRAC_Ttvs
BRAC_Krub
BRAC_Hpst
BRAC_Lana
BRAC_Gpyr
BRAC_Nvcn
MOLL_Lott
MOLL_Hphy
MOLL_Mlab
MOLL_Svel
MOLL_Cgig
MOLL_Ovul
MOLL_Npom
MOLL_Lvpl
MOLL_Pcus
MOLL_Nmna
MOLL_Phol
MOLL_Lrug
MOLL_Cton
ENTO_Lxmt
ENTO_Loxp
ENTO_PclF
ENTO_Bgra
CYCL_Symb
PLAT_Sman
PLAT_Ecmu
PLAT_Bsem
PLAT_Smed
PLAT_Rros
PLAT_Mfus
PLAT_Gnos
PLAT_Pvit
PLAT_Gapp
PLAT_Mlin
PLAT_Sleu
GAST_Megd
GAST_Mdas
GAST_Dpdl
GAST_Lepi
GAST_Dnot
ROTI_Mhir
ROTI_Egad
ROTI_Rrot
ROTI_Avag
ROTI_Bcal
MICR_Limn
GNAT_Gnat
GNAT_Augn
GNAT_Trik
CHAE_Spdl
CHAE_Selg
0.92
0.93
0.94
0.99
0.98
0.89
0.91
1
0.4

S13

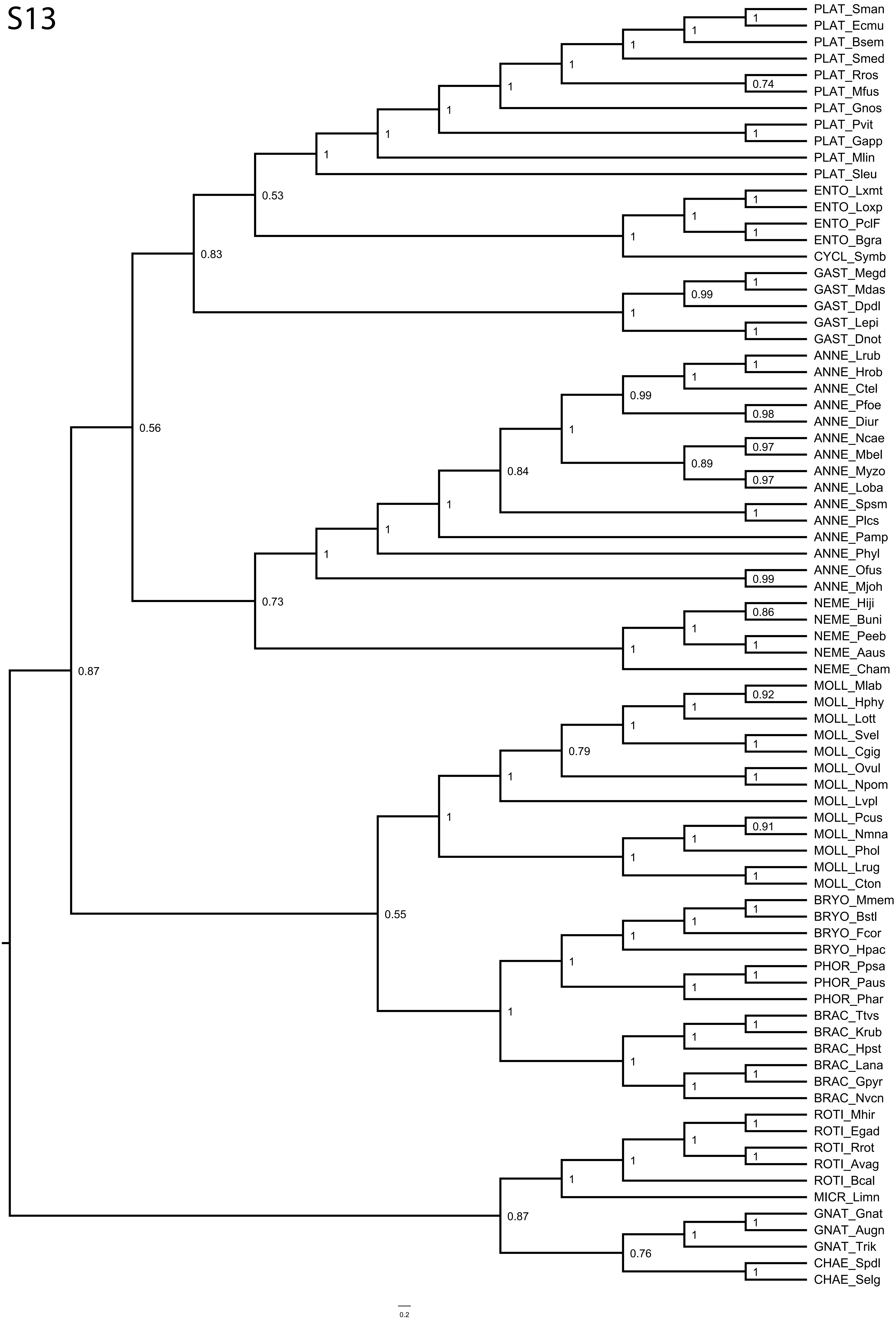
PLAT_Sman
PLAT_Ecmu
PLAT_Bsem
PLAT_Smed
PLAT_Rros
PLAT_Mfus
PLAT_Gnos
PLAT_Pvit
PLAT_Gapp
PLAT_Mlin
PLAT_Sleu
ENTO_Lxmt
ENTO_Loxp
ENTO_PclF
ENTO_Bgra
CYCL_Symb
GAST_Megd
GAST_Mdas
GAST_Dpdl
GAST_Lepi
GAST_Dnot
ANNE_Lrub
ANNE_Hrob
ANNE_Ctel
ANNE_Pfoe
ANNE_Diur
ANNE_Ncae
ANNE_Mbel
ANNE_Myzo
ANNE_Loba
ANNE_Spsm
ANNE_Plcs
ANNE_Pamp
ANNE_Phyl
ANNE_Ofus
ANNE_Mjoh
NEME_Hiji
NEME_Buni
NEME_Peeb
NEME_Aaus
NEME_Cham
MOLL_Mlab
MOLL_Hphy
MOLL_Lott
MOLL_Svel
MOLL_Cgig
MOLL_Ovul
MOLL_Npom
MOLL_Lvpl
MOLL_Pcus
MOLL_Nmna
MOLL_Phol
MOLL_Lrug
MOLL_Cton
BRYO_Mmem
BRYO_Bstl
BRYO_Fcor
BRYO_Hpac
PHOR_Ppsa
PHOR_Paus
PHOR_Phar
BRAC_Ttvs
BRAC_Krub
BRAC_Hpst
BRAC_Lana
BRAC_Gpyr
BRAC_Nvcn
ROTI_Mhir
ROTI_Egad
ROTI_Rrot
ROTI_Avag
ROTI_Bcal
MICR_Limn
GNAT_Gnat
GNAT_Augn
GNAT_Trik
CHAE_Spdl
CHAE_Selg
0.53
0.83
0.56
0.74
0.99
0.99
0.98
0.97
0.89
0.97
0.84
0.99
0.73
0.86
0.87
0.92
0.79
0.91
0.55
0.87
0.76
0.2

S14

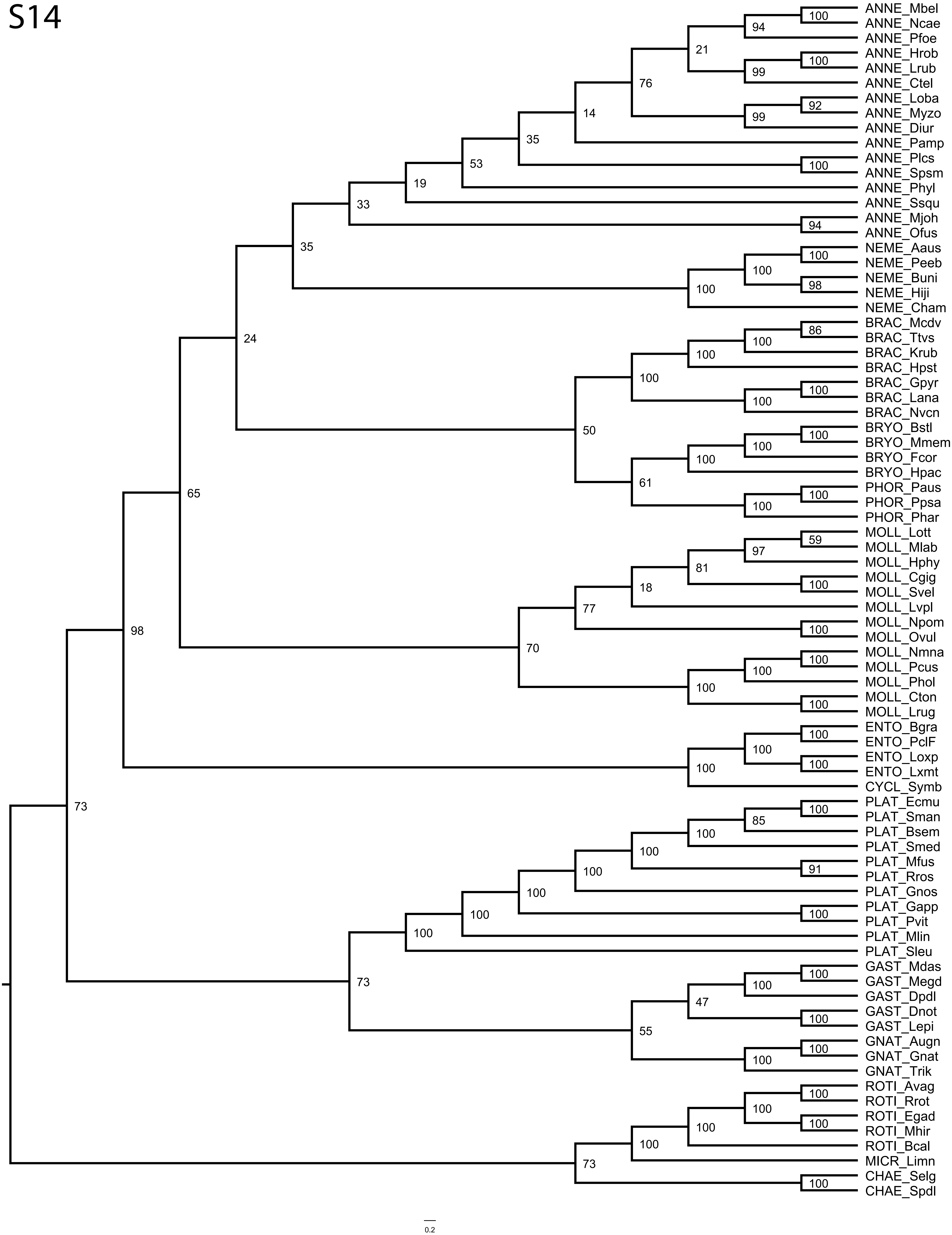
ANNE_Mbel
ANNE_Ncae
ANNE_Pfoe
ANNE_Hrob
ANNE_Lrub
ANNE_Ctel
ANNE_Loba
ANNE_Myzo
ANNE_Diur
ANNE_Pamp
ANNE_Plcs
ANNE_Spsm
ANNE_Phyl
ANNE_Ssqu
ANNE_Mjoh
ANNE_Ofus
NEME_Aaus
NEME_Peeb
NEME_Buni
NEME_Hiji
NEME_Cham
BRAC_Mcdv
BRAC_Ttvs
BRAC_Krub
BRAC_Hpst
BRAC_Gpyr
BRAC_Lana
BRAC_Nvcn
BRYO_Bstl
BRYO_Mmem
BRYO_Fcor
BRYO_Hpac
PHOR_Paus
PHOR_Ppsa
PHOR_Phar
MOLL_Lott
MOLL_Mlab
MOLL_Hphy
MOLL_Cgig
MOLL_Svel
MOLL_Lvpl
MOLL_Npom
MOLL_Ovul
MOLL_Nmna
MOLL_Pcus
MOLL_Phol
MOLL_Cton
MOLL_Lrug
ENTO_Bgra
ENTO_PclF
ENTO_Loxp
ENTO_Lxmt
CYCL_Symb
PLAT_Ecmu
PLAT_Sman
PLAT_Bsem
PLAT_Smed
PLAT_Mfus
PLAT_Rros
PLAT_Gnos
PLAT_Gapp
PLAT_Pvit
PLAT_Mlin
PLAT_Sleu
GAST_Mdas
GAST_Megd
GAST_Dpdl
GAST_Dnot
GAST_Lepi
GNAT_Augn
GNAT_Gnat
GNAT_Trik
ROTI_Avag
ROTI_Rrot
ROTI_Egad
ROTI_Mhir
ROTI_Bcal
MICR_Limn
CHAE_Selg
CHAE_Spdl
0.2

S15

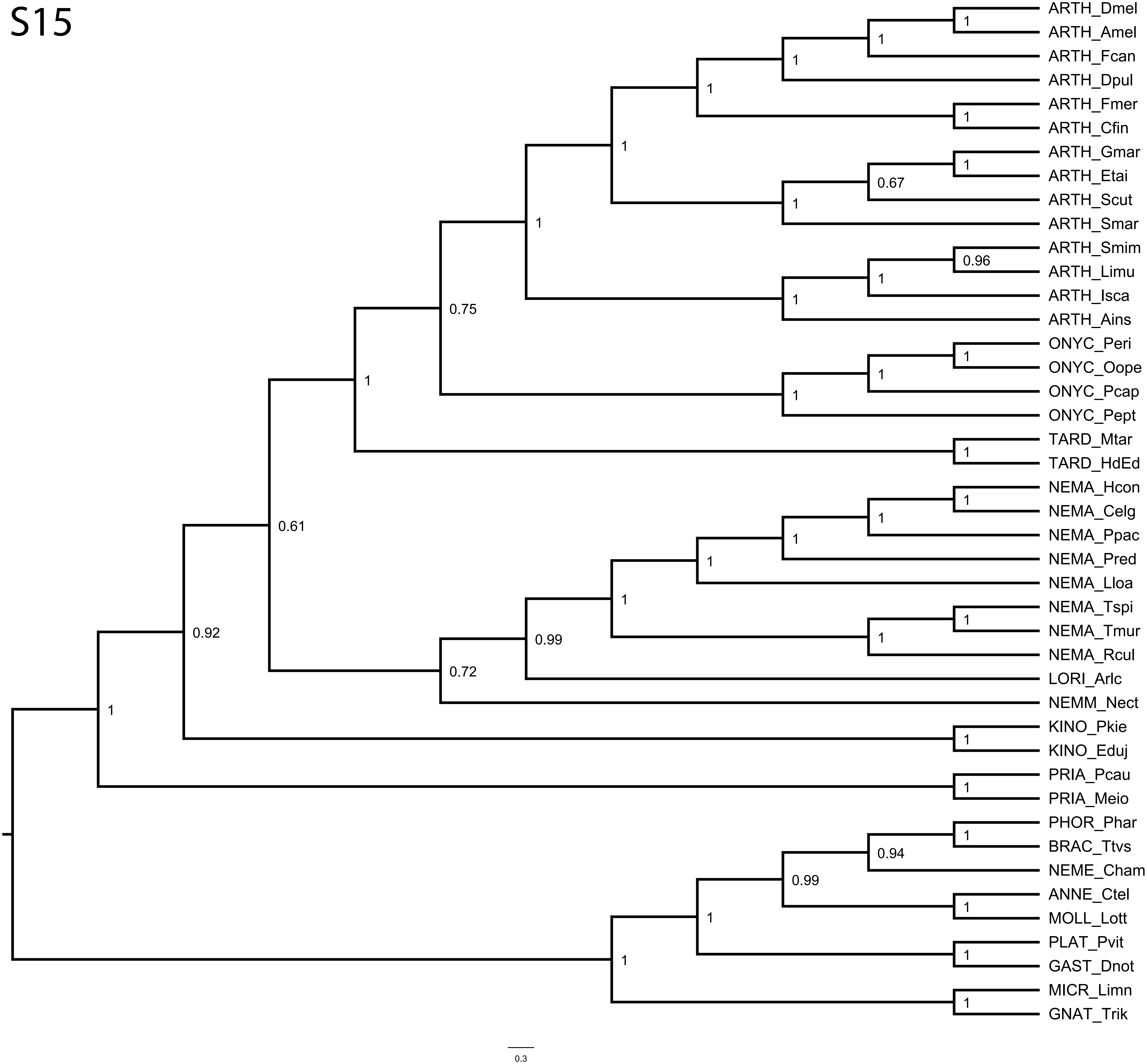
ARTH_Dmel
ARTH_Amel
ARTH_Fcan
ARTH_Dpul
ARTH_Fmer
ARTH_Cfin
ARTH_Gmar
ARTH_Etai
ARTH_Scut
ARTH_Smar
ARTH_Smim
ARTH_Limu
ARTH_Isca
ARTH_Ains
ONYC_Peri
ONYC_Oope
ONYC_Pcap
ONYC_Pept
TARD_Mtar
TARD_HdEd
NEMA_Hcon
NEMA_Celg
NEMA_Ppac
NEMA_Pred
NEMA_Lloa
NEMA_Tspi
NEMA_Tmur
NEMA_Rcul
LORI_Arlc
NEMM_Nect
KINO_Pkie
KINO_Eduj
PRIA_Pcau
PRIA_Meio
PHOR_Phar
BRAC_Ttvs
NEME_Cham
ANNE_Ctel
MOLL_Lott
PLAT_Pvit
GAST_Dnot
MICR_Limn
GNAT_Trik
0.67
0.96
0.75
0.61
0.92
0.99
0.72
0.94
0.99
0.3

S16

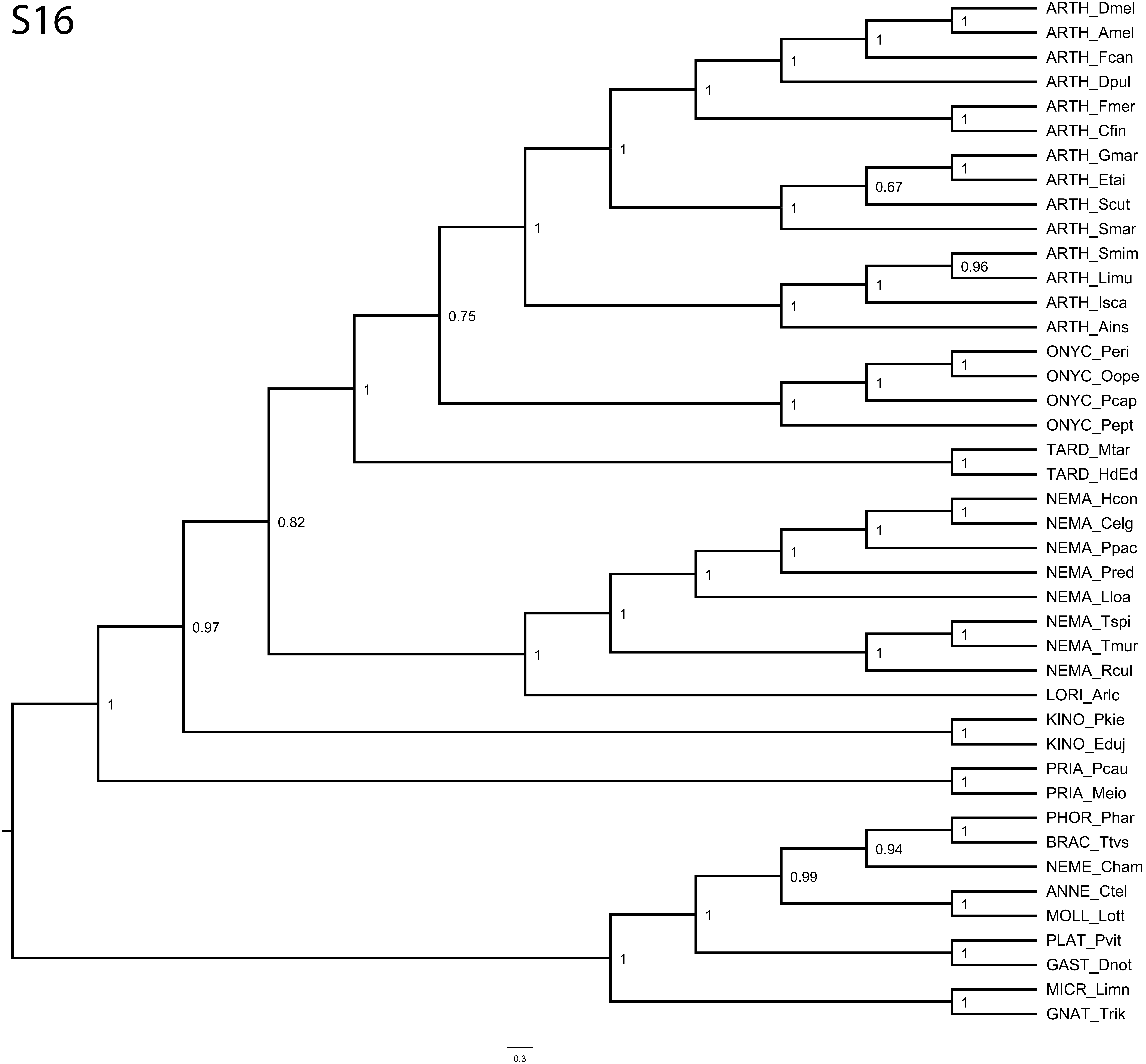
ARTH_Dmel
ARTH_Amel
ARTH_Fcan
ARTH_Dpul
ARTH_Fmer
ARTH_Cfin
ARTH_Gmar
ARTH_Etai
ARTH_Scut
ARTH_Smar
ARTH_Smim
ARTH_Limu
ARTH_Isca
ARTH_Ains
ONYC_Peri
ONYC_Oope
ONYC_Pcap
ONYC_Pept
TARD_Mtar
TARD_HdEd
NEMA_Hcon
NEMA_Celg
NEMA_Ppac
NEMA_Pred
NEMA_Lloa
NEMA_Tspi
NEMA_Tmur
NEMA_Rcul
LORI_Arlc
KINO_Pkie
KINO_Eduj
PRIA_Pcau
PRIA_Meio
PHOR_Phar
BRAC_Ttvs
NEME_Cham
ANNE_Ctel
MOLL_Lott
PLAT_Pvit
GAST_Dnot
MICR_Limn
GNAT_Trik
0.67
0.96
0.75
0.82
0.97
0.94
0.99
0.3

S17

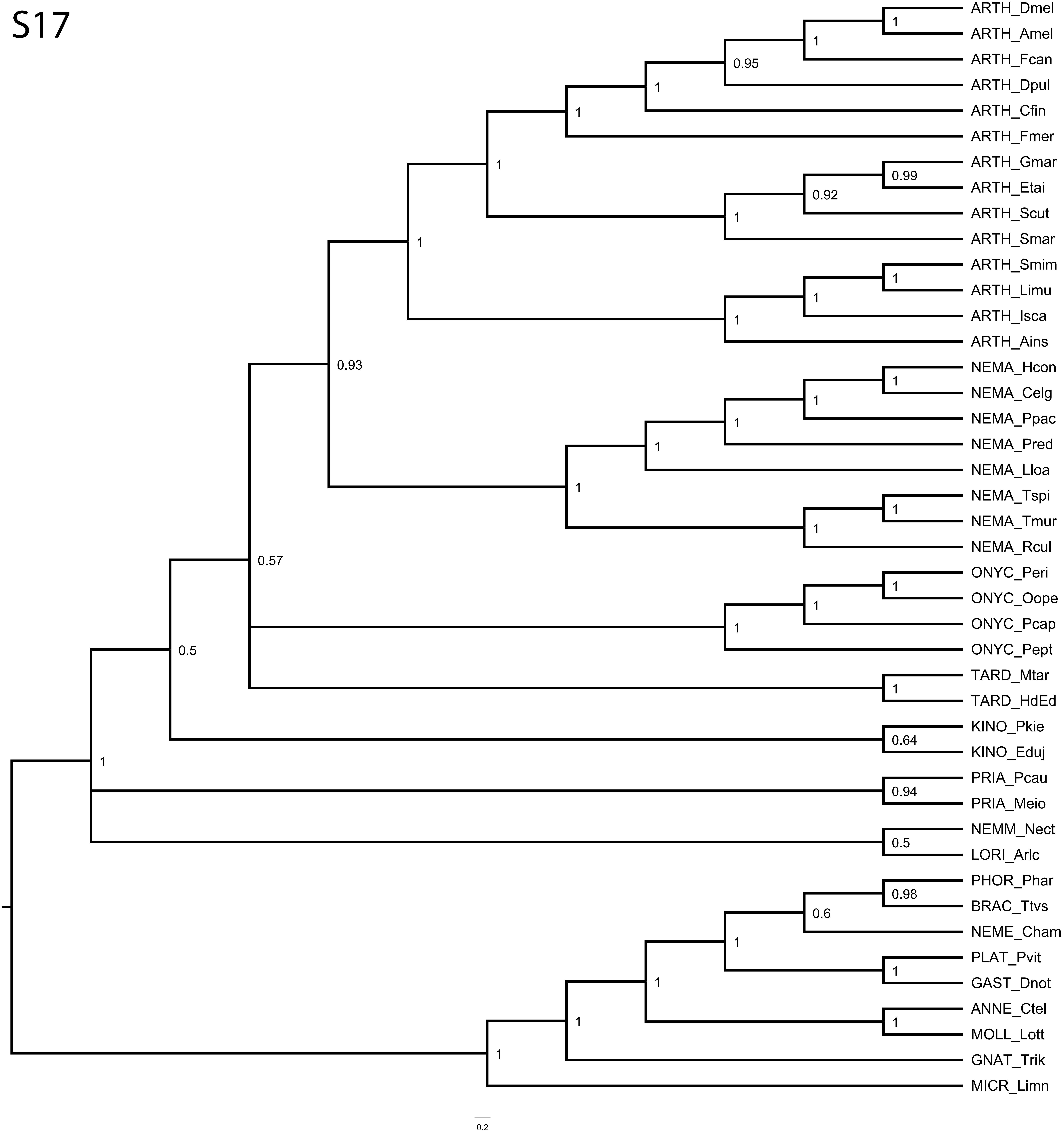
ARTH_Dmel
ARTH_Amel
ARTH_Fcan
ARTH_Dpul
ARTH_Cfin
ARTH_Fmer
ARTH_Gmar
ARTH_Etai
ARTH_Scut
ARTH_Smar
ARTH_Smim
ARTH_Limu
ARTH_Isca
ARTH_Ains
NEMA_Hcon
NEMA_Celg
NEMA_Ppac
NEMA_Pred
NEMA_Lloa
NEMA_Tspi
NEMA_Tmur
NEMA_Rcul
ONYC_Peri
ONYC_Oope
ONYC_Pcap
ONYC_Pept
TARD_Mtar
TARD_HdEd
KINO_Pkie
KINO_Eduj
PRIA_Pcau
PRIA_Meio
NEMM_Nect
LORI_Arlc
PHOR_Phar
BRAC_Ttvs
NEME_Cham
PLAT_Pvit
GAST_Dnot
ANNE_Ctel
MOLL_Lott
GNAT_Trik
MICR_Limn
1
1
0.95
1
1
0.99
0.92
1
1
1
1
1
1
0.93
1
1
1
1
1
1
0.57
1
1
1
1
0.5
0.64
1
0.94
0.5
0.98
0.6
1
1
1
1
1
1
0.2

S18

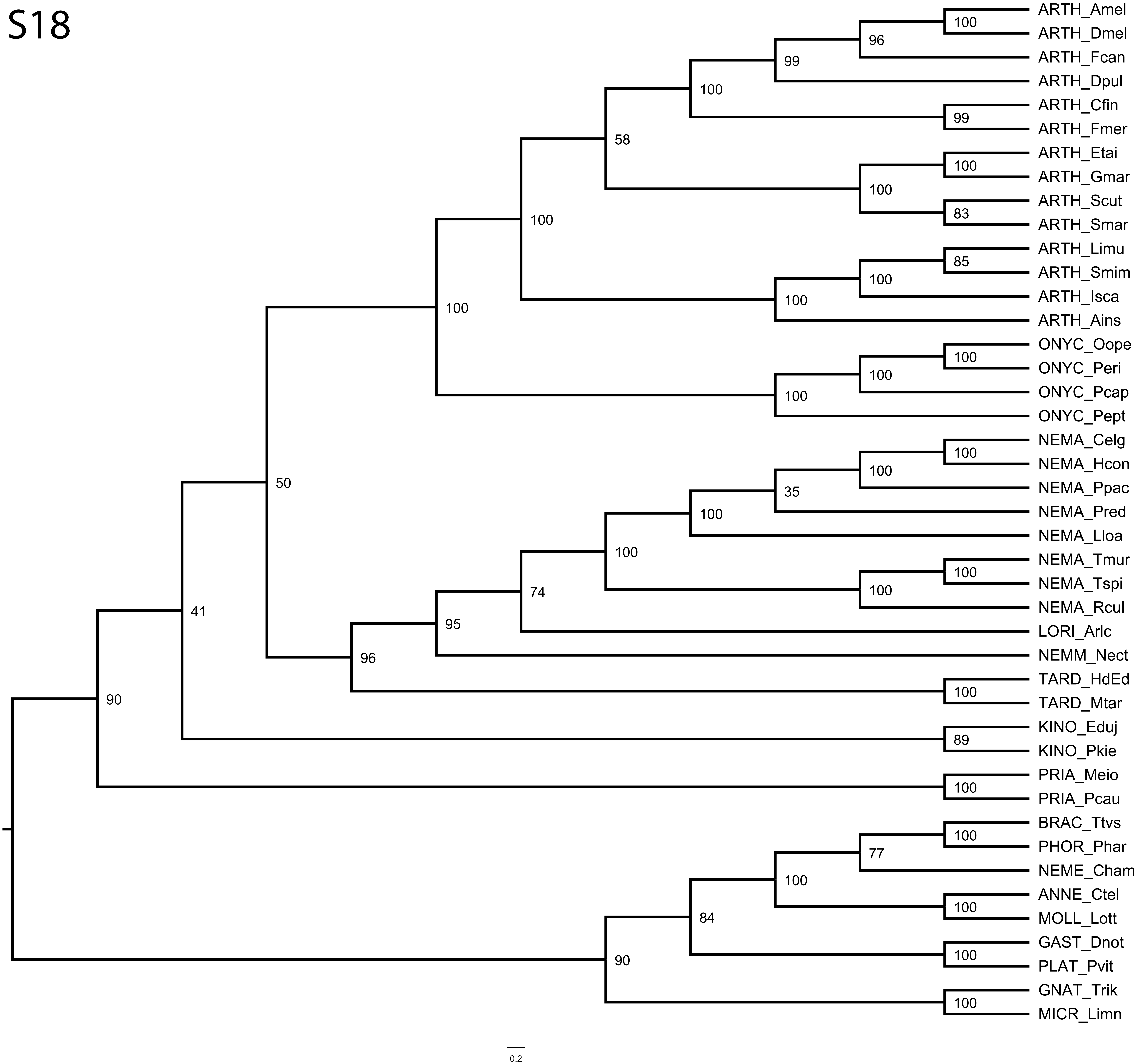
ARTH_Amel
ARTH_Dmel
ARTH_Fcan
ARTH_Dpul
ARTH_Cfin
ARTH_Fmer
ARTH_Etai
ARTH_Gmar
ARTH_Scut
ARTH_Smar
ARTH_Limu
ARTH_Smim
ARTH_Isca
ARTH_Ains
ONYC_Oope
ONYC_Peri
ONYC_Pcap
ONYC_Pept
NEMA_Celg
NEMA_Hcon
NEMA_Ppac
NEMA_Pred
NEMA_Lloa
NEMA_Tmur
NEMA_Tspi
NEMA_Rcul
LORI_Arlc
NEMM_Nect
TARD_HdEd
TARD_Mtar
KINO_Eduj
KINO_Pkie
PRIA_Meio
PRIA_Pcau
BRAC_Ttvs
PHOR_Phar
NEME_Cham
ANNE_Ctel
MOLL_Lott
GAST_Dnot
PLAT_Pvit
GNAT_Trik
MICR_Limn
0.2

S19

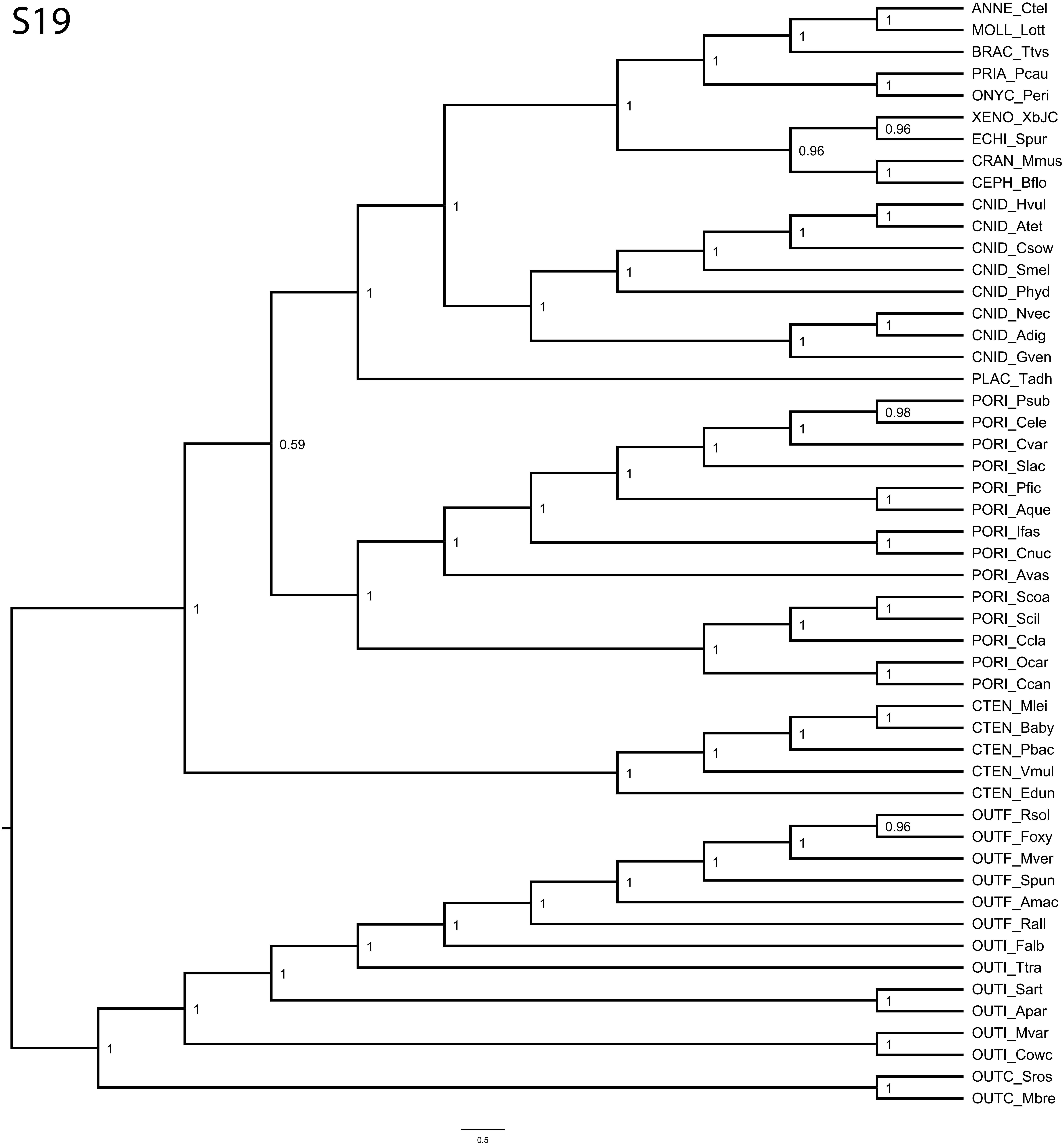
ANNE_Ctel
MOLL_Lott
BRAC_Ttvs
PRIA_Pcau
ONYC_Peri
XENO_XbJC
ECHI_Spur
CRAN_Mmus
CEPH_Bflo
CNID_Hvul
CNID_Atet
CNID_Csow
CNID_Smel
CNID_Phyd
CNID_Nvec
CNID_Adig
CNID_Gven
PLAC_Tadh
PORI_Psub
PORI_Cele
PORI_Cvar
PORI_Slac
PORI_Pfic
PORI_Aque
PORI_Ifas
PORI_Cnuc
PORI_Avas
PORI_Scoa
PORI_Scil
PORI_Ccla
PORI_Ocar
PORI_Ccan
CTEN_Mlei
CTEN_Baby
CTEN_Pbac
CTEN_Vmul
CTEN_Edun
OUTF_Rsol
OUTF_Foxy
OUTF_Mver
OUTF_Spun
OUTF_Amac
OUTF_Rall
OUTI_Falb
OUTI_Ttra
OUTI_Sart
OUTI_Apar
OUTI_Mvar
OUTI_Cowc
OUTC_Sros
OUTC_Mbre
0.96
0.96
0.59
0.98
0.96
0.5

S20

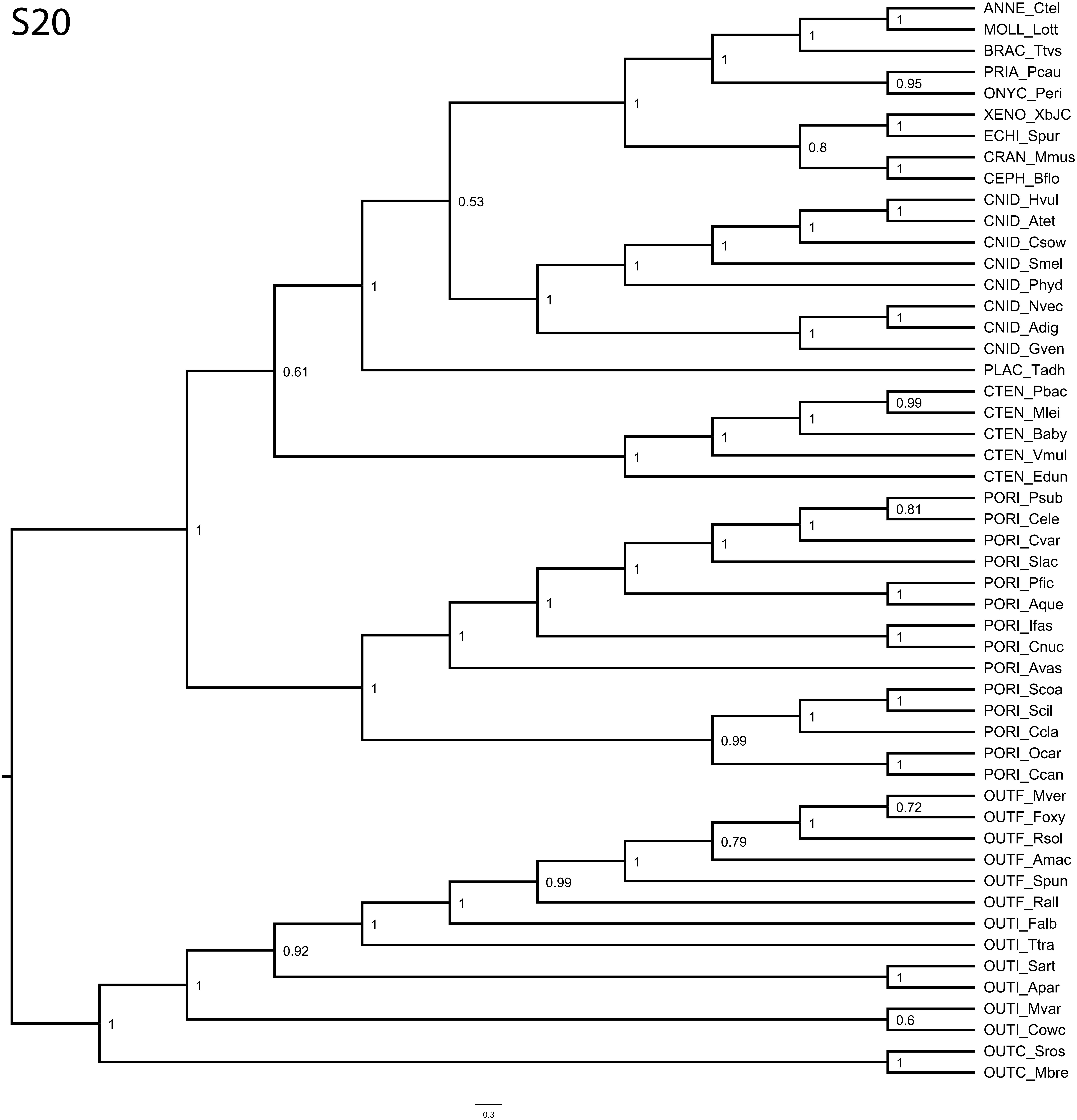
ANNE_Ctel
MOLL_Lott
BRAC_Ttvs
PRIA_Pcau
ONYC_Peri
XENO_XbJC
ECHI_Spur
CRAN_Mmus
CEPH_Bflo
CNID_Hvul
CNID_Atet
CNID_Csow
CNID_Smel
CNID_Phyd
CNID_Nvec
CNID_Adig
CNID_Gven
PLAC_Tadh
CTEN_Pbac
CTEN_Mlei
CTEN_Baby
CTEN_Vmul
CTEN_Edun
PORI_Psub
PORI_Cele
PORI_Cvar
PORI_Slac
PORI_Pfic
PORI_Aque
PORI_Ifas
PORI_Cnuc
PORI_Avas
PORI_Scoa
PORI_Scil
PORI_Ccla
PORI_Ocar
PORI_Ccan
OUTF_Mver
OUTF_Foxy
OUTF_Rsol
OUTF_Amac
OUTF_Spun
OUTF_Rall
OUTI_Falb
OUTI_Ttra
OUTI_Sart
OUTI_Apar
OUTI_Mvar
OUTI_Cowc
OUTC_Sros
OUTC_Mbre
0.95
0.8
0.53
0.61
0.99
0.81
0.99
0.72
0.79
0.99
0.92
0.6
0.3

S21

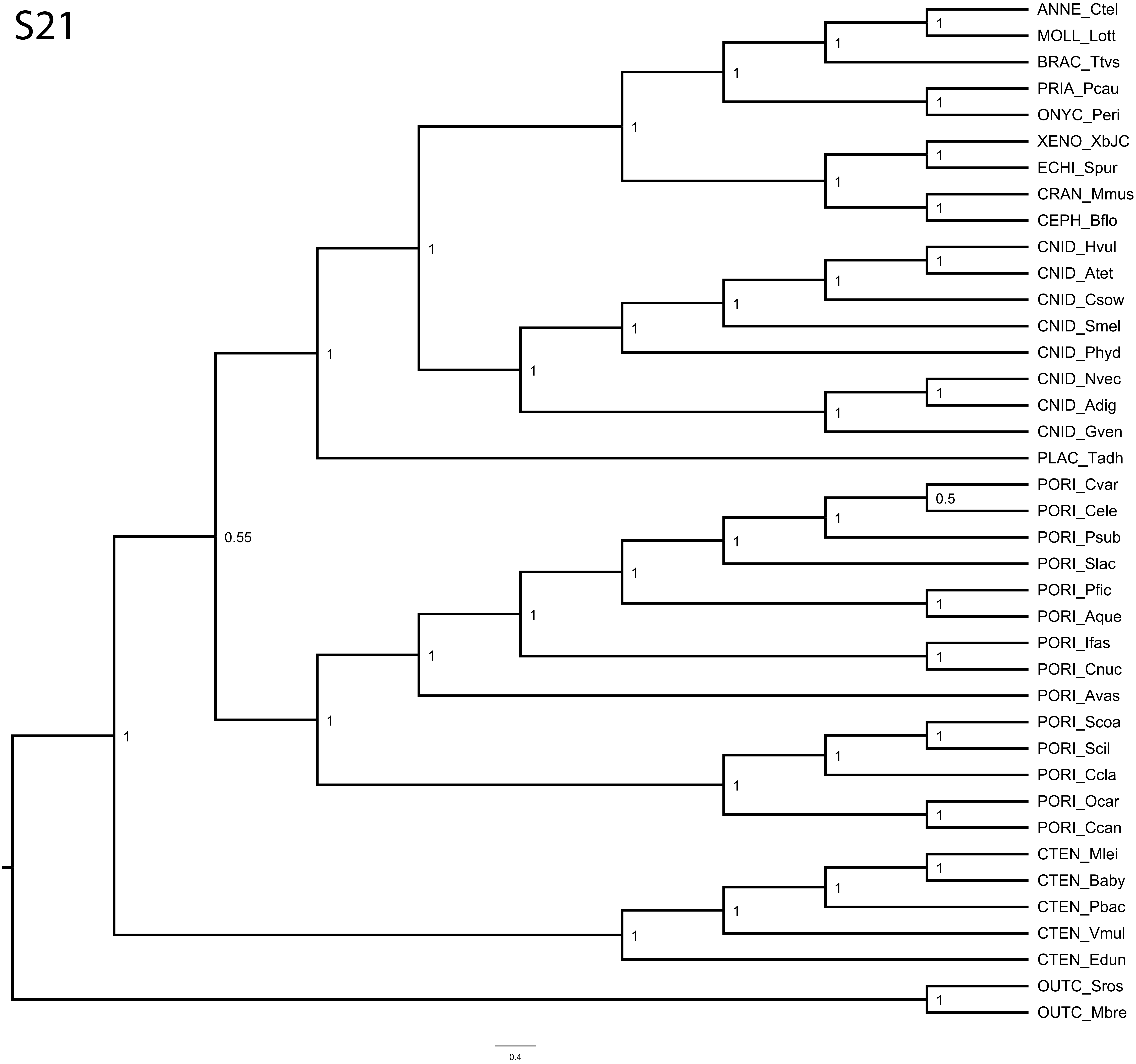
ANNE_Ctel
MOLL_Lott
BRAC_Ttvs
PRIA_Pcau
ONYC_Peri
XENO_XbJC
ECHI_Spur
CRAN_Mmus
CEPH_Bflo
CNID_Hvul
CNID_Atet
CNID_Csow
CNID_Smel
CNID_Phyd
CNID_Nvec
CNID_Adig
CNID_Gven
PLAC_Tadh
PORI_Cvar
PORI_Cele
PORI_Psub
PORI_Slac
PORI_Pfic
PORI_Aque
PORI_Ifas
PORI_Cnuc
PORI_Avas
PORI_Scoa
PORI_Scil
PORI_Ccla
PORI_Ocar
PORI_Ccan
CTEN_Mlei
CTEN_Baby
CTEN_Pbac
CTEN_Vmul
CTEN_Edun
OUTC_Sros
OUTC_Mbre
0.55
0.5
0.4

S22

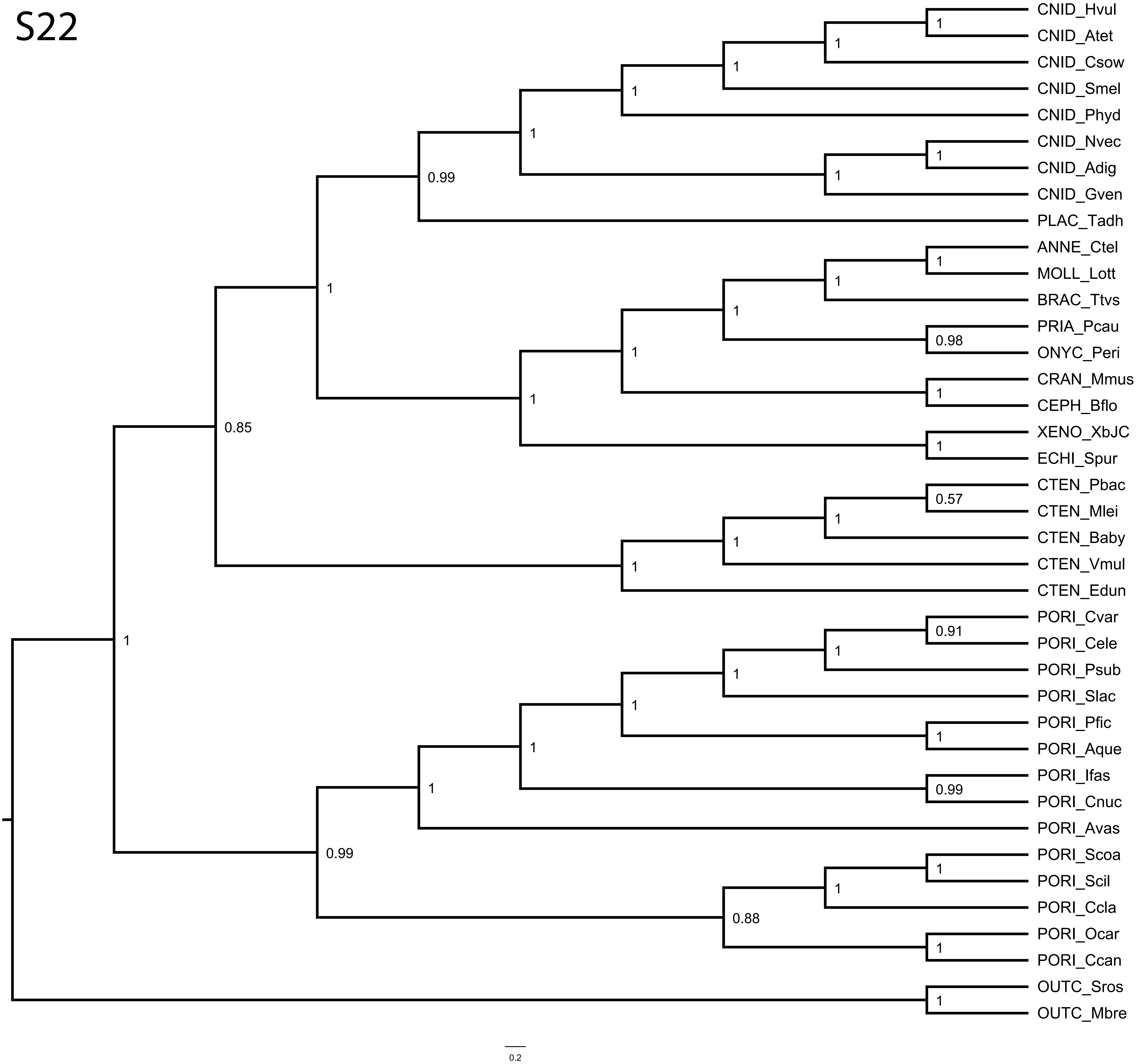
CNID_Hvul
CNID_Atet
CNID_Csow
CNID_Smel
CNID_Phyd
CNID_Nvec
CNID_Adig
CNID_Gven
PLAC_Tadh
ANNE_Ctel
MOLL_Lott
BRAC_Ttvs
PRIA_Pcau
ONYC_Peri
CRAN_Mmus
CEPH_Bflo
XENO_XbJC
ECHI_Spur
CTEN_Pbac
CTEN_Mlei
CTEN_Baby
CTEN_Vmul
CTEN_Edun
PORI_Cvar
PORI_Cele
PORI_Psub
PORI_Slac
PORI_Pfic
PORI_Aque
PORI_Ifas
PORI_Cnuc
PORI_Avas
PORI_Scoa
PORI_Scil
PORI_Ccla
PORI_Ocar
PORI_Ccan
OUTC_Sros
OUTC_Mbre
0.99
0.85
0.98
0.57
0.91
0.99
0.99
0.88
0.2

S23

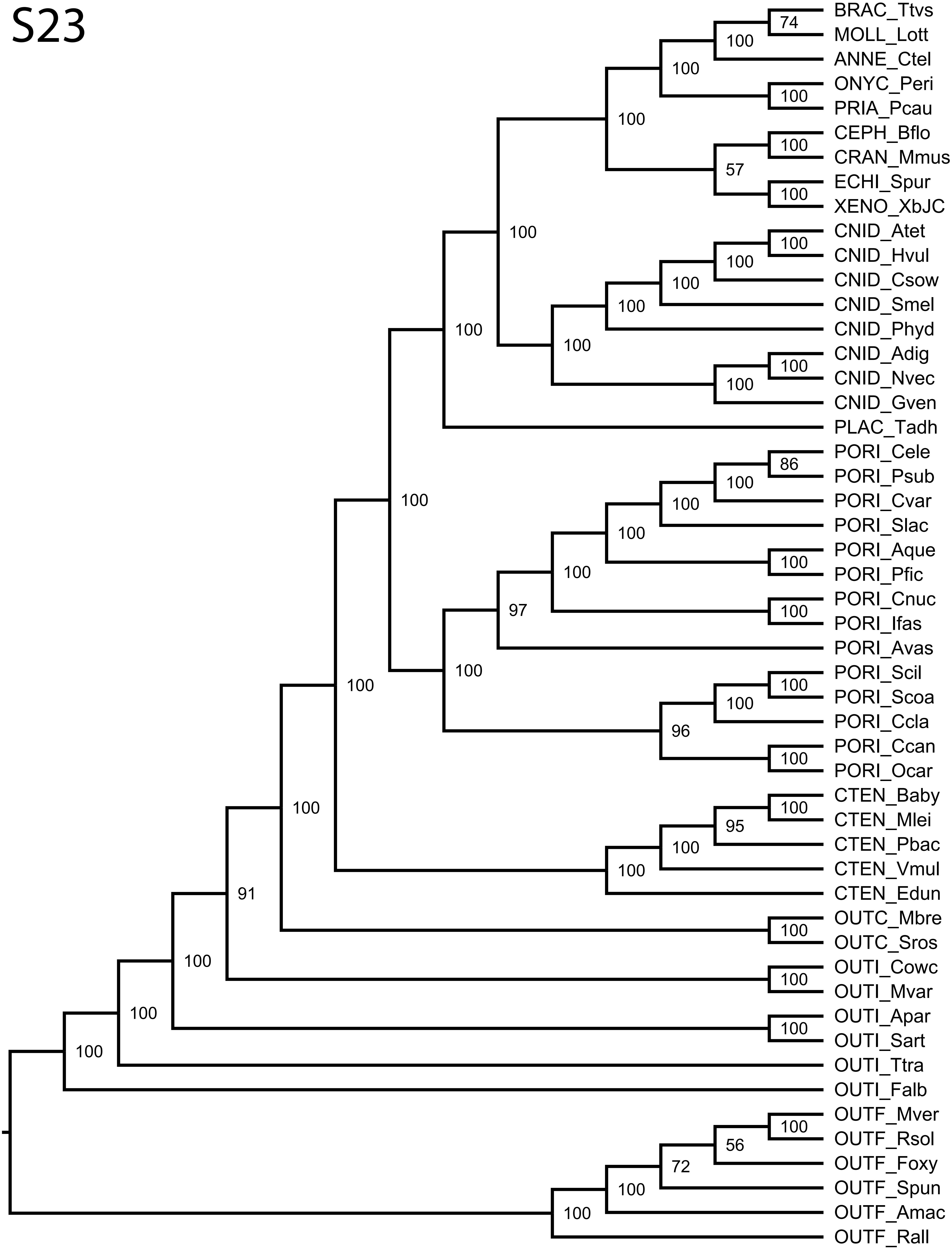

BRAC_Ttvs
MOLL_Lott
ANNE_Ctel
ONYC_Peri
PRIA_Pcau
CEPH_Bflo
CRAN_Mmus
ECHI_Spur
XENO_XbJC
CNID_Atet
CNID_Hvul
CNID_Csow
CNID_Smel
CNID_Phyd
CNID_Adig
CNID_Nvec
CNID_Gven
PLAC_Tadh
PORI_Cele
PORI_Psub
PORI_Cvar
PORI_Slac
PORI_Aque
PORI_Pfic
PORI_Cnuc
PORI_Ifas
PORI_Avas
PORI_Scil
PORI_Scoa
PORI_Ccla
PORI_Ccan
PORI_Ocar
CTEN_Baby
CTEN_Mlei
CTEN_Pbac
CTEN_Vmul
CTEN_Edun
OUTC_Mbre
OUTC_Sros
OUTI_Cowc
OUTI_Mvar
OUTI_Apar
OUTI_Sart
OUTI_Ttra
OUTI_Falb
OUTF_Mver
OUTF_Rsol
OUTF_Foxy
OUTF_Spun
OUTF_Amac
OUTF_Rall

S24

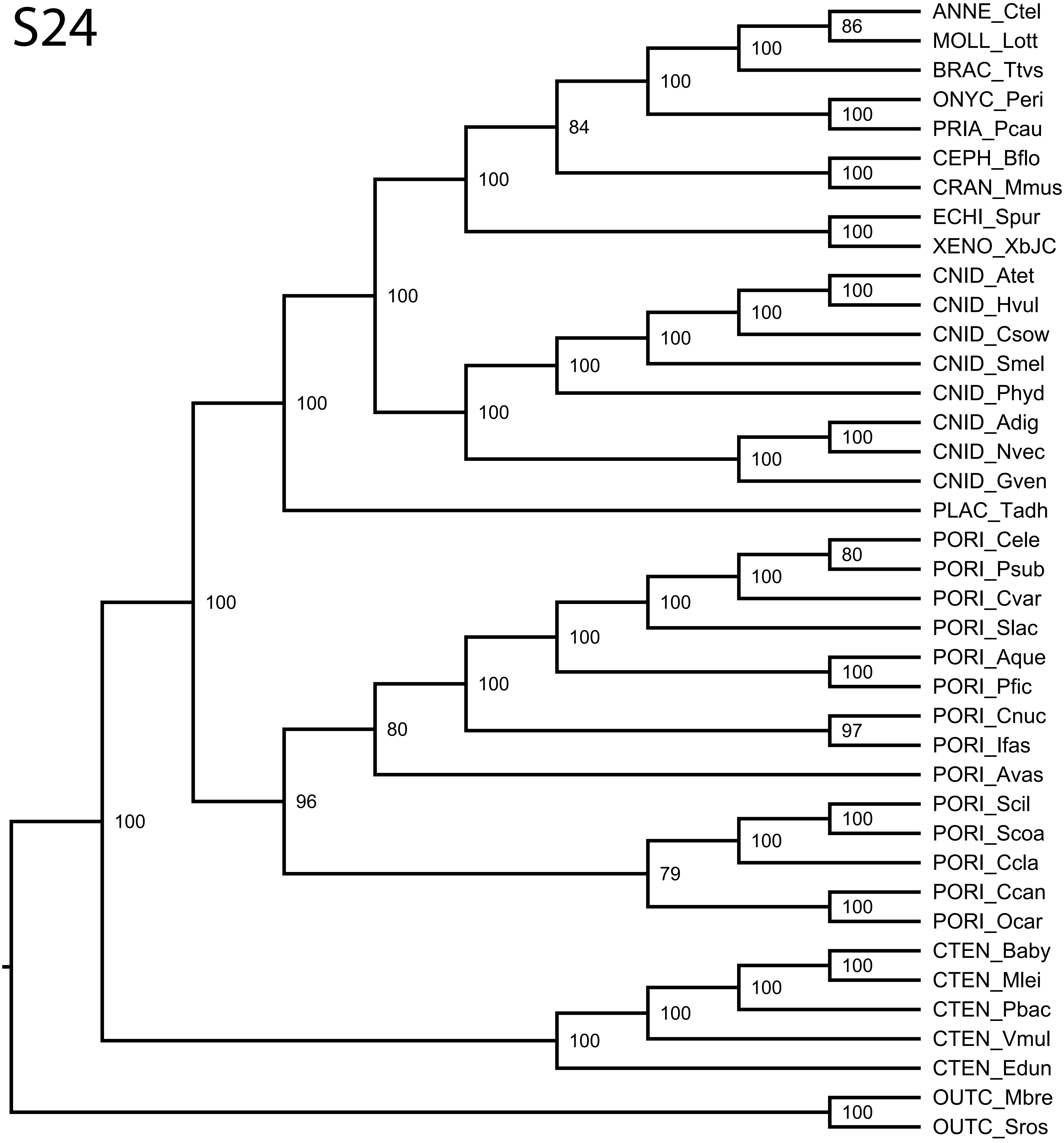
ANNE_Ctel
MOLL_Lott
BRAC_Ttvs
ONYC_Peri
PRIA_Pcau
CEPH_Bflo
CRAN_Mmus
ECHI_Spur
XENO_XbJC
CNID_Atet
CNID_Hvul
CNID_Csow
CNID_Smel
CNID_Phyd
CNID_Adig
CNID_Nvec
CNID_Gven
PLAC_Tadh
PORI_Cele
PORI_Psub
PORI_Cvar
PORI_Slac
PORI_Aque
PORI_Pfic
PORI_Cnuc
PORI_Ifas
PORI_Avas
PORI_Scil
PORI_Scoa
PORI_Ccla
PORI_Ccan
PORI_Ocar
CTEN_Baby
CTEN_Mlei
CTEN_Pbac
CTEN_Vmul
CTEN_Edun
OUTC_Mbre
OUTC_Sros
86
100
100
100
84
100
100
100
100
100
100
100
100
100
100
100
100
80
100
100
100
100
100
97
80
96
100
100
79
100
100
100
100
100
100
100

**NON-BILATERIA**				
Ctenophora [5]	Beroe abyssicola	CTEN_Baby	Transcriptome (Illumina)	SRR777787
	Euplokamis dunlapae	CTEN_Edun	Transcriptome (Illumina)	SRR777663
	Mnemiopsis leidyi	CTEN_Mlei	Genome	PRJNA64405
	Pleurobrachia bachei	CTEN_Pbac	Genome	PRJNA213480
	Vallicula multiformis	CTEN_Vmul	Transcriptome (Illumina)	SRR786489
Porifera [14]	Amphimedon queenslandica	PORI_Aque	Genome	PRJNA66531
	Aphrocallistes vastus	PORI_Avas†	Transcriptome (Illumina)	SRR1068281
	Chondrilla caribensis	PORI_Cnuc	Transcriptome (Illumina)	https://dataverse.harvard.edu/dataverse/spotranscriptomes
	Clathrina coriacea	PORI_Ccla†	Transcriptome (Illumina)	SRR3417192
	Cliona varians	PORI_Cvar	Transcriptome (Illumina)	SRR1391011
	Corticium candelabrum	PORI_Ccan	Transcriptome (Illumina)	SRR504694
	Crella elegans	PORI_Cele	Transcriptome (Illumina)	SRR648671
	Ircinia fasciculata	PORI_Ifas	Transcriptome (Illumina)	https://dataverse.harvard.edu/dataverse/spotranscriptomes
	Oscarella carmela	PORI_Ocar	Genome	SRX386229
	Petrosia ficiformis	PORI_Pfic	Transcriptome (Illumina)	SRR504688
	Pseudospongosorites suberitoides	PORI_Psub	Transcriptome (Illumina)	https://dataverse.harvard.edu/dataverse/spotranscriptomes
	Spongilla lacustris	PORI_Slac	Transcriptome (Illumina)	SRR1168575
	Sycon ciliatum	PORI_Scil	Transcriptome (Illumina)	ERR466762
	Sycon coactum	PORI_Scoa	Transcriptome (Illumina)	SRR504689,SRR504690
Placozoa [1]	Trichoplax adhaerens	PLAC_Tadh	Genome	PRJNA12874
Cnidaria [8]	Abylopsis tetragona	CNID_Atet	Transcriptome (Illumina)	SRR871525
	Acropora digitifera	CNID_Adig	Genome	PRJDA67425
	Craspedacusta sowerbyi	CNID_Csow	Transcriptome (Illumina)	SRR923472
	Gorgonia ventalina	CNID_Gven	Transcriptome (Illumina)	SRR935083
	Hydra vulgaris	CNID_Hvul	Genome	PRJNA31231
	Nematostella vectensis	CNID_Nvec	Genome	PRJNA19965
	Polypodium hydriforme	CNID_Phyd	Transcriptome (Illumina)	SRR1336770
	Stomolophus meleagris	CNID_Smel	Transcriptome (Illumina)	SRR1168418
**XENACOELOMORPHA**				
Acoelomorpha [8]	Convolutriloba macropyga	ACOE_Conv	Transcriptome (Illumina)	SRR2681679
	Diopisthoporus longitubus	ACOE_Diol	Transcriptome (Illumina)	SRR3105704
	Diopisthoporus gymnopharyngeus	ACOE_Diop	Transcriptome (Illumina)	SRR3105703
	Eumecynostomum macrobursalium	ACOE_Eumc†	Transcriptome (Illumina)	SRR3105705
	Hofstenia miamia	ACOE_Hmia	Transcriptome (Illumina)	SRR1208932
	Isodiametra pulchra	ACOE_Isop	Transcriptome (Illumina)	SRR2681926
	**Neochildia fusca**	**ACOE_Nfus**	**Transcriptome (Illumina)**	**SRR8617822**
	Symsagittifera roscoffensis	ACOE_Sros	Transcriptome (Illumina)	SRR827579
Nemertodermatida [4]	Ascoparia sp. BV-2015	NEMO_Asco	Transcriptome (Illumina)	SRR2682154
	**Flagellophora sp. Bocas**	**NEMO_Flag†**	**Transcriptome (Illumina)**	**SRR8641368**
	Meara stichopi	NEMO_Mers	Transcriptome (Illumina)	SRR2681155
	Nemertoderma westbladi	NEMO_Nemw	Transcriptome (Illumina)	SRR2682004
Xenoturbellida [1]	**Xenoturbella bocki**	**XENO_Xboc†**	**Transcriptome (Illumina)**	**SRR8638116**
	Xenoturbella bocki	XENO_XbJC	Transcriptome (Illumina)	SRR2681987
**DEUTEROSTOMIA**				
Echinodermata [11]	Apostichopus japonicus	ECHI_Ajap	Transcriptome (Illumina)	SRR1185973
	Echinaster spinulosus	ECHI_Espi	Transcriptome (Illumina)	SRR1139455

	Eucidaris tribuloides	ECHI_Etri	Transcriptome (Illumina)	SRR1138704
	Holothuria glaberrima	ECHI_Hgla	Transcriptome (Illumina)	SRR490924
	Lytechinus variegatus	ECHI_Lvar	Genome	SRX056025
	Ophiocoma echinata	ECHI_Oech	Transcriptome (Illumina)	SRR1138707
	Ophioderma longicauda	ECHI_Olon	Transcriptome (Illumina)	SRR1325052
	Oxycomanthus japonicus	ECHI_Ojap	Transcriptome (Illumina)	SRR1138706
	Patiria miniata	ECHI_Pmin	Genome	SRX096949
	Sclerodactyla briareus	ECHI_Sbria	Transcriptome (Illumina)	SRR1139189
	Strongylocentrotus purpuratus	ECHI_Spur	Genome	SRX130692
Hemichordata [5]	Cephalodiscus gracilis	HEMI_Cgra	Transcriptome (Illumina)	SRR1695473
	Ptychodera bahamensis	HEMI_Pbah	Transcriptome (Illumina)	SRR1695458
	Saccoglossus kowalevskii	HEMI_Skow	Genome	GCA_000003605.1
	Schizocardium cf. braziliense	HEMI_Sbra	Transcriptome (Illumina)	SRR1695467
	Torquaratoridae sp. Antarctica	HEMI_Tbah	Transcriptome (Illumina)	SRR1695469
Cephalochordata [2]	Asymmetron lucayanum	CEPH_Aluc	Transcriptome	SRR1138336
	Branchiostoma floridae	CEPH_Bflo	Genome	PRJNA33245
Urochordata [2]	Ciona intestinalis	UROC_Cint	Genome	PRJNA187185
	Oikopleura dioica	UROC_Odio†	Genome	ASM20953v1
Craniata [7]	Callorhincus milii	CRAN_Cmil	Transcriptome (Illumina)	SRR514104
	Danio rerio	CRAN_Drer	Genome	PRJNA13922
	Gallus gallus	CRAN_Ggal	Genome	PRJNA10808
	Homo sapiens	CRAN_Hsap	Genome	PRJNA178030
	Latimera chalumnae	CRAN_Lcha	Genome	GCA_000225785.1
	Mus musculus	CRAN_Mmus	Genome	PRJNA169
	Petromyzon marinus	CRAN_Pmar	Genome	GCA_000148955.1
**SPIRALIA**				
Chaetognatha [2]	**Sagitta elegans**	**CHAE_Selg**	**Transcriptome (Illumina)**	**SRR8627919**
	**Spadella sp.**	**CHAE_Spdl**	**Transcriptome (Illumina)**	**PRJNA531448**
Bryozoa [4]	**Bugula stolonifera**	**BRYO_Bstl**	**Transcriptome (Illumina)**	**PRJNA531447**
	**Flustrellidra corniculata**	**BRYO_Fcor**	**Transcriptome (Illumina)**	**PRJNA531442**
	**Heteropora pacifica**	**BRYO_Hpac**	**Transcriptome (Illumina)**	**PRJNA531441**
	Membranipora membranacea	BRYO_Mmem	Transcriptome (Illumina)	SRR2131259
Entoprocta [4]	Barentsia gracilis	ENTO_Bgra	Transcriptome (Illumina)	SRR1611554
	**Loxomitra sp.**	**ENTO_Lxmt†**	**Transcriptome (Illumina)**	**PRJNA531440**
	Loxosoma pectinaricola	ENTO_Loxp	Transcriptome (Illumina)	SRR1611559
	**Pedicellina sp. FHL**	**ENTO_PclF†**	**Transcriptome (Illumina)**	**PRJNA531438**
Cycliophora [1]	**Symbion cf. americanus**	**CYCL_Symb**	**Transcriptome (Illumina)**	**PRJNA531426**
	Symbion pandora	CYCL_SyRN‡	Transcriptome (Illumina)	SRR3102772
Annelida [16]	Capitella teleta	ANNE_Ctel	Genome	https://metazoa.ensembl.org/Capitella_teleta/Info/Index
	Diurodrilus subterraneus	ANNE_Diur†	Transcriptome (Illumina)	SRR2131612
	Helobdella robusta	ANNE_Hrob	Genome	http://metazoa.ensembl.org/Helobdella_robusta/Info/Index
	Lobatocerebrum sp.	ANNE_Loba†	Transcriptome (Illumina)	SRR2131397
	Lumbricus rubellus	ANNE_Lrub	Transcriptome (Illumina)	SRR923752
	Magelona johnstoni	ANNE_Mjoh	Transcriptome (Illumina)	SRR1222290
	Marphysa bellii	ANNE_Mbel	Transcriptome (Illumina)	SRR1232821,SRR1232833
	Myzostoma seymourcollegiorum	ANNE_Myzo†	Transcriptome (Illumina)	SRR2005822
	Nepthys caeca	ANNE_Ncae	Transcriptome (Illumina)	SRR1232685,SRR1232795

	Owenia fusiformis	ANNE_Ofus†	Transcriptome (Illumina)	SRR1222288
	Paramphinome jeffreysii	ANNE_Pamp	Transcriptome (Illumina)	SRR1257731,SRR1257732
	Phascolopsis gouldii	ANNE_Plcs	Transcriptome (Illumina)	SRR1654498
	Phyllochaetopterus sp.	ANNE_Phyl	Transcriptome (Illumina)	SRR1257898,SRR1257899
	Phylo foetida	ANNE_Pfoe	Transcriptome (Illumina)	SRR1222216
	Scolelepis squamata	ANNE_Ssqu‡	Transcriptome (Illumina)	SRR1222145
	Siphonosoma cumanense	ANNE_Spsm†	Transcriptome (Illumina)	SRR1646441
Mollusca [14]	Chiton olivaceus	MOLL_Cton	Transcriptome (Illumina)	SRX205322
	Crassostrea gigas	MOLL_Cgig	Genome	http://metazoa.ensembl.org/Crassostrea_gigas/Info/Index
	Hydatina physis	MOLL_Hphy	Transcriptome (Illumina)	SRR1505113
	Laevipilina hyalina	MOLL_Lvpl	Transcriptome (Illumina)	SRX644679
	Leptochiton rugatus	MOLL_Lrug	Transcriptome (Illumina)	SRR1611558
	Lottia gigantea	MOLL_Lott	Genome	http://metazoa.ensembl.org/Lottia_gigantea/Info/Index
	Monodonta labio	MOLL_Mlab	Transcriptome (Illumina)	SRR1505119
	Nautilus pompilius	MOLL_Nomp	Transcriptome (Illumina)	SRR5626553
	Neomenia sp.	MOLL_Nmna	Transcriptome (Illumina)	SRX092155
	Octopus vulgaris	MOLL_Ovul	Transcriptome (Illumina)	SRX092193
	Pholidoskepia sp. [formerly Chaetoderı	MOLL_Phol	Transcriptome (Illumina)	SRR1505105
	Proneomenia custodiens	MOLL_Pcus	Transcriptome (Illumina)	SRR1611561
	Solemya velum	MOLL_Svel	Transcriptome (Illumina)	SRR330465
Nemertea [5]	Argonemertes australiensis	NEME_Aaus	Transcriptome (Illumina)	SRX646169
	Baseodiscus unicolor	NEME_Buni	Transcriptome (Illumina)	SRX644738
	Carinoma hamanako	NEME_Cham	Transcriptome (Illumina)	SRX643224
	Hubrechtella ijimai	NEME_Hiji	Transcriptome (Illumina)	SRX644663
	Protopelagonemertes beebei	NEME_Peeb	Transcriptome (Illumina)	SRX646186
Brachiopoda [7]	Glottidia pyramidata	BRAC_Gpyr	Transcriptome (Illumina)	SRR1611555
	Hemithiris psittacea	BRAC_Hpst	Transcriptome (Illumina)	SRR1611556
	Kraussina rubra	BRAC_Krub	Transcriptome (Illumina)	SRR2131392
	Lingula anatina	BRAC_Lana	Transcriptome (Illumina)	SRR330440
	Macandrevia cranium	BRAC_Mcdv‡	Transcriptome (Illumina)	SRR1611130
	Novocrania anomala	BRAC_Nvcn	Transcriptome (Illumina)	SRR1611564
	Terebratalia transversa	BRAC_Ttvs	Transcriptome (Illumina)	SRR2005824
Phoronida [3]	Phoronis australis	PHOR_Paus	Transcriptome (Illumina)	SRR2018856
	Phoronis psammophila	PHOR_Ppsa	Transcriptome (Illumina)	SRR1611565
	Phoronopsis harmeri	PHOR_Phar	Transcriptome (Illumina)	SRR2131255
Gastrotricha [5]	Dactylopodola baltica	GAST_Dpdl	Transcriptome (Illumina)	SRR1273672,SRR1273673,SRR1275388,SRR1275389
	Diuronotus aspetos	GAST_Dnot	Transcriptome (Illumina)	SRR2131262
	Lepidodermella squamata	GAST_Lepi	Transcriptome (Illumina)	SRR1982110
	Macrodasys sp.	GAST_Mdas	Transcriptome (Illumina)	SRR1271706,SRR1271707,SRR1271708,SRR1275393
	Megadasys sp.	GAST_Megd	Transcriptome (Illumina)	SRR1273711,SRR1273712,SRR1275394,SRR1275397
Platyhelminthes [11]	Bothrioplana semperi	PLAT_Bsem	Transcriptome (Illumina)	SRR1955240, SRR1796356
	Echinococcus multilocularis	PLAT_Ecmu	Genome	http://www.genedb.org/Homepage/Emultilocularis
	Geocentrophora applanata	PLAT_Gapp	Transcriptome (Illumina)	SRR1955490
	Gnosonesimida sp. IV	PLAT_Gnos	Transcriptome (Illumina)	SRR1976178, SRR1976442
	Microstomum cf. lineare	PLAT_Mlin	Transcriptome (Illumina)	SRR1980039
	Monocelis fusca	PLAT_Mfus	Transcriptome (Illumina)	SRR1979673
	Prostheceraeus vittatus	PLAT_Pvit	Transcriptome (Illumina)	SRR2000268

	Rhynchomesostoma rostratum	PLAT_Rros	Transcriptome (Illumina)	SRR1980143
	Schistosoma mansoni	PLAT_Sman	Genome	http://www.genedb.org/Homepage/Smansoni
	Schmidtea mediterranea	PLAT_Smed	Transcriptome (Illumina)	Alejandro Sanchéz Alvarado and Eric Ross, as in 10.7554/eLife.05503
	Stenostomum leucops	PLAT_Sleu	Transcriptome (Illumina)	SRR1910423
Gnathostomulida [3]	Austrognathia sp.	GNAT_Augn	Transcriptome (Illumina)	SRR1976176
	Gnathostomulidae sp. [incorrectly ID'd	GNAT_Gnat†	Transcriptome (Illumina)	SRR1271607,SRR1271608,SRR1271613,SRR1275390
	**Tenuignathia rikerae**	**GNAT_Trik**	**Genome**	**PRJNA525844**
Micrognathozoa [1]	Limnognathia maerski	MICR_Limn	Transcriptome (Illumina)	SRR2131287
Rotifera [5]	Adineta vaga	ROTI_Avag	Genome	https://metazoa.ensembl.org/Adineta_vaga/Info/Index
	Brachionus calyciflorus	ROTI_Bcal	Transcriptome (Illumina)	SRR611718,SRR611719,SRR611720,SRR620051,SRR620163
	Echinorhynchus gadi	ROTI_Egad†	Transcriptome (Illumina)	SRR2131254
	Macracanthorhynchus hirudinaceus	ROTI_Mhir	Transcriptome (Illumina)	ERR454503,ERR454504
	Rotaria rotatoria	ROTI_Rrot	Transcriptome (Illumina)	ERR454505
Dicyemida [1]	**Dicyemida sp.**	**DICY_Dicy‡**	**Transcriptome (Illumina)**	**PRJNA531422**
**ECDYSOZOA**				
Priapulida [3]	**Meiopriapulus fijiensis**	**PRIA_Meio**	**Transcriptome (Illumina)**	**PRJNA531413**
	Priapulus caudatus	PRIA_Pcau	Transcriptome (Illumina)	SRR1611567
	Tubiluchus corallicola	PRIA_Tubi‡	Transcriptome (Illumina)	PRJNA523693
Loricifera [1]	Armorloricus sp.	LORI_Arlc*	Transcriptome (Illumina)	SRR2131253
Kinorhyncha [1]	**Echinoderes dujardinii**	**KINO_Eduj***	**Transcriptome (Illumina)**	**PRJNA523695**
	Pycnophyes kielensis	KINO_Pkie*	Transcriptome (454)	SRR1141803
Nematomorpha [1]	**Nectonema munidae**	**NEMM_Nect***	**Transcriptome (Illumina)**	**PRJNA523701**
Nematoda [8]	Caenorhabditis elegans	NEMA_Celg	Genome	http://useast.ensembl.org/info/data/ftp/index.html
	Haemonchus contortus	NEMA_Hcon	Genome	ftp://ftp.sanger.ac.uk/pub/pathogens/Haemonchus/contortus
	Loa loa	NEMA_Lloa	Genome	https://metazoa.ensembl.org/Loa_loa/Info/Index
	Panagrellus redivivus	NEMA_Pred	Genome	https://parasite.wormbase.org/Panagrellus_redivivus_prjna186477/Info/Index/
	Pristionchus pacificus	NEMA_Ppac	Genome	https://metazoa.ensembl.org/Pristionchus_pacificus/Info/Index
	Romanomermis culicivorax	NEMA_Rmcl	Genome	https://parasite.wormbase.org/Romanomermis_culicivorax_prjeb1358/Info/Index/
	Trichinella spiralis	NEMA_Tspr	Genome	http://metazoa.ensembl.org/Trichinella_spiralis/Info/Index
	Trichuris muris	NEMA_Tmur	Genome	https://parasite.wormbase.org/Trichuris_muris_prjeb126/Info/Index/
Tardigrada [2]	Hypsibius dujardini	TARD_Hduj	Genome	http://ensembl.tardigrades.org/Hypsibius_dujardini_nhd231/Info/Index
	Milnesium tardigradum	TARD_Mtar	Transcriptome (Hybrid Illu	Provided by Chong Wang, as in Laumer et al 2015
Onychophora [4]	**Ooperipatellus sp.**	**ONYC_Oope**	**Transcriptome**	**PRJNA523702**
	**Peripatoides sp.**	**ONYC_Peri**	**Transcriptome**	**PRJNA523703**
	Peripatopsis overbergiensis	ONYC_Pcap	Transcriptome	SRR1145776
	**Peripatus sp.**	**ONYC_Pept**	**Transcriptome**	**PRJNA523712**
Arthropoda [14]	**Anoplodactylus insignis**	**ARTH_Ains**	**Transcriptome (Illumina)**	**SRR5237777**
	Apis mellifera	ARTH_Amel	Genome	https://metazoa.ensembl.org/Apis_mellifera/Info/Index
	Calanus finmarchicus	ARTH_Cfin	Transcriptome (Illumina)	SRR6065686
	Daphnia pulex	ARTH_Dpul	Genome	https://metazoa.ensembl.org/Daphnia_pulex/Info/Index
	Drosophila melanogaster	ARTH_Dmel	Genome	https://www.ensembl.org/Drosophila_melanogaster/Info/Index
	Eudigraphis taiwaniensis	ARTH_Etai	Transcriptome (Illumina)	SRR3458640
	Fenneropenaeus merguiensis	ARTH_Fmer	Transcriptome (Illumina)	SRR1756093
	Folsomia candida	ARTH_Fcan	Transcriptome (Illumina)	SRX321913
	Glomeris marginata	ARTH_Gmar	Transcriptome (Illumina)	SRR3233211
	Ixodes scapularis	ARTH_Isca	Genome	http://metazoa.ensembl.org/Ixodes_scapularis/Info/Index
	Limulus polyphemus	ARTH_Limu	Genome	PRJNA187356

	Scutigerella sp.	ARTH_Scuti†	Transcriptome (Illumina)	SRR3458649
	Stegodyphus mimosarium	ARTH_Smim	Genome	https://metazoa.ensembl.org/Stegodyphus_mimosarum/Info/Index
	Strigamia maritima	ARTH_Smar	Genome	https://metazoa.ensembl.org/Strigamia_maritima/Info/Index
**OUTGROUPS**				
Choanoflagellatea [2]	Monosiga brevicollis	OUTC_Mbre	Genome	GCA_000002865.1 V1.0
	Salpingoeca rosetta	OUTC_Sros	Genome	ACSY01
Filastera [2]	Capsaspora owczarzaki	OUTI_Cowc†	Genome	GCA_000151315.2
	Ministeria vibrans	OUTI_Mvar†	Transcriptome (Illumina)	SRX096925
Cristidiscoidea [1]	Fonticula alba	OUTI_Falb†	Genome	GCA_000388065.2
Ichthyosporea [2]	Amoebidium parasiticum	OUTI_Apar†	Transcriptome (Illumina)	PRJNA189477
	Sphaeroforma artica	OUTI_Sart†	Genome	GCA_001186125.1
Apusomonadida [1]	Thecamonas trahens	OUTI_Ttra†	Genome	GCF_000142905.1
Ascomycota (Fungi) [1]	Fusarium oxysporum	OUTF_Foxy†	Genome	AGNE01
Glomeromycota (Fungi) [2]	Rhizophagus irregularis	OUTF_Rirr/OUTF_Rsol [re	Genome	JEMT01
	Spizellomyces punctatus	OUTF_Spun†	Genome	GCF_000182565.1
Blastocladiomycota (Fungi) [1]	Allomyces macrogynus	OUTF_Amac†	Genome	GCA_000151295.1
Cryptomycota (Fungi) [1]	Rozella allomycis	OUTF_Rall†	Genome	PRJNA81749
Zygomycota (Fungi) [1]	Mortierella verticillata	OUTF_Mver†	Genome	GCA_000739165.1
